# Lactate controls glomerular endothelial barrier integrity in lupus nephritis

**DOI:** 10.1172/jci.insight.205034

**Published:** 2026-08-18

**Authors:** Jiaxin Lei, Xingyu Zhai, Yixin Wang, Ying Li, Lei Li, Mengdi Liu, Jing Guo, Lingyi Li, Zhezhuyun Chen, Qinghua Cao, Zhichun Liu, Ting Liu, Lin Xu, Zhenke Wen

**Affiliations:** 1 Department of Pulmonary and Critical Care Medicine at the First Affiliated Hospital of Soochow University, Institutes of Biology and Medical Sciences, School of Basic Medical Sciences, Suzhou Medical College of Soochow University, Suzhou, China.; 2 Jiangsu Key Laboratory of Infection and Immunity, MOE Key Laboratory of Geriatric Diseases and Immunology, Biomedical Basic Research Center of Jiangsu Province, Soochow University, Suzhou, China,; 3 Department of Rheumatology, The Second Affiliated Hospital of Soochow University, Soochow University, Suzhou, China.; 4 School of Biomedical Sciences, University of Leeds, Leeds, United Kingdom.; 5 Renal Medicine, Kolling Institute of Medical Research, Royal North Shore Hospital, St Leonards, Sydney Medical School, University of Sydney, St Leonards, New South Wales, Australia.; 6 Department of Rheumatology, The Affiliated Wuxi People’s Hospital of Nanjing Medical University, Wuxi People’s Hospital, Wuxi Medical Center, Nanjing Medical University, Wuxi, China.; 7 Key Laboratory of Cancer Prevention and Treatment of Guizhou Province, Department of Immunology, Zunyi Medical University, Zunyi, China.

**Keywords:** Autoimmunity, Nephrology, Lupus

## Abstract

Systemic lupus erythematosus (SLE) is a progressive autoimmune disease that affects multiple organs and tissues, with lupus nephritis (LN) as one of its most severe complications. Although LN progression is associated with compromised permeability of human renal glomerular endothelial cells (HRGECs), the underlying mechanisms are not fully defined. Herein, we demonstrate that aberrant glycolysis drives this glomerular endothelial barrier defect by suppressing the transcription of tight junction (TJ) genes. Mechanistically, circulating self-DNA in SLE plasma acts as a ligand that activates the cGAS/STING pathway in HRGECs, driving aberrant glycolytic adaption. The resulting glycolytic product, lactate, serves as a substrate for protein lactylation, leading to extensive lactylation and subsequent ubiquitination of enhancer of zeste homolog 2 (EZH2). In consequence, EZH2 deficiency results in reduced H3K27me3 levels, thereby suppressing the transcription of TJ genes. In a self-DNA–induced SLE model, inhibition of cGAS/STING signaling or lactate production effectively restored the integrity of TJs of HRGECs and concurrently alleviated key LN symptoms. Together, lactate programs lactylation and ubiquitination of EZH2 to impair the glomerular endothelial barrier in human SLE.

## Abstract 2

Lactate programs lactylation and ubiquitination of EZH2 to impair glomerular endothelial barrier in human SLE

## Graphical abstract

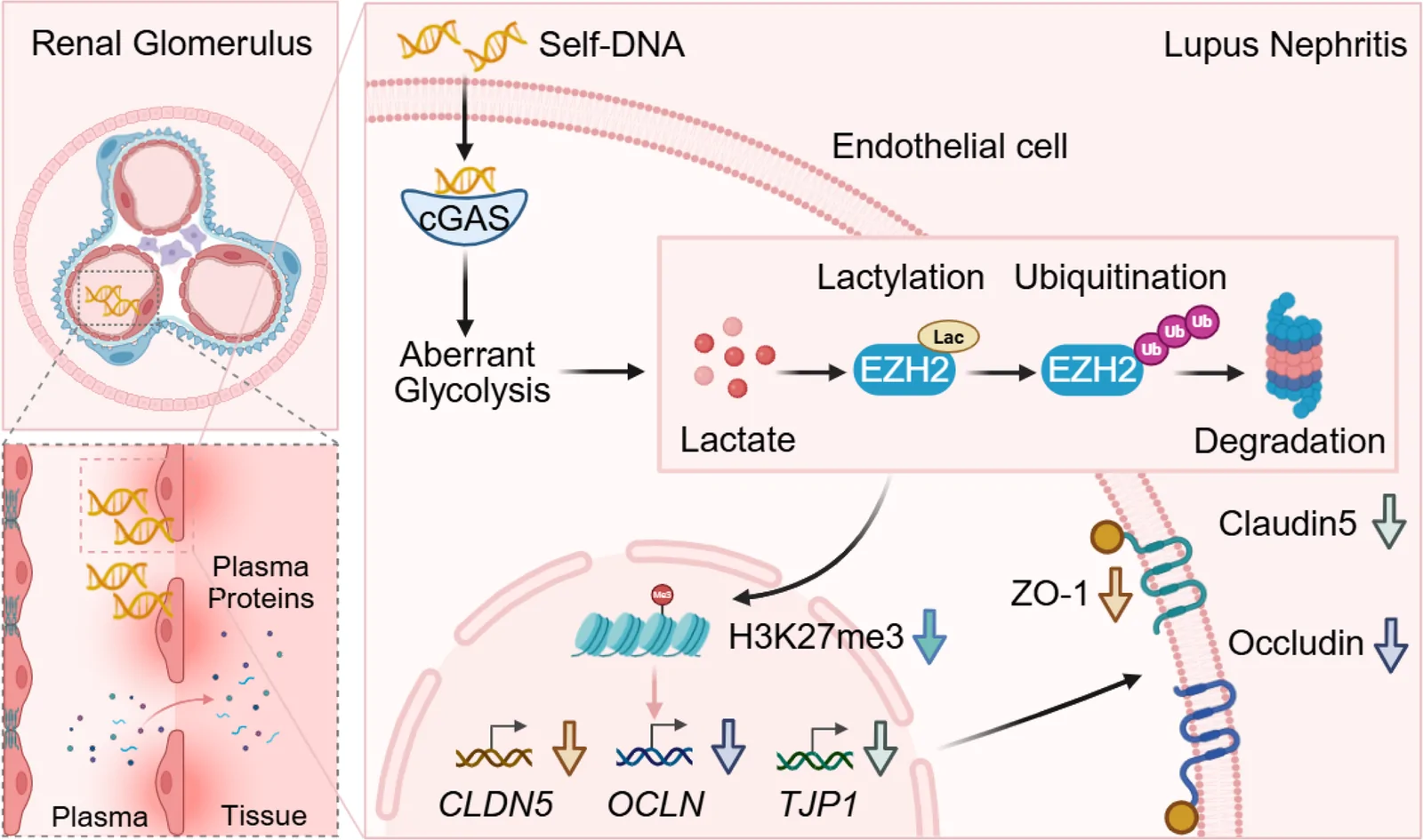

## Introduction

Systemic lupus erythematosus (SLE) is a common and multisystem autoimmune disorder with limited treatment options. It is marked by diverse clinical presentations and a tendency to cause progressive organ damage. Among patients with SLE, roughly 40%–60% develop lupus nephritis (LN) — a serious complication that often advances to end-stage renal disease (1). To date, only 20%–30% of patients with SLE achieve complete remission after 6 months of standard treatment, and this is often accompanied by severe adverse reactions, which further worsen LN prognosis (2). In recent years, biological agents targeting autoimmune responses and inflammatory pathways have emerged and advanced rapidly (3). However, their limited response rates and difficulty targeting specific organs mean there is an urgent need for new approaches for LN treatment.

A key clinical feature of LN is the breakdown of the glomerular filtration barrier (GFB), which results in measurable proteinuria. This barrier consists of 3 distinct layers, all formed by renal resident cells: endothelial cells (ECs), the glomerular basement membrane, and the foot processes of podocytes (4). Growing evidence points to endothelial dysfunction as a key driver behind severe proteinuria in patients with LN (5, 6). This is largely because human renal glomerular ECs (HRGECs) form the innermost layer of glomerular capillaries, leaving them directly exposed to circulating pathogenic factors, including immunogenic self-DNA, immune complexes (ICs), and immune cells (7). These pathogenic interactions exacerbate HRGEC injury and disrupt cellular adhesion (8). Cellular adhesion forms a semipermeable membranous barrier, composed of adjacent tight junctions (TJs), adherens junctions, and a range of adhesion molecules (8). Although it is not the sole factor, the structural integrity of the GFB depends heavily on the formation of TJs. Under physiological conditions, ECs constitutively express ZO-1, occludin, and claudin5 to contribute to TJ architecture, maintaining passive paracellular transport (9). Although dysregulation of TJ proteins has been established to be related to diabetic nephropathy–associated albuminuria (10), their expression patterns and functional changes in LN are still not well understood.

Autoantibodies are the most reliable serological markers for SLE, found in 70%–80% of patients (11). Accumulation of self-antigens induces excessive activation of T and B cells, thereby promoting plasma cell–derived autoantibody production, which constitutes a core paradigm of autoimmune pathogenesis (12). Over the past 20 years, the immunogenic self-DNA hypothesis has uncovered the mechanistic basis for the specific generation of anti-dsDNA autoantibodies in SLE (13). In this context, self-DNA orchestrates immune responses through 3 distinct but interconnected modes. First, self-DNA engulfed by antigen-presenting cells (APCs) promotes the differentiation of Tfh cells, which support germinal center B cell hypermutation and the production of anti-dsDNA IgG (14, 15). Second, cytoplasmic self-DNA is sensed by intracellular DNA sensors, thereby regulating cell fate and contributing to cellular dysfunction (16). Third, self-DNA binds to anti-dsDNA antibodies to form ICs; tissue deposition of these immunogenic ICs along with self-DNA further initiates and exacerbates local proinflammatory cascades (2, 17). Although these pathogenic events are well characterized, the direct regulatory effects of circulating self-DNA and ICs on tissue-resident cells — particularly glomerular ECs — remain incompletely defined. Accordingly, we propose that circulating self-DNA disrupts TJ integrity in HRGECs via downstream DNA-sensing cascades, ultimately facilitating the progression of LN.

In this study, we found that the expression of TJ-related genes is reduced in SLE cases, and this is mainly mediated by self-DNA in SLE plasma. Patient-derived circulating DNA is sensed by cGAS, which reprograms HRGEC metabolism to enhance glycolysis, leading to lactate accumulation. The excess lactate acts as a substrate for enhancer of zeste homolog 2 (EZH2) lactylation, which in turn promotes EZH2 ubiquitination and degradation. When EZH2 is depleted, H3K27me3 levels drop; this ultimately suppresses the transcription of TJ genes and increases the permeability of the glomerular capillary wall. Targeting EZH2 lactylation and cGAS reversed typical LN symptoms, such as proteinuria, glomerular IgG deposition, and elevated serum anti-dsDNA antibodies. This suggests our findings offer a promising therapeutic strategy for LN.

## Results

### Plasma from patients with SLE disrupts TJ barriers and elevates permeability in HRGECs.

**Figure 1:**
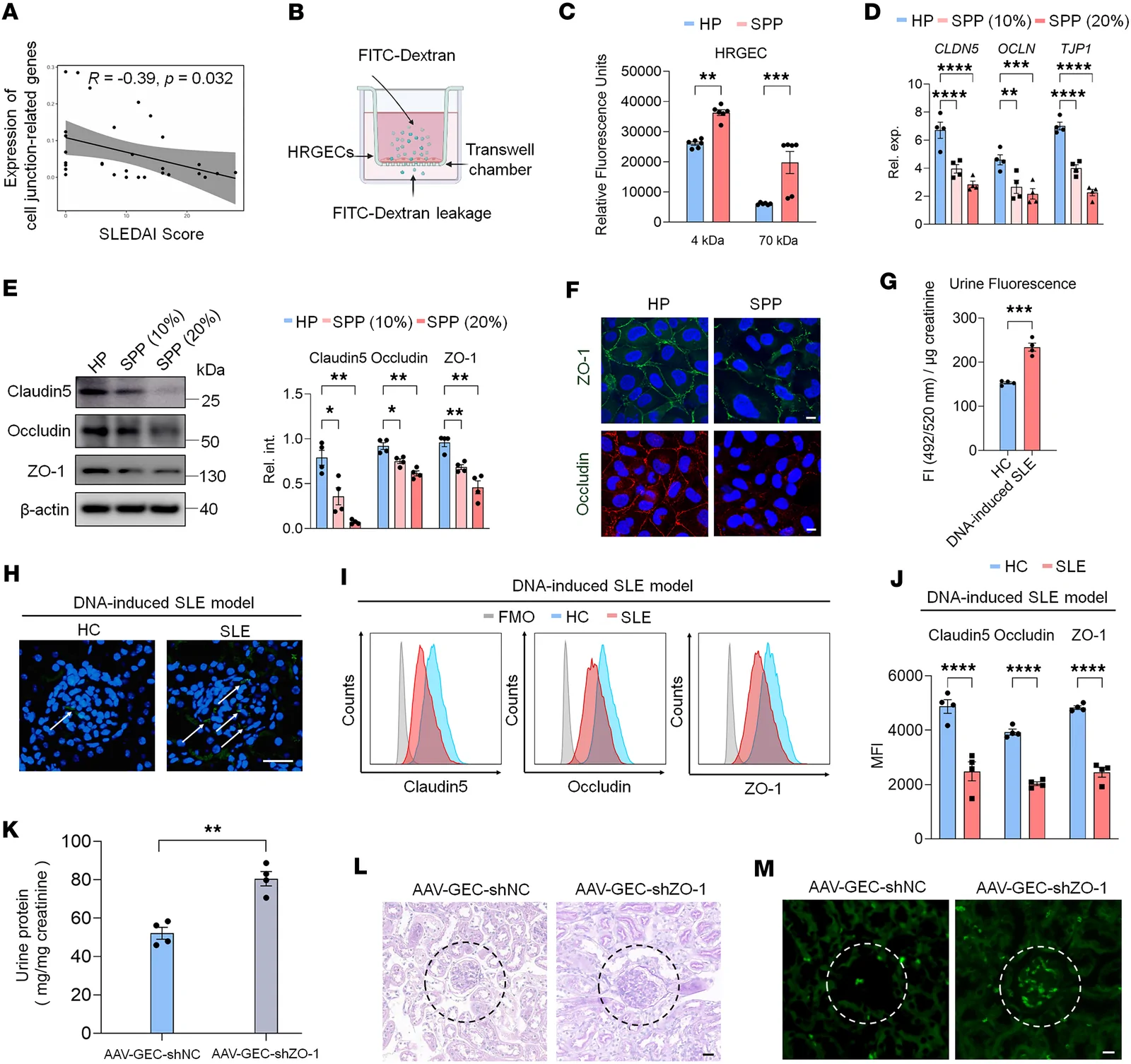
SLE plasma impairs the TJ barrier in HRGECs. (**A**) The correlation between the expression of TJ-related genes and SLEDAI scores in patients with SLE. (**B**) Schematic diagram for experimental design. (**C**) HRGECs were treated with plasma from HCs or patients with SLE for 24 hours. The fluorescence intensity of 4 kDa and 70 kDa FITC-dextran in the lower chamber was measured using a microplate reader. Mean ± SEM from 6 individuals in each group. (**D**–**F**) ECs were cultured with 10% or 20% plasma from HCs and patients with SLE. TJ-associated mRNA and protein levels were assessed by qPCR (**D**, 10%/20% plasma), Western blot (**E**, 10%/20% plasma), and immunofluorescence staining (**F**, 10% plasma). Data are mean ± SEM (*n* = 4 individuals per group for **D**–**E**). Scale bar: 10 μm (**F**). (**G**) Urinary 4 kDa FITC-dextran fluorescence intensity normalized to creatinine was measured via microplate reader in healthy and SLE model mice. Values are presented as mean ± SEM; 4 mice per group. (**H**) Localization of 70 kDa FITC-dextran in the glomeruli of healthy mice and SLE model mice was visualized by immunofluorescence microscopy. Scale bar: 50 μm. (**I** and **J**) Expression of TJ-associated proteins in glomerular ECs in vivo from healthy and SLE model mice. Data are presented as mean ± SEM; 4 mice per group. (**K**–**M**) Healthy mice were subjected to treatment with AAV2-GEC-shZO-1 or negative control shRNA. (**K**) Urinary protein levels. (**L**) Periodic acid–Schiff (PAS) staining of renal tissues. (**M**) Renal IgG deposition. Data are mean ± SEM (*n* = 4 mice per group for **K**). Scale bar: 20 μm. *P < 0.05, **P < 0.01, ***P < 0.001, and ****P < 0.0001 with unpaired 2-tailed *t* test (**G** and **K**), 2-way ANOVA plus Tukey’s method (**C**–**E**, and **J**), and Pearson’s correlation statistical analysis (**A**).

To explore the possible correlations between the integrity of the glomerular paracellular permeability barrier and LN progression, we analyzed clinical data from the NCBI’s Gene Expression Omnibus (GEO). Notably, we observed a negative correlation between renal cell junction–related protein expression levels and SLE disease activity index (SLEDAI) scores, implying that altered paracellular permeability may play a critical role in LN pathogenesis (Figure 1A). Since plasma isolated from patients with SLE recapitulates pathological phenotypes in renal cells in vitro (2), we assessed the permeability of HRGECs — cells directly exposed to circulating plasma — via FITC-dextran Transwell permeability assays. Our results showed a marked increase in fluorescence in the lower chamber of wells treated with SLE patient–derived plasma compared with healthy controls (HCs) (Figure 1, B and C), indicating an enhanced paracellular leakage. Importantly, cell confluency and viability did not differ between the 2 plasma treatment groups (Supplemental Figure 1, A and B; supplemental material available online with this article; https://doi.org/10.1172/jci.insight.205034DS1), ruling out potential broad cytotoxicity or growth interference. Next, we separately examined the expression of genes related to TJs, adherens junctions, and fenestration and found that ECs treated with SLE plasma exhibited selective downregulation of TJ-related proteins including claudin5, occludin, and ZO-1, whereas VE-cadherin (a core adherens junctions component) and plasmalemma vesicle-associated protein (PLVAP, the key mediator of endothelial fenestration) remained unchanged (Figure 1, D–F, and Supplemental Figure 1, C–E). This phenomenon was validated in HUVECs, confirming the reliability of our conclusion that SLE patient–derived plasma impairs the TJ barrier (Supplemental Figure 1, F–H).

In order to investigate alterations in HRGEC permeability during LN pathogenesis in vivo, we established a murine LN model via self-DNA immunization as previously reported (13). These mice exhibited typical LN manifestations, including elevated serum anti-dsDNA antibodies, proteinuria, albuminuria, glomerular lesions, and renal IgG deposition (Supplemental Figure 2, A–E). Notably, we detected markedly increased urinary leakage of 4 kDa FITC-dextran and prominent glomerular accumulation of 70 kDa FITC-dextran, indicating impaired glomerular endothelial barrier function during disease progression (Figure 1, G and H). We further isolated renal glomeruli using the differential adhesion assay (18) and performed immunostaining for the endothelial marker CD31, as well as the TJ proteins ZO-1, occludin, and claudin5. Consistent with our in vitro findings, glomerular endothelial TJ architecture was severely disrupted as SLE progressed (Figure 1, I and J). These results provide direct in vivo evidence that TJ barrier dysfunction occurs in HRGECs during LN.

To further clarify the pathogenic role of TJ barrier disruption in LN progression, we generated AAV2 vectors modified with the glomerular endothelium–targeted peptide QVLVYRE (19). ZO-1 shRNA was packaged into these vectors to achieve specific ZO-1 knockdown in glomerular ECs in vivo. After glomerular isolation, we validated the knockdown efficiency of ZO-1 via flow cytometry using CD31 and ZO-1 costaining (Supplemental Figure 2F). Functional assays demonstrated that disruption of endothelial TJs induced by ZO-1 silencing alone was sufficient to recapitulate hallmark SLE pathologies, including severe proteinuria, renal IgG deposition, and extensive glomerular injury (Figure 1, K–M).

### SLE patient–derived plasma reprograms HRGEC metabolism to glycolysis, thereby increasing endothelial permeability.

**Figure 2:**
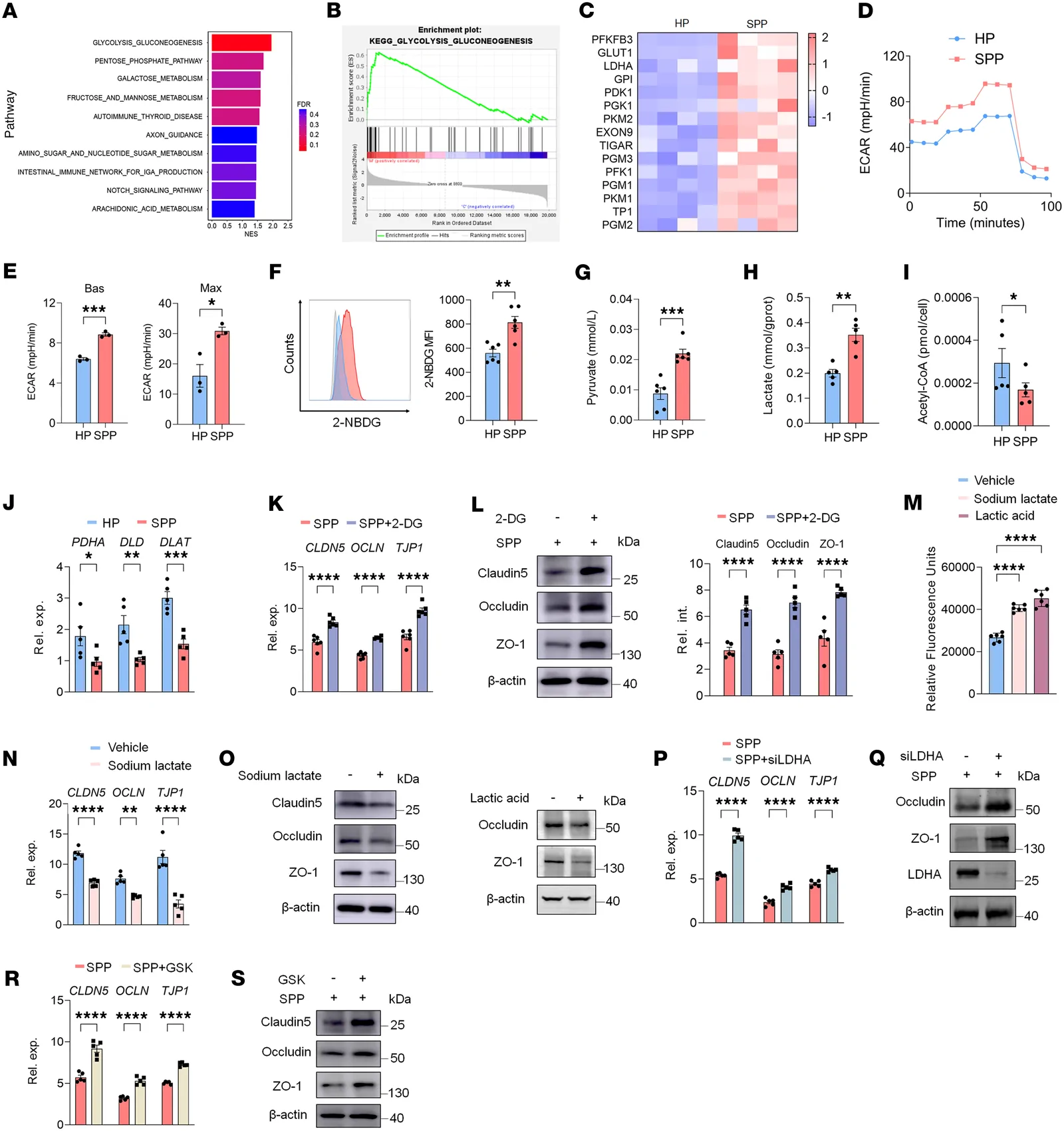
SLE plasma impairs the TJ barrier in HRGECs through glycolytic shift–driven lactate accumulation. (**A** and **B**) Gene Set Enrichment Analysis (GSEA) and Kyoto Encyclopedia of Genes and Genomes (KEGG) pathway analysis were performed on RNA-seq data derived from HRGECs treated with 10% plasma from HCs or patients with SLE. NES, normalized enrichment score. (**C**–**E**) ECs were treated with 10% plasma from HCs or patients with SLE for 24 hours. qPCR was performed to detect the mRNA expression of glycolysis-related genes (**C**), and Seahorse assay was used to measure ECAR (**D** and **E**). Data are mean ± SEM (3 individuals/group for **E**). (**F**) ECs were treated with 10% plasma derived from HCs or patients with SLE for 24 hours, and 2-NBDG levels were determined by flow cytometry. Mean ± SEM (6 individuals/group). (**G**–**I**) After 24 hours of treatment with SLE- or HC-derived plasma (10%), ECs were analyzed for pyruvate (**G**), lactate (**H**), and acetyl-CoA (**I**) using corresponding assay kits. Mean ± SEM (5–6 individuals/group). (**J**) After treatment with HC or SLE plasma (10%), gene expression of pyruvate dehydrogenase complex components in ECs was detected by qPCR. Mean ± SEM (5 individuals/group). (**K** and **L**) mRNA and protein levels of TJ-related proteins after SLE plasma treatment (10%) with or without 2-DG (1 mM) for 24 hours analyzed by qPCR (**K**) and Western blot (**L**). Mean ± SEM (5–6 individuals/group). (**M**) ECs were treated with 40 mM lactate or 10 mM lactic acid for 24 hours. Fluorescence intensity of 4 kDa FITC-dextran in the lower chamber was quantified via a microplate reader. Mean ± SEM (6 independent experiments/group). (**N** and **O**) After treatment with lactate (40 mM) or lactic acid (10 mM), mRNA and protein levels of TJ-related proteins in ECs were assessed by qPCR (**N**) and Western blot (**O**), respectively. Mean ± SEM (5 independent experiments/group). (**P** and **Q**) ECs were transfected with siRNA targeting LDHA and treated with 10% SLE plasma for 24 hours. mRNA and protein levels of TJ-related proteins were analyzed by qPCR (**P**) and Western blot (**Q**). Mean ± SEM (5 individuals/group). (**R** and **S**) ECs were treated with 10% SLE plasma with GSK2837808A (40 μM), and corresponding mRNA and protein levels were determined by qPCR (**R**) and Western blot (**S**), respectively. Mean ± SEM (5 individuals/group). *P < 0.05, **P < 0.01, ***P < 0.001, and ****P < 0.0001 with unpaired 2-tailed *t* test (**E**–**I**), 1-way (**M**) or 2-way ANOVA plus Tukey’s method (**J**–**L**, **N**, **P**, and **R**).

To explore why plasma from patients with SLE increases permeability in HRGECs, we conducted RNA-seq analysis comparing HRGECs pretreated with patients’ plasma versus those exposed to HC samples. Our results showed that SLE plasma considerably upregulated the glycolytic metabolism pathway (Figure 2, A and B). Further analysis revealed that SLE plasma upregulated glycolysis-related genes while suppressing oxidative phosphorylation (OXPHOS) (Figure 2C and Supplemental Figure 3). We next assessed glycolytic capacity in HRGECs after SLE plasma pretreatment by measuring the extracellular acidification rate (ECAR). Cells exposed to SLE plasma exhibited notably enhanced glycolytic capacity compared with those treated with HC plasma (Figure 2, D and E). To quantify cellular glucose uptake and pinpoint altered glycolytic influx, we performed 2-(N-(7-nitrobenz-2-oxa-1,3-diazol-4-yl) amino)-2-deoxyglucose (2-NBDG) uptake assays; 2-NBDG is a fluorescent glucose analog that competes with native glucose for transmembrane transport and accumulates intracellularly upon uptake (20). Accordingly, elevated fluorescent intensity in SLE plasma–pretreated ECs (Figure 2F) indicates enhanced glucose import, reflecting upregulated glycolytic substrate influx. Pyruvate, a central glycolytic node, showed divergent metabolic fates: its conversion to lactate was augmented, while acetyl-CoA production was constrained in SLE plasma–treated ECs (Figure 2, G–I). This metabolic shift correlated with downregulated expression of pyruvate dehydrogenase complex components (Figure 2J).

Pharmacological inhibition of glycolysis with 2-deoxy-d-glucose (2-DG) reversed the SLE plasma–induced downregulation of TJ genes (Figure 2, K and L). To explore the effect of lactic acid accumulation resulting from this metabolic shift, we observed that exogenous supplementation with either sodium lactate or lactic acid was sufficient to downregulate TJ gene expression (Figure 2, M–O). Importantly, both genetic lactate dehydrogenase A (LDHA) knockdown and pharmacological inhibition of LDHA (GSK2837808A) restored TJ levels (Figure 2, P–S). These results suggest that SLE plasma reprograms EC metabolism to mimic aerobic glycolysis, thereby inducing endothelial barrier dysfunction. Of note, although patients on corticosteroids were not excluded, our analysis confirmed that corticosteroid treatment did not affect plasma or HRGEC lactate levels (Supplemental Figure 4, A and B).

### SLE plasma drives endothelial barrier dysfunction via glycolysis-mediated EZH2 degradation and H3K27me3 reduction.

**Figure 3:**
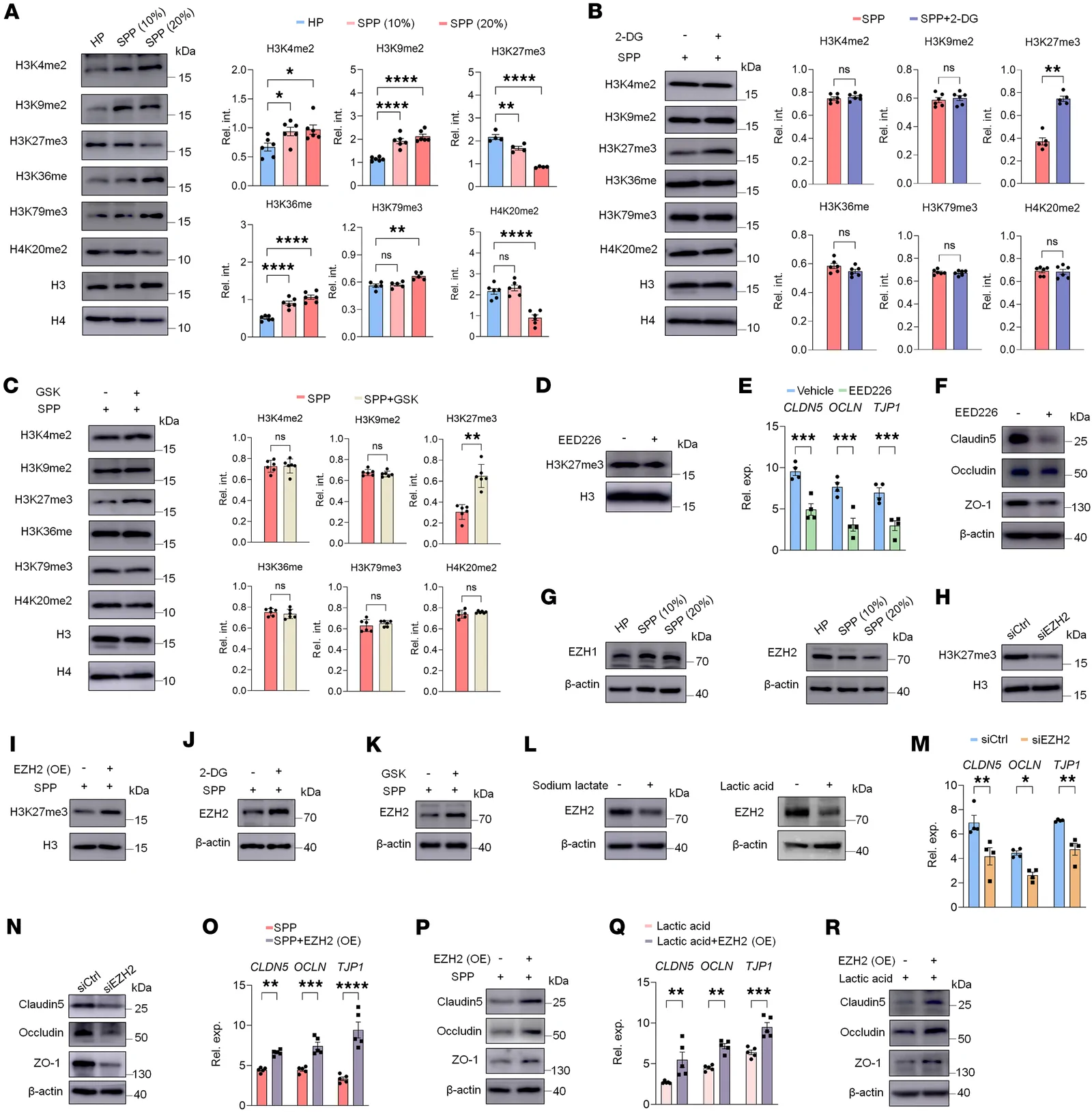
Abnormal glycolysis and subsequent lactate accumulation trigger EZH2 degradation, leading to diminished H3K27me3 abundance. (**A**) Western blot analysis of histone methylation levels in HRGECs after treatment with HC- or SLE-derived plasma (10% or 20%; 24 hours). Mean ± SEM (4–6 individuals/group). (**B** and **C**) ECs were treated with 10% SLE plasma together with 2‑DG (1 mM) or GSK2837808A (40 μM), with quantification of histone methylation levels via Western blot. Mean ± SEM (5–6 individuals/group). (**D**) Western blot analysis of H3K27me3 levels in HRGECs after 24 hours’ treatment with or without EED226 (50 nM). (**E** and **F**) qPCR (**E**) and Western blot (**F**) analyses of TJ-related protein expression in HRGECs after 24 hours’ treatment with or without EED226 (50 nM). Mean ± SEM (*n* = 4 per group). (**G**) ECs were treated for 24 hours with plasma from HCs or patients with SLE at 10% or 20% concentration. Protein levels of EZH1 and EZH2 were analyzed by Western blot. (**H**) Levels of H3K27me3 in ECs assessed by Western blot after EZH2 knockdown. (**I**) ECs were treated with 10% SLE plasma for 24 hours in the presence or absence of EZH2 overexpression. Levels of H3K27me3 were assessed by Western blot. (**J** and **K**) Western blot analysis of EZH2 protein levels in ECs treated with 10% SLE plasma for 24 hours in the presence or absence of 2‑DG (1 mM) or GSK2837808A (40 μM). (**L**) EZH2 protein levels in ECs exposed to lactate (40 mM) or lactic acid (10 mM) for 24 hours assessed by Western blot. (**M** and **N**) qPCR (**M**) and Western blot (**N**) analysis of TJ-related protein expression in ECs after EZH2 knockdown. Mean ± SEM (4 independent experiments/group) (**M**). (**O** and **P**) TJ-related protein expression analyzed by qPCR (**O**) and Western blot (**P**) in ECs upon treatment with 10% SLE plasma with or without EZH2 overexpression. Mean ± SEM, 5 individuals (**O**). (**Q** and **R**) qPCR (**Q**) and Western blot (**R**) analysis of TJ-related protein expression in ECs after lactic acid treatment (10 mM) with or without EZH2 overexpression. Mean ± SEM, 5 independent experiments (**Q**). *P < 0.05, **P < 0.01, ***P < 0.001, and ****P < 0.0001 with 2‑tailed paired *t* test (**B** and **C**) and 1‑way (**A**) and 2‑way ANOVA plus Tukey’s method (**E**, **M**, **O**, and **Q**).

In line with the evidence that histone methylation is linked to heterochromatin formation and the repression of transcription across multiple genes (21), our mass spectrometry analysis demonstrated that plasma from patients with SLE substantially altered histone methylation patterns in ECs. Specifically, we observed higher levels of H3K4me2, H3K9me2, H3K36me, and H3K79me3, alongside lower levels of H3K27me3 and H4K20me2 (Supplemental Figure 5A), findings that were consistently confirmed by Western blot (Figure 3A). To elucidate the potential correlation between metabolic reprogramming induced by SLE plasma and abnormal histone methylation, we utilized 2-DG and GSK2837808A, respectively, and detected histone methylation levels. Our results showed that both 2-DG and GSK2837808A restored H3K27me3 levels, supporting that H3K27me3 is involved in the increased glycolysis of ECs (Figure 3, B and C). To further examine the role of H3K27me3 in regulating TJ genes, we employed EED226, a selective allosteric inhibitor of polycomb repressive complex 2 (PRC2) that directly binds to the H3K27me3 binding pocket of the EED subunit (22), leading to reduced H3K27me3 levels (Figure 3D). Our results demonstrated that inhibition of H3K27me3 mimicked the effect of SLE plasma on TJ genes, reducing their expression at both the mRNA and protein levels (Figure 3, E and F).

Although with different expression patterns, EZH1 and EZH2 are both responsible for catalyzing trimethylation of H3K27 (23). To elucidate why SLE plasma lowers H3K27me3 levels, we measured EZH1 and EZH2 expression at both the mRNA and protein levels. Interestingly, although the mRNA levels of both enzymes stayed the same, SLE plasma treatment reduced EZH2 protein expression in a dose-dependent way (Figure 3G and Supplemental Figure 5, B and C). H3K27me3 levels varied with EZH2 expression: levels were reduced upon EZH2 knockdown and elevated after EZH2 overexpression (Figure 3, H and I, and Supplemental Figure 5, D–G). These results demonstrated that plasma from patients with SLE regulates H3K27me3 levels by modulating EZH2 protein expression.

To connect the glycolytic shift induced by SLE plasma to the downregulation of EZH2 protein, we examined EZH2 levels under conditions where glycolysis and lactate were modulated. As expected, both 2-DG and GSK2837808A reversed the SLE plasma–induced downregulation of EZH2 (Figure 3, J and K, and Supplemental Figure 5, H and I), and exogenous lactate supplementation (sodium lactate or lactic acid) produced effects comparable to those of SLE plasma (Figure 3L). We next directly manipulated EZH2 expression in endothelial cells in the absence of exogenous plasma or lactate stimulation. Knockdown of EZH2 alone was sufficient to downregulate TJ gene expression (Figure 3, M and N, and Supplemental Figure 5J), phenocopying the effect of SLE plasma. Conversely, EZH2 overexpression not only restored the TJ barrier disruption induced by SLE plasma (Figure 3, O and P, and Supplemental Figure 5K) but also rescued the impairment caused by lactic acid supplementation (Figure 3, Q and R). This highlights the key role of EZH2 in regulating HRGEC permeability.

### Lactate induces ubiquitin-dependent degradation of EZH2 in HRGECs.

**Figure 4:**
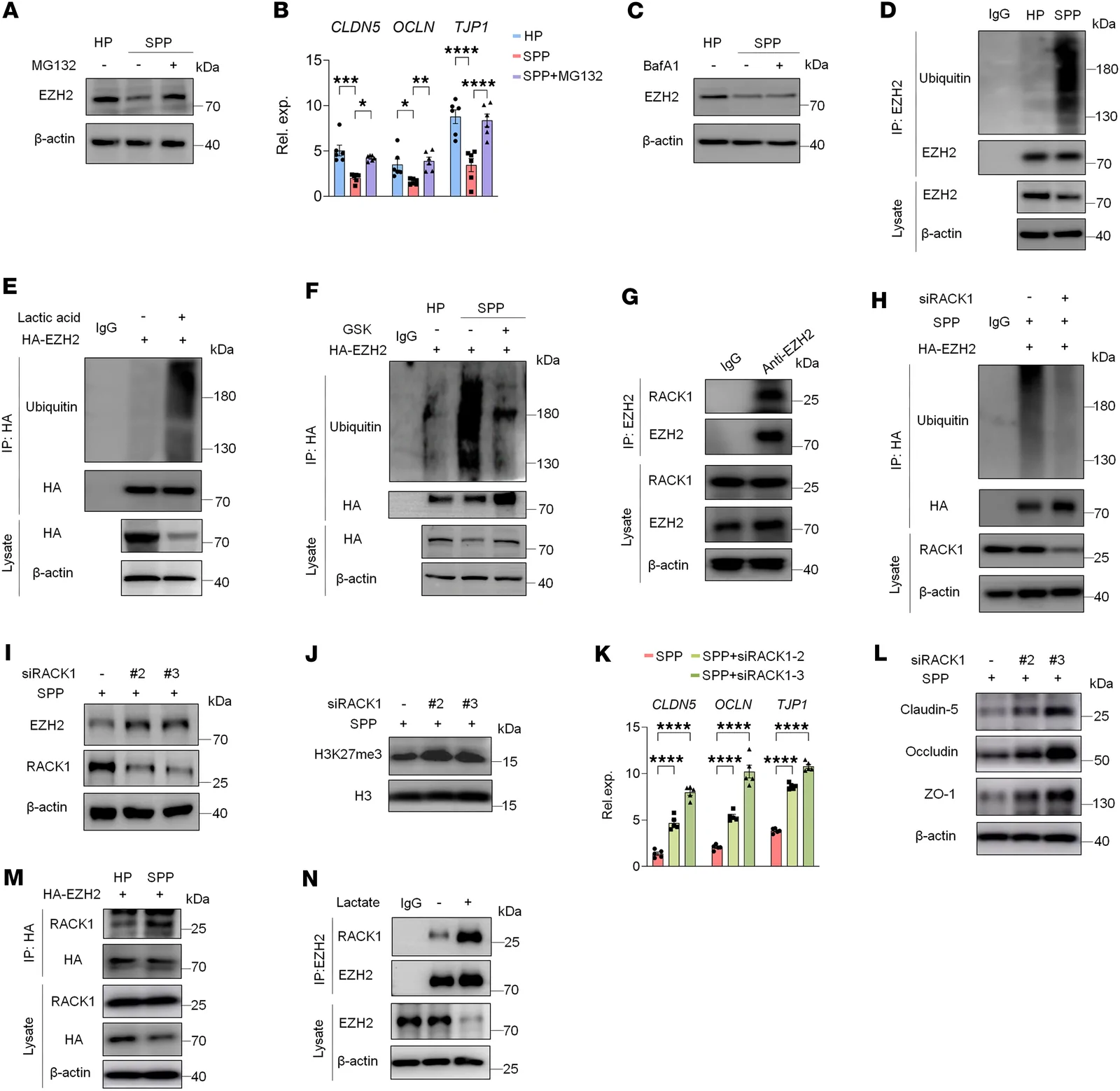
Lactate accumulation induces the ubiquitination of EZH2 in HRGECs. (**A** and **B**) ECs were treated with 10% plasma from HCs or patients with SLE for 24 hours, with or without the addition of MG132 (2 μM) during the final 4 hours of culture. EZH2 protein levels (**A**) and mRNA levels of TJ-related proteins (**B**) were analyzed by Western blot and qPCR, respectively. Mean ± SEM from 6 individuals in each group (**B**). (**C**) ECs were treated with 10% plasma from HCs or patients with SLE for 24 hours, with or without bafilomycin A1 (BafA1, 100 nM, 4 hours). EZH2 protein levels were assessed by Western blot. (**D**) IP analysis of EZH2 ubiquitination was performed in ECs after 24 hours’ treatment with 10% plasma from HC or SLE individuals. (**E**) HA-EZH2 stable ECs were cultured with or without lactic acid (10 mM) for 24 hours, followed by IP analysis of EZH2 ubiquitination. (**F**) IP analysis of EZH2 ubiquitination was performed in HA-EZH2 stable ECs stimulated with 10% plasma from HCs or patients with SLE for 24 hours, with or without 40 μM GSK2837808A cotreatment. (**G**) Co-IP was performed to assess the interaction between EZH2 and RACK1 in HRGECs. (**H**) HA-EZH2 stable ECs were transfected with RACK1-targeting siRNA or control siRNA, then treated with 10% SLE plasma for 24 hours. Subsequent IP analysis evaluated EZH2 ubiquitination in these cells. (**I** and **J**) HRGECs were transfected with RACK1-targeting siRNA or control siRNA, followed by 24 hours’ treatment with 10% SLE plasma. Western blot analysis was used to determine the protein levels of EZH2 (**I**) and H3K27me3 (**J**). H3 was used as the loading control for normalization (**J**). (**K** and **L**) HRGECs were transfected with RACK1-targeting siRNA or control siRNA, followed by 24 hours’ stimulation with 10% SLE plasma. The expression levels of TJ-associated proteins were examined via qPCR (**K**) and Western blot (**L**). Data are mean ± SEM (5 individuals per group for **K**). (**M**) Co-IP assays were conducted in HA-EZH2 stable ECs treated with 10% plasma from HC or SLE individuals to evaluate the interaction between HA-EZH2 and RACK1. (**N**) Co-IP was performed to assess the interaction of EZH2 with RACK1 in HRGECs after culture with or without lactate (40 mM). *P < 0.05, **P < 0.01, ***P < 0.001, and ****P < 0.0001 with 2-way ANOVA plus Tukey’s method (**B** and **K**).

Given that SLE plasma reduced EZH2 protein abundance without altering its mRNA expression, we further explored the underlying protein degradation mechanisms in ECs. Two primary cellular proteolytic systems govern protein turnover: the ubiquitin/proteasome and autophagy/lysosome pathways (17). Pharmacological blockade of the proteasome with MG132, which inhibits the proteolytic activity of the 26S proteasome, fully restored EZH2 expression and reversed the downregulation of TJ transcripts (Figure 4, A and B, and Supplemental Figure 6A). In contrast, lysosomal inhibition with bafilomycin A1 (BafA1), which blocks autophagosome-lysosome fusion, exerted no detectable effect on EZH2 levels (Figure 4C). We next used IP to assess EZH2 ubiquitination and found that SLE plasma treatment markedly increased this posttranslational modification (Figure 4D and Supplemental Figure 6B). Importantly, exogenous lactic acid addition recapitulated this effect, markedly enhancing EZH2 ubiquitination (Figure 4E and Supplemental Figure 6C). In contrast, treatment with GSK2837808A reversed the effect of SLE plasma on EZH2 (Figure 4F). These results establish lactate as the key mediator of EZH2 ubiquitination.

E3 ubiquitin ligases catalyze ubiquitin conjugation onto target substrates (24). To pinpoint the specific E3 responsible for EZH2 ubiquitination, we first screened candidate regulators via the UbiBrowser database, yielding 20 predicted hits (Supplemental Figure 6D), with RACK1 as the top candidate in HRGECs (Supplemental Figure 6E). Subsequent co-IP validated endogenous physical binding between RACK1 and EZH2 (Figure 4G). Subsequently, we used siRNAs to knock down RACK1 expression (Supplemental Figure 6F) and found that RACK1 depletion consistently reduced EZH2 ubiquitination (Figure 4H), thereby preventing EZH2 protein degradation (Figure 4I). This, in turn, restored H3K27me3 levels and alleviated SLE plasma–mediated TJ impairment (Figure 4, J–L). These findings identify RACK1 as the critical E3 ligase for EZH2 in HRGECs. However, neither SLE plasma nor lactate altered RACK1 expression levels (Supplemental Figure 6G). We next examined the binding of EZH2 to RACK1, and our results showed that both SLE plasma and lactate enhanced the binding capacity of EZH2 to RACK1 (Figure 4, M and N), suggesting that lactate may alter the binding between EZH2 and RACK1 in ECs.

### EZH2 lactylation drives its ubiquitination in HRGECs under lactate stress.

**Figure 5:**
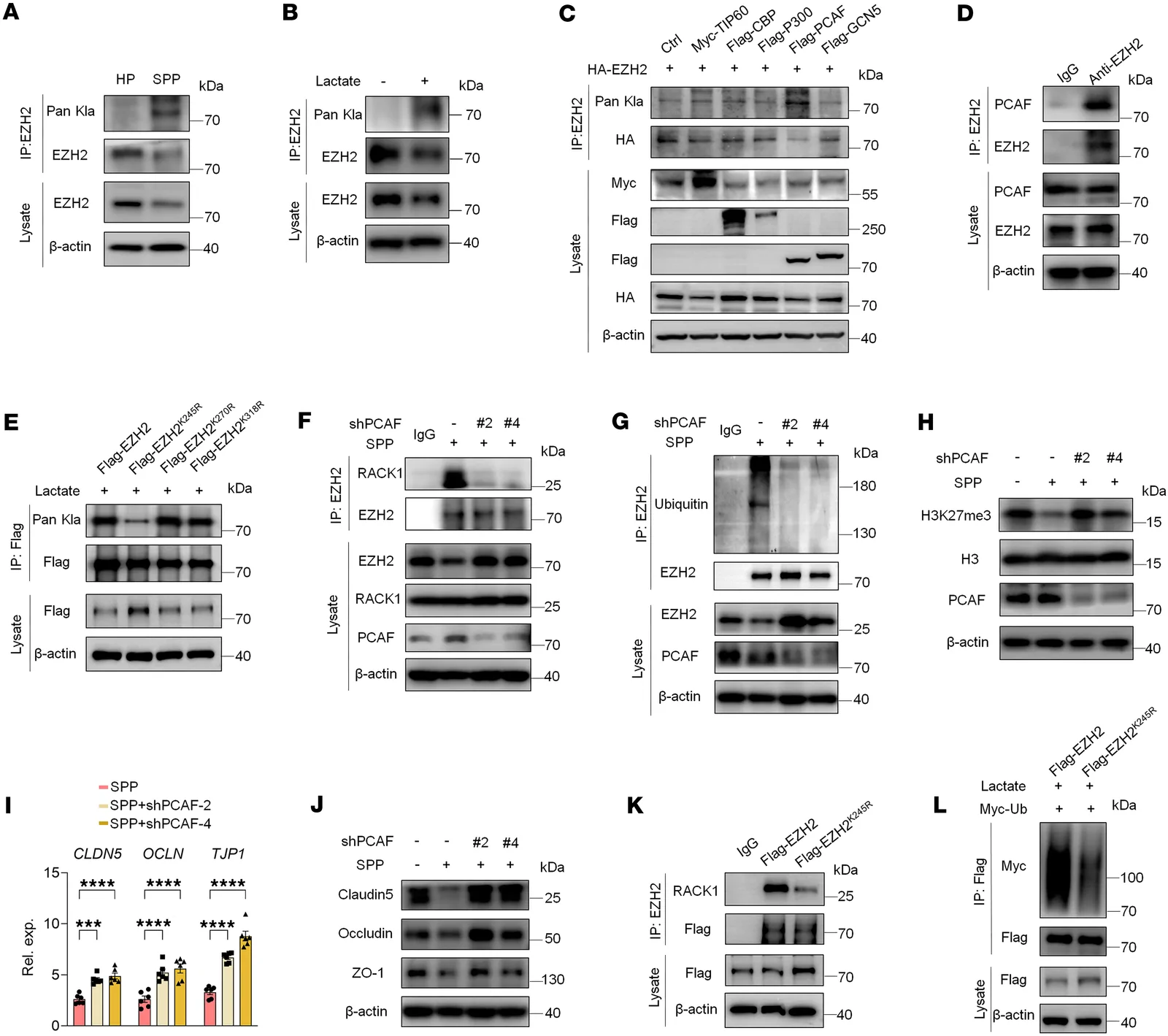
Lactate-induced EZH2 lactylation facilitates its ubiquitination through enhanced RACK1 interaction. (**A**) IP analysis was performed to detect EZH2 lactylation in HRGECs after 24 hours’ treatment with 10% plasma from HC or SLE individuals. (**B**) HRGECs were cultured in the presence or absence of lactate (40 mM) for 24 hours, and EZH2 lactylation was subsequently analyzed via IP. (**C**) IP analysis was conducted to assess EZH2 lactylation in HEK293T cells cotransfected with HA-EZH2 and the indicated acetyltransferase. (**D**) Co-IP analysis was used to examine the interaction between EZH2 and PACF in HRGECs. (**E**) IP analysis was conducted to assess EZH2 lactylation in HEK293T cells transfected with Flag-EZH2 (WT) or the indicated EZH2 mutants. (**F**–**J**) HRGECs were transfected with shRNA targeting PCAF or nontargeting control shRNA, then treated with SLE plasma for 24 hours. (**F**) Co-IP analysis was used to examine the interaction between EZH2 and RACK1. (**G**) IP analysis was conducted to assess EZH2 ubiquitination. (**H**) Western blot was employed to evaluate H3K27me2 levels. qPCR (**I**) and Western blot (**J**) were conducted to assess the expression levels of TJ-related proteins. Mean ± SEM from 6 independent individuals (**I**). (**K**) Co-IP analysis was conducted to assess the interaction between EZH2 and RACK1 in HEK293T cells transfected with Flag-EZH2 (WT) or Flag-EZH2^K245R^ mutants. (**L**) IP was performed to evaluate EZH2 ubiquitination in 40 mM lactate-treated HEK293T cells transfected with Flag-EZH2 (WT) or the Flag-EZH2 (K245R) mutant. ***P < 0.001 and ****P < 0.0001 with 2-way ANOVA plus Tukey’s method (**I**).

Growing evidence suggests that lactate, a ubiquitous metabolic intermediate, mediates a specific posttranslational modification in a wide range of proteins known as lactylation (25). To investigate the mechanism by which lactate accumulation induced by SLE plasma treatment promotes EZH2 ubiquitination, we performed IP of EZH2 to detect its lactylation level. Our results demonstrated that both SLE plasma and lactate markedly increased EZH2 lactylation, a phenomenon accompanied by decreased EZH2 protein levels (Figure 5, A and B, and Supplemental Figure 7A). Canonical histone acetyltransferases are known to dynamically regulate enzyme-dependent lactylation. To identify the specific enzyme responsible for EZH2 lactylation, we systematically overexpressed 5 common acetyltransferases and found that p300/CBP-associated factor (PCAF) selectively enhances EZH2 lactylation (Figure 5C) (26). Co-IP assays further confirmed a direct physical interaction between PCAF and EZH2 in HRGECs (Figure 5D). Using mass spectrometry, we identified K245, K270, and K318 as lactylation sites on EZH2 (Supplemental Figure 7B). Subsequent site-directed mutagenesis revealed that K245 is the primary lactylation residue critical for lactylation of EZH2 (Figure 5E).

To clarify the mechanistic link between lactate-induced EZH2 lactylation and its subsequent ubiquitination, we knocked down PCAF using 2 independent shRNAs (Supplemental Figure 7C). This intervention effectively disrupted the EZH2-RACK1 interaction (Figure 5F), which in turn reduced EZH2 ubiquitination (Figure 5G and Supplemental Figure 7D), restored H3K27me3 levels (Figure 5H), and rescued TJ gene expression (Figure 5, I and J). We next transfected WT and EZH2^K245R^ mutant into HEK293T cells and observed that the EZH2^K245R^ mutant lost the ability to bind RACK1 (Figure 5K), leading to a subsequent reduction in ubiquitination (Figure 5L and Supplemental Figure 7E). Collectively, these findings establish a sequential regulatory axis: PCAF-mediated lactylation at EZH2^K245R^ facilitates RACK1-dependent ubiquitination, which ultimately controls endothelial barrier integrity.

### Sensing of SLE circulating DNA by cGAS initiates endothelial barrier dysfunction.

**Figure 6:**
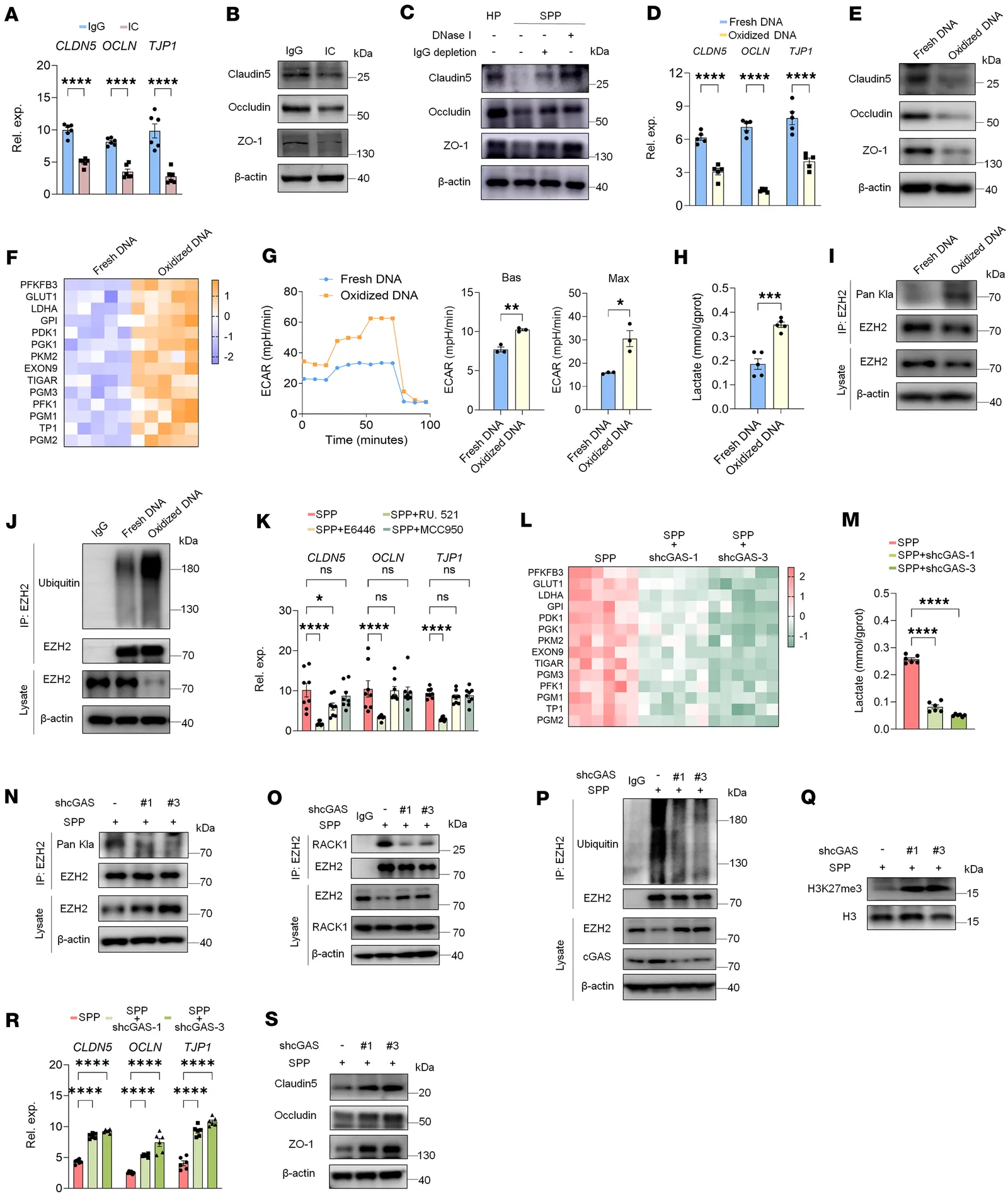
Sensing of oxidized DNA derived from SLE plasma by cGAS serves as the initiator of metabolic perturbation and TJ barrier damage in HRGECs. (**A** and **B**) ECs were incubated with IgG from HCs or SLE-derived ICs (80 IU/mL) for 24 hours. Expression levels of TJ-related proteins were analyzed by qPCR (**A**) and Western blot (**B**). Mean ± SEM (6 individuals/group) (**A**). (**C**) SLE plasma was preincubated with or without protein A/G (1:100 protein A/G/plasma) or DNase I (1:10 DNase I/plasma) before treating HRGECs; HC plasma received no pretreatment. TJ-associated protein expression was detected by Western blot. (**D**–**J**) ECs were cultured with fresh DNA or circulating oxidized DNA derived from SLE patient plasma for 24 hours. TJ-related protein expression was detected by qPCR (**D**) and Western blot (**E**). Data are mean ± SEM (5 individuals/group, **D**). (**F**) Glycolysis-related gene expression was examined by qPCR. (**G**) ECAR was measured using a Seahorse assay kit. Mean ± SEM; 3 individuals per group. (**H**) Lactate concentrations were quantified using an assay kit. Mean ± SEM; 5 individuals per group. (**I**) IP analysis was conducted to assess EZH2 lactylation. (**J**) IP analysis was conducted to detect EZH2 ubiquitination. (**K**) HRGECs were pretreated with RU320521 (RU.521, 10 μM), E6446 (100 nM), or MCC950 (100 nM) for 24 hours, followed by 24 hours’ treatment with 10% SLE plasma. mRNA levels of TJ-related proteins were measured by qPCR. Mean ± SEM (8 individuals/group). (**L**–**S**) HRGECs were transfected with cGAS-targeting shRNAs or nontargeting control shRNAs, then cultured with SLE plasma. (**L**) Glycolysis-related gene expression was examined by qPCR. (**M**) Lactate concentrations were quantified using an assay kit. Mean ± SEM (6 individuals/group). (**N**) IP analysis was conducted to assess EZH2 lactylation. (**O**) Co-IP analysis was performed to examine the interaction between EZH2 and RACK1. (**P**) IP analysis was used to detect EZH2 ubiquitination. (**Q**) H3K27me3 levels were evaluated by Western blot. H3 was used as the loading control for normalization. Expression of TJ-related proteins analyzed by qPCR (**R**) and Western blot (**S**). Mean ± SEM (6 individuals/group) (**R**). *P < 0.05, **P < 0.01, ***P < 0.001, and ****P < 0.0001 with unpaired 2-tailed *t* test (**G** and **H**) and 1-way (**A**) and 2-way ANOVA plus Tukey’s method (**D**, **K**, **M**, and **R**).

In circulation, self-DNA released from apoptotic cells is highly oxidized and plays a crucial role in SLE pathogenesis: it triggers the production of IgG anti-dsDNA antibodies, leading to the formation of pathogenic ICs and subsequent organ damage (15). Our prior work has demonstrated that such DNA-containing ICs represent the critical bioactive component in SLE patient plasma, directly driving pathological mesangial cell proliferation (2). To clarify whether circulating ICs mediate TJ disruption in LN, we treated ECs with ICs isolated from SLE plasma and analyzed the expression of key TJ components. Our results showed that IC pretreatment substantially downregulated the expression of TJ-related genes, confirming that ICs play a critical role in inducing endothelial barrier dysfunction (Figure 6, A and B). Intriguingly, enzymatic removal of the DNA moiety with DNase I completely abrogated this inhibitory effect (Figure 6C), whereas depletion of the IgG backbone only partially rescued TJ expression (Figure 6C), pointing to the nucleic acid cargo as the predominant active factor. These findings align with the coexistence of IgG-complexed DNA and cell-free circulating DNA in peripheral blood of patients with SLE. To further verify the independent regulatory effect of SLE plasma–derived circulating DNA, exogenous DNA was supplemented into EC culture medium separately. Notably, oxidized DNA rather than native fresh DNA recapitulated the inhibitory effect on TJ-related gene transcription in these cells (Figure 6, D and E, and Supplemental Figure 8, A and B). These results indicate that the SLE circulating DNA is the critical component mediating SLE plasma–induced endothelial barrier dysfunction. In addition, exogenous oxidized DNA follows the same pathway as SLE plasma, inducing a metabolic shift in HRGECs toward glycolysis (Figure 6, F and G) and increasing lactate production (Figure 6H). This accumulated lactate then promotes EZH2 lactylation (Figure 6I), which in turn facilitates EZH2 ubiquitination and degradation (Figure 6J).

DNA sensing is associated with cellular metabolic reprogramming (27). cGAS, NLRP3 inflammasome, and TLR9 are the most classic receptors that mediate DNA immune responses (14). To identify the key DNA sensor responsible for SLE-DNA–triggered TJ breakdown in HRGECs, we pharmacologically blocked canonical DNA sensors with selective small-molecule inhibitors: RU320521 (RU.521) for cGAS, MCC950 for NLRP3 inflammasome, and E6446 for TLR9 (16). Notably, our results indicate that only inhibition of cGAS effectively blocked the oxidized DNA-induced TJ impairment in HRGECs (Figure 6K). Genetic depletion of cGAS (Supplemental Figure 8C) effectively attenuated oxidized DNA-induced glycolytic activation (Figure 6L). This metabolic normalization subsequently reduced lactate accumulation (Figure 6M), which in turn diminished EZH2 lactylation (Figure 6N). Decreased lactylation impaired the interaction between EZH2 and RACK1 (Figure 6O), leading to reduced EZH2 ubiquitination (Figure 6P). Consequently, stabilized EZH2 protein levels restored H3K27me3 deposition (Figure 6Q) and ultimately rescued TJ barrier integrity (Figure 6, R and S, and Supplemental Figure 8D). These results demonstrate that cGAS-dependent DNA-sensing orchestrates endothelial barrier dysfunction through the sequential EZH2 lactylation/ubiquitination axis.

### Targeting cGAS-mediated sensing of self-DNA and inhibiting lactic acid accumulation suppress LN progression in SLE models.

**Figure 7:**
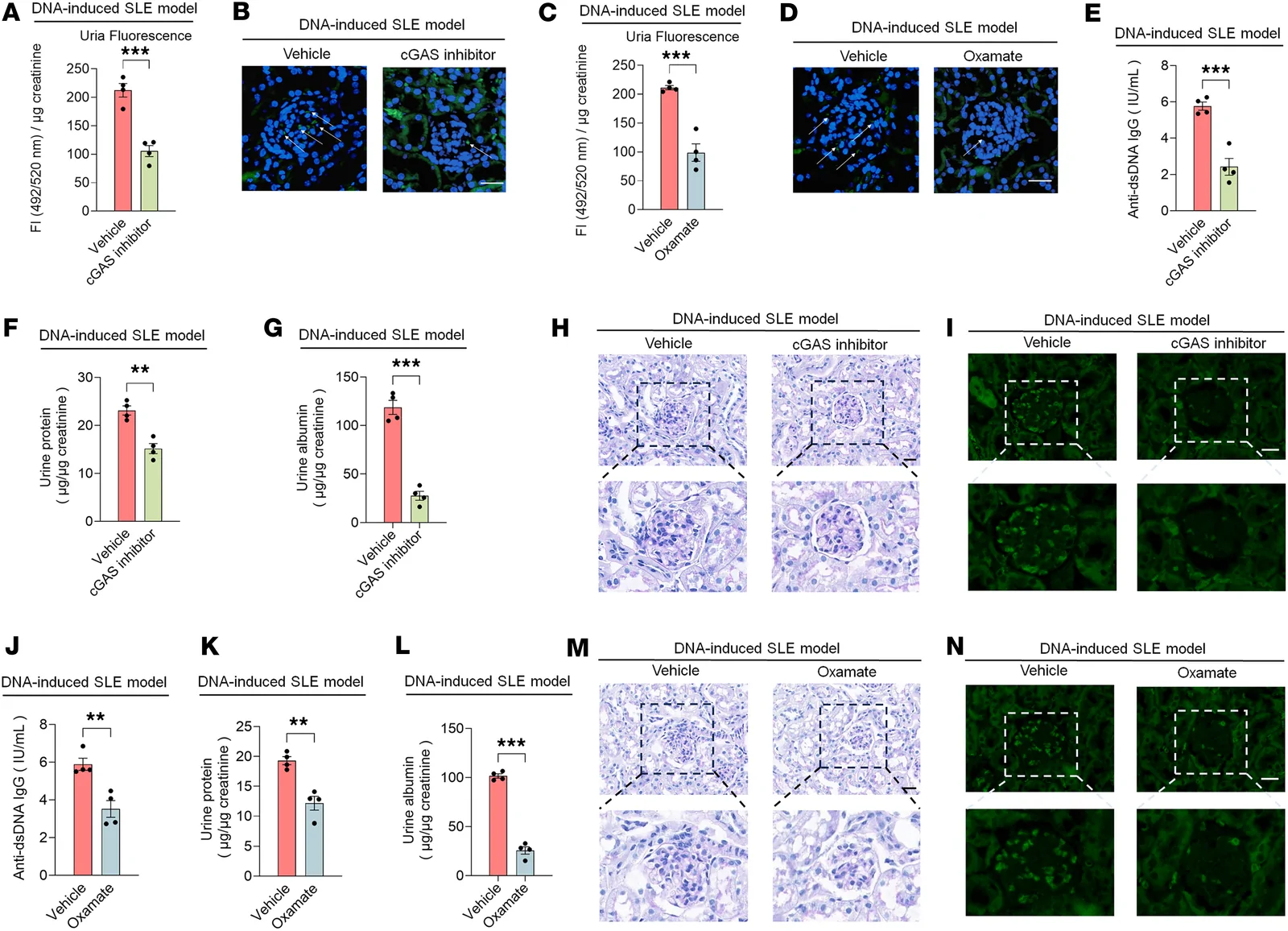
RU.521 and oxamate exert a therapeutic effect by restoring TJ barrier damage and ameliorating LN-related symptoms. (**A**) Urinary 4 kDa FITC-dextran fluorescence intensity normalized to creatinine was measured via microplate reader in SLE model mice treated with or without RU.521. Data shown as mean ± SEM (*n* = 4 per group). (**B**) Glomerular localization of 70 kDa FITC-conjugated dextran in SLE model mice (with or without RU.521 treatment) was visualized by immunofluorescence microscopy. Scale bar: 50 μm. (**C**) Urinary 4 kDa FITC-dextran fluorescence intensity normalized to creatinine was measured via microplate reader in SLE model mice treated with or without oxamate. Data shown as mean ± SEM (*n* = 4 per group). (**D**) Immunofluorescence microscopy was used to visualize the glomerular localization of 70 kDa FITC-conjugated dextran in SLE model mice (with or without oxamate treatment). Scale bar: 50 μm. (**E**–**I**) SLE model mice were treated with or without RU.521. (**E**) Plasma levels of anti-dsDNA IgG were measured using an ELISA kit. (**F**) Proteinuria normalized to creatinine was quantified via Bradford protein assay. (**G**) Albuminuria normalized to creatinine was quantified via BCG albumin assay kit. (**H**) Glomerular pathological changes were visualized by periodic acid–Schiff (PAS) staining. Scale bar: 20 μm. (**I**) Glomerular localization of IgG was detected by immunofluorescence. Scale bar: 20 μm. Mean ± SEM from 4 mice per group (**E**–**G**). (**J**–**N**) SLE model mice were administered with or without oxamate. (**J**) Plasma concentrations of anti-dsDNA IgG were determined using an ELISA kit. (**K**) Proteinuria normalized to creatinine was quantified via Bradford protein assay. (**L**) Albuminuria normalized to creatinine was quantified via BCG albumin assay kit. (**M**) Pathological alterations in glomeruli were observed via PAS staining. Scale bar: 20 μm. (**N**) Localization of IgG in glomeruli was visualized through immunofluorescence. Scale bar: 20 μm. Mean ± SEM from 4 mice per group (**J**–**L**). **P < 0.01, ***P < 0.001, and ****P < 0.0001 with unpaired 2-tailed *t* test (**A**, **C**, **E**–**G**, and **J**–**L**).

To evaluate the therapeutic effect of targeting both cGAS activation and lactate accumulation in vivo, we implemented a targeted intervention strategy in a self-DNA–induced SLE mouse model. Starting 1 week after disease induction, mice were administered either the cGAS inhibitor RU.521 to specifically block DNA-sensing pathways or the LDHA inhibitor sodium oxamate to selectively suppress lactate production. After cGAS inhibitor RU.521 or oxamate pretreatment, levels of 4 kDa FITC-dextran in urine and 70 kDa FITC-dextran in glomeruli were substantially reduced (Figure 7, A–D), both indicators of improved endothelial permeability. Additionally, LN symptoms in mice were alleviated, as evidenced by decreased serum anti-dsDNA antibodies, reduced proteinuria and albuminuria, restored glomerular morphology, and attenuated renal IgG deposition (Figure 7, E–N). These findings provide direct in vivo evidence that circulating oxidized DNA-induced lactic acid accumulation contributes to endothelial barrier dysfunction in LN.

To verify the generalizability of our findings in a classic spontaneous SLE model, we performed additional in vivo experiments using MRL/lpr mice. Pharmacological inhibition of either cGAS or LDHA (via oxamate) markedly ameliorated LN-related pathological phenotypes in MRL/lpr mice, including attenuated serum anti-dsDNA antibodies and proteinuria, improved renal histology, and reduced glomerular IgG deposition (Supplemental Figure 9, A–D).

## Discussion

TJs, composed of transmembrane proteins (occludin, claudins) and cytoplasmic scaffold proteins (ZO-1/2/3) (28), form a structural complex that seals the epithelium and enables paracellular transport through 2 distinct routes: the pore pathway (claudin-mediated, selective for small cations such as Na^+^) and the leak pathway (occludin-regulated, which permits molecules >0.6 nm, including lactulose, mannitol, and 4 kDa dextran) (29–31). Among these, claudin5 is a critical determinant of endothelial barrier function (32), and ZO-1 plays a dominant role in TJ assembly over ZO-2/3 (33, 34). TJ dysfunction in response to inflammatory signaling is strongly associated with endothelial barrier disruption and constitutes a common pathogenic mechanism across multiple diseases, such as systemic vascular leakage (observed in sepsis) (35), blood-brain barrier breakdown (associated with stroke) (36), impaired CNS vascular integrity (causing multiple sclerosis) (37), and proteinuria (accompanied by diabetic kidney disease) (38). Increased permeability to 4 kDa dextran indicates TJ damage (39), and 70 kDa dextran deposition in the aortic wall serves as a marker of compromised TJ integrity (39). Previous studies have linked endothelial layer integrity to the development of neurological complications in SLE. When human brain microvascular ECs are exposed to lupus serum, TNF-like weak inducer of apoptosis (TWEAK), and C5a, increased permeability — monitored via changes in transendothelial electrical resistance — occurs, accompanied by downregulated expression of claudin5 and ZO-1 (40). However, whether HRGEC TJ integrity contributes to LN pathogenesis remains unclear. In our study, we assessed TJ function by examining claudin5, occludin, and ZO-1 because these molecules are key regulators of TJ-mediated paracellular permeability. Consistent with TJ disruption, we observed increased urinary fluorescence intensity of 4 kDa dextran and glomerular accumulation of 70 kDa dextran in DNA-induced lupus mice, demonstrating impaired HRGEC barrier function in LN. Unexpectedly, inhibition of cGAS or LDHA reduced glomerular accumulation of 70 kDa dextran and concurrently increased its reabsorption in the proximal tubules. Although the underlying mechanism remains to be fully elucidated here, multiple studies have associated cGAS signaling and lactate metabolism with tubular epithelial dysfunction (41, 42). Accumulating evidence also demonstrates well-established glomerulotubular crosstalk, whereby primary glomerular injury initiates secondary tubular lesions (43).

In our study, we found that EZH2-dependent H3K27me3 is essential for promoting the transcription of TJ-related genes in HRGECs. These findings underscore cell type–specific regulatory mechanisms governing TJ gene expression. Meanwhile, SLE plasma induces widespread alterations in histone methylation, including H3K4me2, H3K9me2, H3K36me, H3K79me3, and H4K20me2. However, the functional implications of these specific modifications, which may not be directly associated with TJ impairment, remain unexplored. EZH2-mediated H3K27me3 has been reported to regulate TJ genes in ECs (44), but its effect varies by context: in a depression model, EZH2 recruited to the Cldn5 promoter represses claudin5 (45); in proximal tubular cells, injury-induced EZH2 upregulation suppresses ZO-1 (46). To resolve this discrepancy, we compared HRGECs and HK2 cells and found that HK2 cells exhibited higher basal EZH2 and H3K27me3 levels and opposing regulatory patterns (Supplemental Figure 10, A–C). These differences align with the principles that basal H3K27me3 abundance varies across cell types, dictating its positive or negative regulatory outcome, and that the gene pools subjected to such regulation are lineage dependent (47). Based on this principle, ZO-1 is plausibly regulated positively by H3K27me3 in glomerular ECs, yet suppressed by excessive H3K27me3 in injured tubular cells, explaining the opposite ZO-1 responses to EZH2 silencing between our work and the published report. The complexity of H3K27me3 regulation is further underscored by a study showing that JMJD3-mediated demethylation of H3K27me3, rather than excessive methylation, is the primary driver of blood–spinal cord barrier disruption and TJ impairment (48).

In SLE, immune cells, including DCs, macrophages, T cells, and B cells, display metabolic reprogramming that leads to lactic acid accumulation. This metabolic shift is closely associated with enhanced antigen presentation, proinflammatory responses, and antibody production (49, 50). Our recent work showed that enhanced glycolysis drives lactate accumulation not only in immune cells but also in resident kidney cells such as mesangial cells, promoting mesangial proliferation and glomerular injury (2). In diabetic kidney disease, IGF-binding protein 5 (IGFBP5) upregulation increases glycolysis in glomerular ECs (51), but this pathway is not operative in our system, as IGFBP5 blockade failed to rescue SPP-induced glycolysis (Supplemental Figure 10D). Abnormal glycolysis-derived lactic acid is widely recognized as a key driver of protein lactylation (52, 53). Prior evidence has also validated that early growth response protein 1 (EGR1) lactylation upregulates heparinase expression, thereby disrupting the endothelial glycocalyx and contributing to endothelial dysfunction (54). Notably, despite consistent endothelial glycocalyx damage in LN, no alteration in EGR1 lactylation was detected in HRGECs after stimulation with plasma from patients with SLE (Supplemental Figure 10E). In this work, we found that lactic acid accumulation acts as a trigger for EZH2 degradation. Administration of oxamate, which inhibits lactic acid production (2), ameliorated TJ injury in HRGECs and alleviated LN symptoms in SLE mice. Meanwhile, we acknowledge that the lack of cell-type specificity makes it difficult to attribute these effects solely to HRGECs due to potential off-target effects on other cell types.

EZH2 lactylation and subsequent ubiquitination constitute a key mechanism linking glycolysis-driven EZH2 degradation to TJ impairment in HRGECs. Lactylation, initially identified on histone lysine residues, can coexist with acetylation at distinct sites (55). The process is initiated by conversion of Warburg effect–derived lactate to lactyl-coenzyme A via GTPSCS (56), which serves as the direct donor for acetyltransferase-mediated lactylation (57), illustrating the broader principle of posttranslational modification crosstalk (58). Specifically, lactylation of EZH2 at K245 enhances its association with the E3 ubiquitin ligase adaptor RACK1, thereby promoting EZH2 degradation. This supports a model wherein substrate accumulation induces allosteric remodeling that exposes a RACK1-binding domain, ultimately governing EZH2 stability (59).

The breakdown of nucleic acid tolerance and consequent activation of the IFN system are central to SLE pathogenesis (60). A key pathogenic loop involves the constant accumulation of immunogenic self-DNA, exacerbated by impaired clearance mechanisms such as reduced DNase activity. This is exemplified by a lupus model induced via immunization with immunogenic self-DNA from apoptotic cells, which underscores the pivotal role of self-DNA in driving the disease (61, 62). In general, circulating self-DNA acts as a damage-associated molecular pattern: it is taken up by APCs and then sensed by various intracellular DNA sensors, including cGAS, TLR9, NLRP3, AIM2, and ENPP1 (16, 63). These sensors in turn promote the activation of the adaptive immune response (16) and the generation of anti-dsDNA autoantibodies (15) — a hallmark of SLE that correlates with disease activity (64). In LN, these autoantibodies and self-DNA form deposits, either as ICs or independently, on resident kidney cells such as mesangial cells. This pathological deposition directly disrupts the GFB, culminating in proteinuria (2). Our prior work confirmed that DNA sensing drives LN pathogenesis via mesangial proliferation induced by cell-cycle regulator degradation (2). Given that HRGECs can directly interact with circulating self-DNA and possess inherent endocytic activity (65), investigating DNA-induced functional defects in HRGECs is biologically reasonable. Upon internalization, DNA can be sensed by cGAS/STING, which typically activates IRF3 and NF-κB signaling pathways; these pathways have been shown to promote the expression of key glycolytic enzymes (66) — an observation consistent with our present findings. Numerous studies have validated the essential role of cGAS/STING activation in SLE pathogenesis (50, 67, 68). Our previous study also revealed that DNA-triggered cGAS/STING signaling in HRGECs facilitates the differentiation of CD4^+^ tissue-resident memory T cells and accelerates LN progression (69). A seemingly contradictory report by Motwani et al. showed that systemic germline knockout of cGAS or STING in MRL/lpr mice and in the 2,6,10,14‑tetramethylpentadecane–induced (TMPD‑induced) lupus model paradoxically exacerbated disease (70). However, this conclusion remains relatively niche. In contrast, the majority of recent studies using independent genetic interventions have demonstrated that cGAS/STING deficiency alleviates lupus-like pathology. For instance, in the imiquinod-induced lupus model, cGAS or STING knockout markedly alleviated lupus symptoms (71, 72). Notably, in the TMPD-induced model, a STING loss-of-function mutation reduced disease severity (73). These observations indicate that the exacerbation reported by Motwani et al. is likely model- or intervention-dependent and does not represent a generalizable role of cGAS/STING in SLE. Admittedly, we acknowledge one limitation of this study: we did not perform plasma proteomics to rule out the effects of other plasma components, and our analysis focused exclusively on DNA.

In summary, our work uncovers a pathway linking circulating self-DNA to GFB dysfunction in LN. We establish that DNA-induced abnormal glycolysis initiates a cascade of EZH2 lactylation and ubiquitination, leading to epigenetic silencing of TJ genes. This mechanistic insight suggests a druggable metabolic/epigenetic axis that may offer therapeutic opportunities beyond standard immunosuppression.

## Methods

### Sex as a biological variable.

Given the marked female predominance of SLE (14), this study enrolled predominantly women (97/108 patients with SLE and 57/64 HCs) for experiments involving human samples. Accordingly, female mouse strains (BALB/c, MRL/lpr, and C57BL/6) were used for animal experiments.

### Patients and healthy donors.

A total of 108 patients diagnosed with SLE were enrolled in this study. Individuals with other comorbid conditions were excluded. Sixty-four age- and sex-matched healthy donors, with no history of malignancy or other autoimmune disorders, were recruited as controls. Clinical characteristics of the participants are summarized in Supplemental Table 1. All human samples were randomly allocated to experimental groups without bias.

### Cell culture.

HRGECs were purchased from Shanghai Binsui Biotechnology Co., Ltd. HUVECs and HEK293T cells were obtained from ATCC. All cell lines were cultured in DMEM (Gibco) supplemented with 10% FBS (Sigma-Aldrich) at 37°C with 5% CO_2_. Mouse splenocytes were cultured in RPMI-1640 medium (Gibco) containing 10% FBS (PAN-Biotech).

### Mice and DNA-induced SLE model.

BALB/c mice were purchased from GemPharmatech. The LN model was induced using self-DNA as previously described (13). Briefly, genomic self-DNA was extracted from splenocytes of 6-week-old female BALB/c mice that had been stimulated in vitro with concanavalin A (5 μg/mL, 6 days) to induce apoptosis. An additional cohort of BALB/c mice was divided into 4 groups. To establish a lupus-like model, 3 groups were subcutaneously immunized with 0.2 mL of an emulsion containing self-DNA in PBS combined with complete Freund’s adjuvant (CFA) at week 0. Control mice received PBS emulsified with CFA alone. Booster immunizations were performed at weeks 2 and 4 using self-DNA emulsified with incomplete Freund’s adjuvant. After the third immunization, 2 groups from the lupus model cohort received daily treatments for 14 days with either RU.521 (5 mg/kg) or oxamate (600 mg/kg). Six weeks after treatment, analyses were conducted for serum mouse IgG anti-dsDNA antibodies, renal IgG deposition, renal histology, and urine protein levels.

AAV2 vectors conjugated to the glomerular endothelium homing peptide QVLVYRE (designated AAV2GEC) were constructed as reported previously (19). To selectively silence ZO1 in glomerular ECs, a ZO1-targeted shRNA was packaged into AAV2GEC to generate AAV2GECshZO1; an AAV2GEC vector carrying a scrambled nontargeting shRNA was produced as a negative control. On day 14 after modeling, mice were administered AAV2GECshZO1 or AAV2GECshNC via the tail vein. All animals were euthanized 4 weeks after administration, followed by assessments of renal IgG deposition, renal histological lesions, and urinary protein levels.

MRL/lpr mice were obtained from SPF Biotechnology Co. (D206), and 12-week-old female MRL/lpr mice were treated with RU.521 (5 mg/kg) or oxamate (600 mg/kg) twice weekly until 20 weeks of age. Age-matched female C57BL/6 mice served as normal controls. At the experimental endpoint, whole blood, urine, and kidneys were collected. Serum anti-dsDNA IgG antibodies, urinary protein levels, renal IgG deposition, and renal histology were then analyzed.

### Transfections and reagents.

ECs were transfected with siRNA targeting human RACK1, EZH2, or LDHA, along with nontargeting control siRNA (all purchased from GeneAdv), using Lipofectamine Stem (Thermo Fisher Scientific, STEM00001).

For plasmid transfections in HEK293T cells, expression constructs for EZH2, TIP60, CBP, P300, PCAF, GCN5, as well as shRNAs targeting EZH2, RACK1, LDHA, PCAF, and cGAS (synthesized by Miaoling Biology), were introduced via transient transfection by polyethyleneimine (50).

Lentiviral particles for gene overexpression or knockdown in ECs were generated in HEK293T cells through cotransfection with the transfer plasmid and packaging plasmids pVSVg and psPAX2. The viral supernatant was collected 48 hours after transfection, filtered through a 0.45 μm polyethersulfone membrane (MilliporeSigma, SLHP033RB), and used to transduce ECs at a 1:1 ratio with fresh culture medium supplemented with polybrene (8 μg/mL) (MedChemExpress) (16). Stable ECs overexpressing HA-tagged EZH2 were generated by transfection with the HA-EZH2 construct, followed by puromycin selection.

Reagents were obtained from the following sources: MG132, BafA1, 2-DG, GSK2837808A, EED226, E6446, MCC950, and RU.521 were purchased from MedChemExpress; sodium l-lactate was sourced from Sigma-Aldrich; and NBI-31772 was purchased from TargetMol. The following assay kits were used: pyruvate assay kit (Yuanye Bio-Technology, R22024), lactic acid assay kit (Abbkine, KTB1100), acetyl-CoA assay kit (Elabscience, E-EL-0125), Bradford protein assay kit (GeneRay Biotech, GK5021), Amplex red creatinine assay kit (Beyotime, S0291S), albumin assay kit with BCG (Beyotime, P0383S), and anti-dsDNA IgG ELISA kit (Abnova, KA1100). All reagents and kits were used in accordance with the manufacturers’ instructions.

### Real-time PCR.

Total RNA was isolated from samples using TRIzol (Takara) following the protocol described in previous publications (74). cDNA was synthesized using Hifair III First Strand cDNA Synthesis SuperMix (Yeason). qPCR was conducted with SYBR Green qPCR Master Mix (Selleckchem), with 18S ribosomal RNA serving as the endogenous control. Relative mRNA expression levels were calculated using the 2^−ΔΔCt^ method. Primer sequences are listed in Supplemental Table 2.

### Immunoblotting and IP.

Immunoblotting and IP were carried out as described previously (75). Briefly, cells were lysed on ice for 30 minutes using RIPA buffer (50 mM Tris-HCl, pH 7.5; 150 mM NaCl; 10% glycerol; 0.5%–1% Triton X-100, 100 μM PMSF). The lysates were centrifuged at 14,000*g* for 15 minutes at 4°C, and the resulting supernatants were collected for further analysis. For immunoblotting, proteins were separated by SDS-PAGE and transferred to membranes. For IP, cleared lysates were incubated overnight at 4°C with specific antibodies coupled to protein A/G beads. The immunoprecipitated complexes were washed and subjected to immunoblot analysis with indicated antibodies. Antibodies used were as follows: anti-ZO-1 (Cell Signaling Technology, 13663), anti-occludin (Cell Signaling Technology, 91131), anti-claudin5 (Cell Signaling Technology, 49564), anti-PLVAP (Epizyme, P103129), anti-LDHA (Santa Cruz Biotechnology, sc-137243), anti-H3 (Abcam, ab1791), anti-H3K4me2 (Abclonal, A22143), anti-H3K9me2 (Abclonal, A26196), anti-H3K27me3 (Abclonal, A22006), anti-H3K36me (Abclonal, A20379), anti-H3K79me3 (Abclonal, A2369), anti-H4 (Abclonal, A23000), anti-H4K20me2 (Abclonal, A2371), anti-EZH1 (Abclonal, A5818), anti-EZH2 (Cell Signaling Technology, 5246; Proteintech, 21800-1-AP), anti-RACK1 (Proteintech, 27592-1-AP), anti-l-lactyl lysine (PTM, PTM-1401RM), anti–β-actin (Proteintech, 66009-1-Ig), anti-Flag (Proteintech, 66008-4-Ig), anti-HA (Bioss, bsm-33157M), anti-Myc (Abmart, M20002M), anti-PCAF (Proteintech, 28770-1-AP), anti-ubiquitin (Proteintech, 10201-2-AP), anti-cGAS (Santa Cruz Biotechnology, sc-515777), and anti-EGR1 (Proteintech, 22008-1-AP).

### Flow cytometry.

Glucose uptake was assessed in HRGECs using the fluorescent glucose analog 2-NBDG (Invitrogen, N13195). Briefly, cells were serum-starved for 2 hours, followed by incubation with 2-NBDG for 30 minutes at 37°C under 5% CO_2_. After washing, fluorescence was measured on a Canto II flow cytometer (BD Biosciences), and data were analyzed with FlowJo software.

To assess glomerular endothelial claudin5, occludin, and ZO-1 expression in vivo, kidneys were harvested from euthanized mice, and glomeruli were isolated following a differential adhesion protocol (76). In brief, after decapsulation and medulla removal, the cortical tissue was minced and digested in collagenase type V (1 mg/mL in HBSS, C8170, Solarbio) at 37°C for 15 minutes. The reaction was stopped by adding an equal volume of DMEM supplemented with 10% FBS. The resulting suspension was then serially strained through 100 μm, 70 μm, and 40 μm cell strainers. The glomerular-enriched fraction retained on the 40 μm mesh was collected, placed into a 10 cm dish, and left to settle for 1–2 minutes, allowing tubular fragments to adhere to the plastic surface. The supernatant, now enriched with glomeruli, was carefully recovered; this adhesion step was repeated twice to maximize purity. After centrifugation at 290*g* for 5 minutes, the glomerular pellet was resuspended in PBS. The isolated glomeruli were next dissociated into single cells, fixed and permeabilized using fixation and permeabilization solution (BD Biosciences, 554722), and incubated overnight at 4°C with primary antibodies against CD31 (Abclonal, A27114) combined with either anti-claudin5 (Proteintech, 29767-1-AP), anti-occludin (Proteintech, 27260-1-AP), or anti-ZO-1 (Proteintech, 21773-1-AP). After washing, the cells were stained with appropriate Alexa Fluor–conjugated secondary antibodies and analyzed by flow cytometry. The MFI of each TJ protein was compared specifically within the CD31^+^ EC population.

### Immunofluorescence.

For detection of glomerular IgG deposition, paraffin-embedded kidney sections were deparaffinized, subjected to antigen retrieval, and blocked according to established protocols. Sections were then incubated with an anti-mouse IgG (H+L) F(ab’)_2_ Fragment (Alexa Fluor 488 Conjugate; Cell Signaling Technology, 4408S).

For TJ staining in HRGECs, cells grown in 8-well chamber slides were fixed and immunolabeled with antibodies against ZO-1 (Cell Signaling Technology, 13663), occludin (Cell Signaling Technology, 91131), and claudin5 (Cell Signaling Technology, 49564), followed by incubation with DyLight 649-conjugated (Abbkine, A23610) and DyLight 488-conjugated (Abbkine, A23220) secondary antibodies. Nuclei were counterstained with Hoechst (Yeasen, 40731ES10). Images were acquired using a Nikon confocal microscope and processed with ImageJ (NIH).

### Transmission electron microscopy.

After euthanasia, mouse kidneys were rapidly isolated, and the renal cortex was dissected into approximately 1 mm^3^ tissue blocks. The harvested tissues were immediately fixed in ice-cold 2.5% electron microscopy–grade glutaraldehyde for 2 hours at room temperature. After thorough fixation, the samples were rinsed 3 times with 0.1 M phosphate buffer (pH 7.4), postfixed in 1% osmium tetroxide at 4°C for 1–2 hours, and sequentially dehydrated using a graded ethanol series (30%, 50%, 70%, 80%, 90%, 95%, and 100%) followed by pure acetone. Subsequently, the specimens were infiltrated and embedded in epoxy resin. Ultrathin sections of 60–80 nm thickness were prepared with an ultramicrotome, double-stained with uranyl acetate and lead citrate, and observed and imaged under a Hitachi HT-7700 transmission electron microscope.

### ECAR.

Glycolytic function was assessed by measuring the ECAR using an XF24 Extracellular Flux Analyzer (Seahorse Biosciences) and the Agilent Seahorse XF Glycolytic Stress Test kit. HRGECs were seeded in XF24 plates at 2 × 10^4^ cells per well and allowed to adhere overnight. Cells were then treated for 24 hours with either 10% plasma isolated from patients or self-DNA derived from apoptotic cells. All assay procedures were performed according to the manufacturer’s protocol (50).

### Mass spectrum.

For EZH2 lactylation analysis, HEK293T cells were overexpressed with Flag-EZH2 for 48 hours and treated with or without 40 mM sodium l-lactate in the last 24 hours before lysis. Cell lysates were immunoprecipitated using anti-Flag magnetic beads (Selleckchem, B26102). For the histone methylation assay, HRGECs were incubated with plasma from healthy donors or patients with SLE, and histone H3 and H4 were immunoprecipitated with specific antibodies. All immunoprecipitates were separated by SDS-PAGE and stained with Coomassie brilliant blue. Target protein bands were excised and subjected to in-gel tryptic digestion, and the purified peptides were analyzed by mass spectrometry (Thermo Fisher Scientific).

### RNA-seq.

Total RNA (1–2 μg per sample) was used to construct sequencing libraries with the KAPA Stranded RNA-Seq kit (Illumina). Raw reads were adapter-trimmed and quality-filtered (Q20 threshold) to generate clean data. HISAT2 (default parameters) aligned reads to the human reference genome, and HTSeq quantified gene expression. Differential expression was assessed with DESeq2, considering genes with fold-change greater than 2 and adjusted *P* less than 0.05 as significant.

### Kidney single-cell data analysis.

The single-cell RNA-seq (scRNA-seq) data from 24 patients with biopsy-proven LN were obtained from ImmPort database (accession SDY997) and from the NCBI’s Sequence Read Archive (accession PRJNA379992). After quality control, 2,882 high-quality single cells expressing 23,372 genes were retained for analysis. Cell clustering was performed using Seurat (v4.2.0). An initial low-resolution clustering identified major renal cell types, including HRGECs, podocytes, and mesangial cells. To investigate endothelial barrier function, we examined the expression of key cell adhesion genes (e.g., CLDN5, OCLN, TJP1, and CDH5). Finally, Pearson’s correlation analysis was conducted between the aggregate expression of these adhesion-related genes and the SELENA-SLEDAI total score for each corresponding patient.

### IC isolation and identification.

DNA-ICs from SLE patient plasma and IgG from healthy donor plasma were purified using the PurKine Antibody Purification kit (Protein A/G) (Abbkine, KTP2070) according to the manufacturer’s protocol (2).

### FITC-dextran permeability assay.

Endothelial barrier integrity was evaluated using a Transwell system (0.4 μm and 8 μm pore size). HRGECs were cultured on inserts until full confluence was achieved. After treatment with patient-derived plasma or lactate, FITC dextran (4 kDa/70kDa) was applied to the apical chamber. After 1 hour of incubation at 37°C, the basolateral medium was collected and fluorescence was quantified (excitation/emission: 490/520 nm). Permeability was expressed as relative fluorescence units.

To assess renal permeability in mice, FITC-labeled dextran (MW 4 kDa and 70 kDa; Beyotime, ST2930) was administered via tail vein injection (250 mg/kg). After cardiac perfusion to wash out intravascular free dye, kidney sections were subsequently prepared and examined by fluorescence imaging.

### Statistics.

Data are presented as mean ± SEM. Differences between 2 groups were assessed using a 2-tailed Student’s *t* test; multiple comparisons were analyzed by 1-way ANOVA followed by Tukey’s post hoc test. All analyses were conducted with GraphPad Prism 10.2.3. Statistical significance was defined as *P* less than 0.05.

### Study approval.

This study was approved by the Ethics Committee of Soochow University. All participant-related procedures were conducted in accordance with the Declaration of Helsinki, and written informed consent was obtained from all participants prior to enrollment. All animal experiments involving NSG mice were performed in compliance with the ARRIVE guidelines.

### Data availability.

The mass spectrometry data have been deposited in the OMIX database of the China National Center for Bioinformation/Beijing Institute of Genomics, Chinese Academy of Sciences (https://ngdc.cncb.ac.cn/omix; accession OMIX018013 and OMIX018016). The RNA-seq data have been deposited in the Science Data Bank (https://www.scidb.cn/en/list; DOI: https://doi.org/10.57760/sciencedb.41069). All raw numerical data are provided in the Supporting Data Values file. Additional relevant data can be obtained from the corresponding author upon reasonable request.

## Author contributions

ZW, LX, TL, and ZL designed the study. JL, XZ, YW, YL, Lei Li, ML, JG, Lingyi Li, and ZC performed experiments and analyzed data. LX, TL, and QC participated in data interpretation. JL and ZW wrote the manuscript with the input of all authors.

## Conflict of interest

The authors have declared that no conflict of interest exists.

## Funding support

- National Natural Science Foundation of China (82271841 to ZW).
- National Natural Science Foundation of China (824B2047 to JL).
- Jiangsu Provincial Health Innovation Team (to ZW).
- Priority Academic Program Development of Jiangsu Higher Education Institutions.
- Basic Research Program of Jiangsu (BK20255001 to ZW).
- China Postdoctoral Science Foundation (2025M781398 and 2026T190395 to ML).
- Wuxi Municipal Commission of Health (M202320 to TL).
- Wuxi Municipal Bureau of Science and Technology (Y20232009 to FY).

## Supplementary Material

Supplemental data

Unedited blot and gel images

Supporting data values

## Version 1 08/18/2026

In-Press Preview

## Version 2 09/22/2026

Electronic publication
