# Supplementary material for: Lactate controls glomerular endothelial barrier integrity in lupus nephritis
Source: JCI Insight. 2026 Aug 18;11(18):e205034. doi: 10.1172/jci.insight.205034 (PMC13596713; doi:10.1172/jci.insight.205034)
Supplement: Supplementary file 1 [file jciinsight-11-205034-s074.pdf]

Supplementary Figure 1

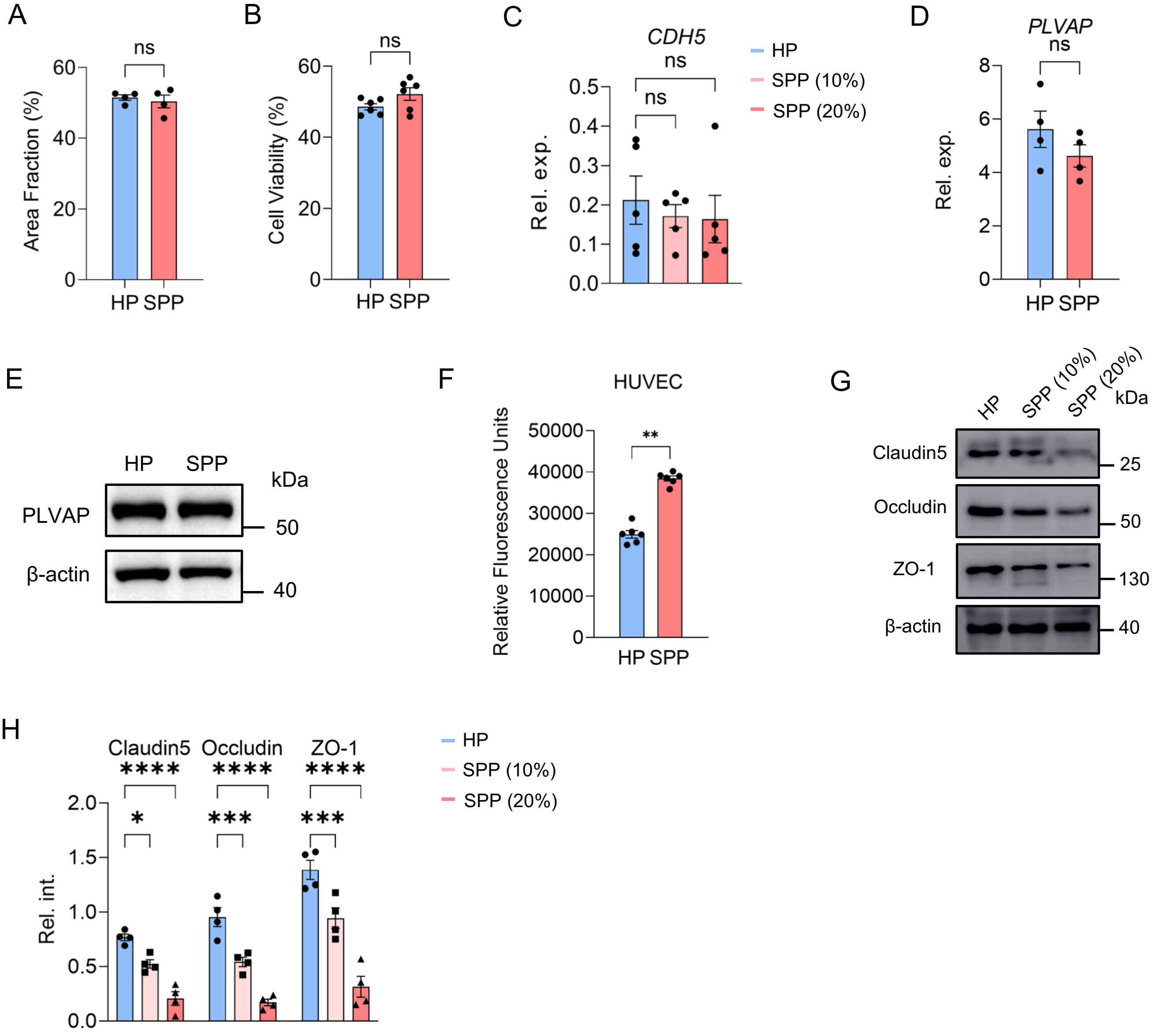

Fig. S1. SLE plasma impairs the TJ barrier

(A-B) HRGECs were incubated with 10% plasma from HCs or SLE patients for 24 h. Cell confluency was analyzed via ImageJ (A), and cell viability was quantified using the CCK-8 assay (B). Data are mean ± SEM, n = 4-6 individuals per group. (C) Endothelial cells were cultured with 10% or 20% plasma from HCs or SLE patients. qPCR was performed to measure mRNA levels of VE-cadherin. Data are mean ± SEM, n = 5 individuals per group. (D-E) HRGECs were treated with 10% plasma from HCs or SLE patients for 24 h. PLVAP expression was assessed by qPCR (D) and western blot (E). Data are mean ± SEM, n = 4 individuals per group (D). (F) HUVECs were exposed to 10% plasma from HCs or SLE patients for 24 h. Fluorescence intensity of 4 kDa FITC-dextran in the lower transwell chamber was quantified with a microplate reader. Data are mean ± SEM, n = 6 individuals per group. (G-H) HUVECs were incubated with 10% or 20% plasma from HCs or SLE patients. Western blot was used to detect the abundance of TJ-associated proteins. Data are mean ± SEM, n = 4 individuals per group. *$p < 0.05$, **$p < 0.01$, ***$p < 0.001$, and ****$p < 0.0001$ with unpaired t test (F) and ANOVA plus Tukey's method (H).

Supplementary Figure 2

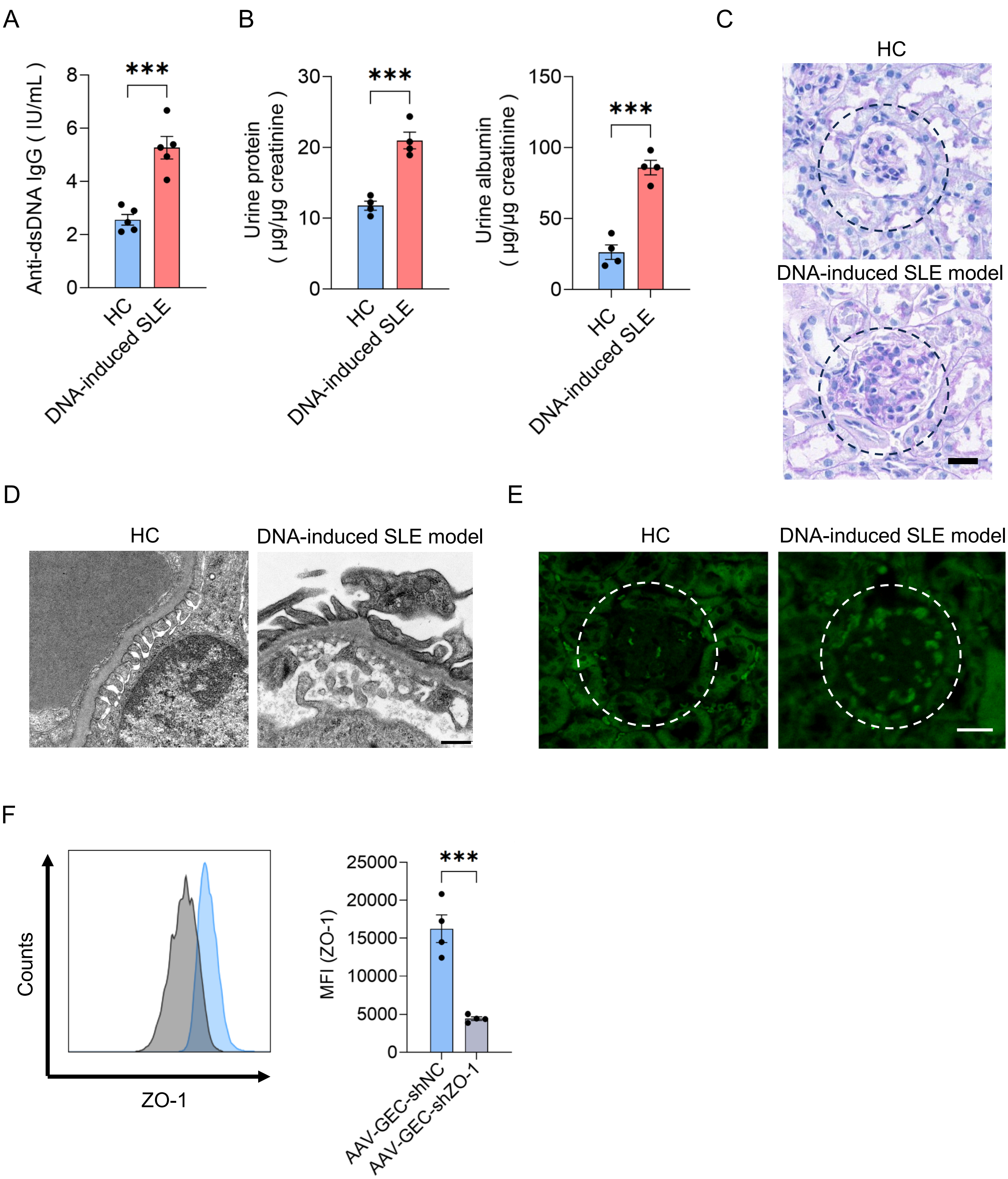

Fig. S2. Establishment of DNA-induced lupus mouse model

(A) Plasma anti-dsDNA IgG levels in healthy and SLE model mice were measured by ELISA. Data are mean ± SEM, n = 5 mice per group. (B) Urinary proteinuria and albuminuria normalized to creatinine were quantified using the Bradford assay and BCG albumin assay kit, respectively. Data are mean ± SEM, n = 4 mice per group. (C) Glomerular pathological lesions were assessed by Periodic acid–Schiff (PAS) staining. Scale bar, 20 μm. (D) Representative transmission electron micrographs of the glomerular filtration barrier. Scale bar, 500 nm. (E) Glomerular IgG deposition was visualized by immunofluorescence staining. Scale bar, 20 μm. (F) Healthy mice were subjected to treatment with AAV2-GEC-shZO-1 or negative control shRNA. ZO-1 knockdown efficiency was determined via flow cytometry. Data are mean ± SEM, n = 4 mice per group. ****p* < 0.001 with unpaired t-test (A-B, F).

Supplementary Figure 3

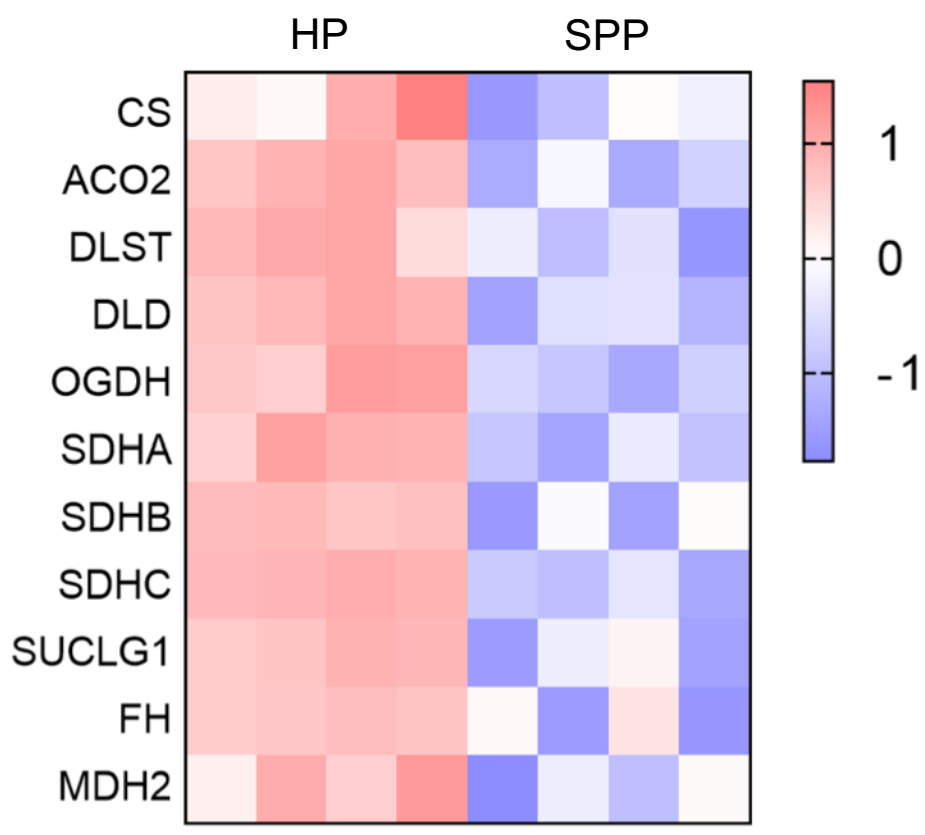

Fig. S3. Expression of TCA cycle-related genes in endothelial cells

qPCR analysis of TCA cycle-related genes in endothelial cells treated for 24 hours with plasma from HCs or SLE patients.

Supplementary Figure 4

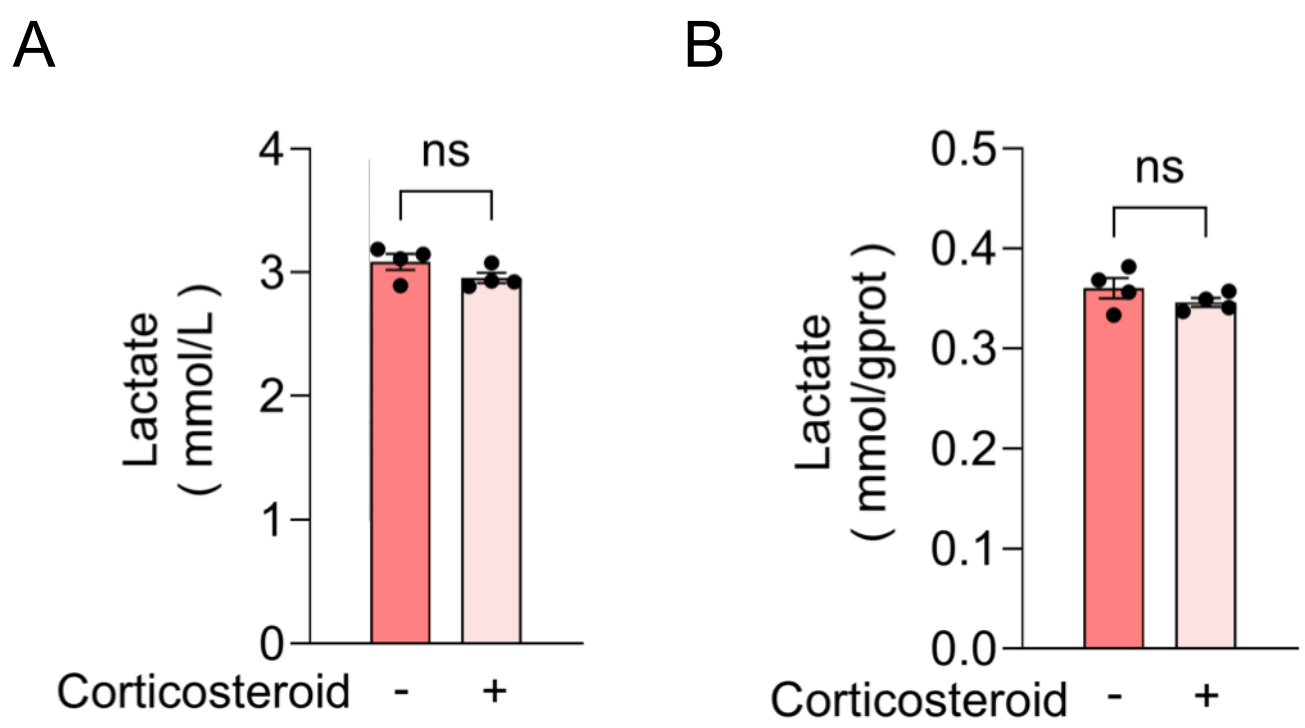

Fig. S4. Effects of corticosteroid on lactate production in HRGECs stimulated with SLE patient plasma

(A) Lactate concentrations in plasma collected from SLE patients pre- and post-corticosteroid treatment were quantified using a commercial assay kit. Data are mean ± SEM, n = 4 individuals per group. (B) Intracellular lactate levels were measured in HRGECs incubated with 10% plasma from SLE patients with or without corticosteroid intervention. Data are mean ± SEM, n = 4 individuals per group.

Supplementary Figure 5

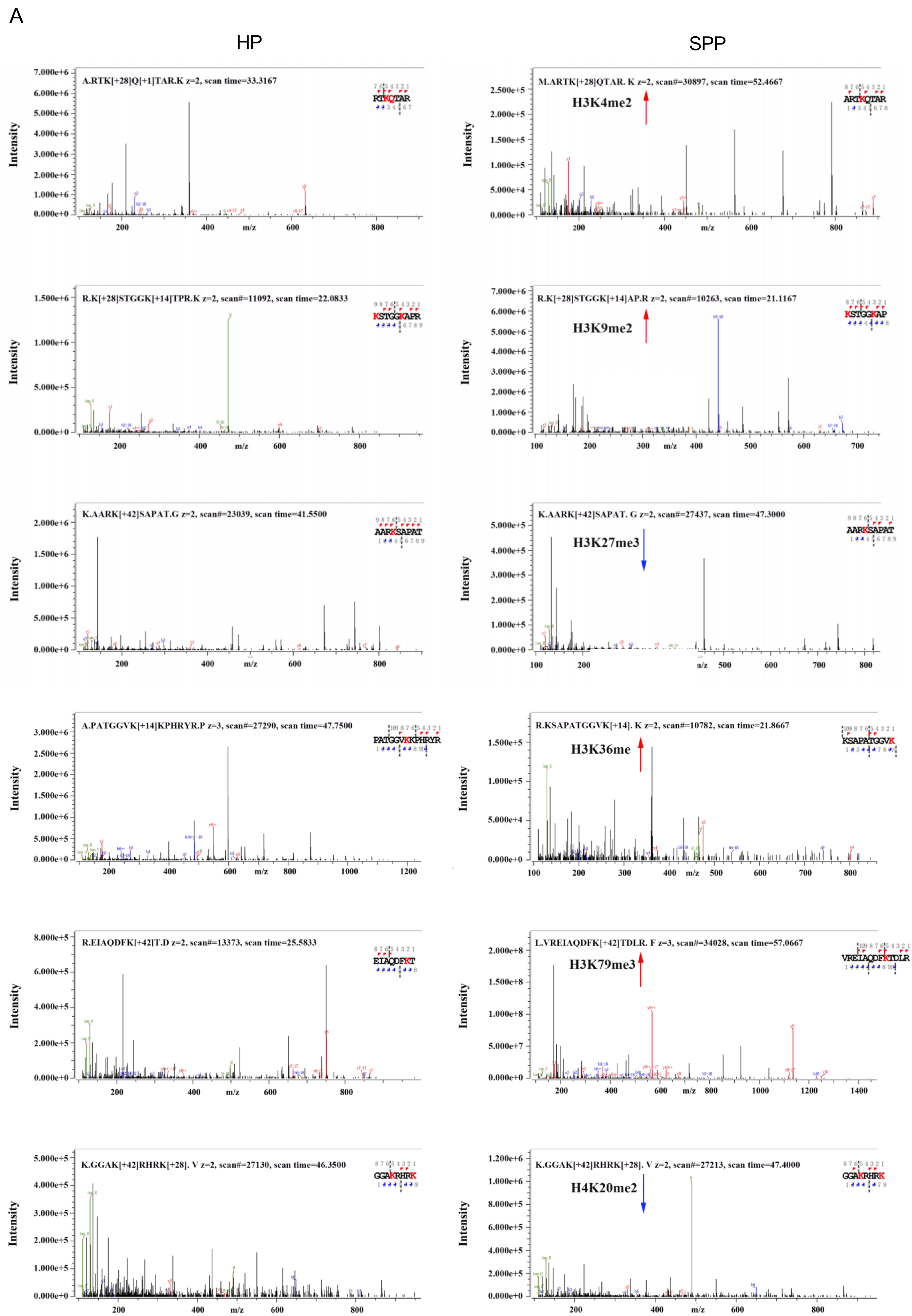

Supplementary Figure 5

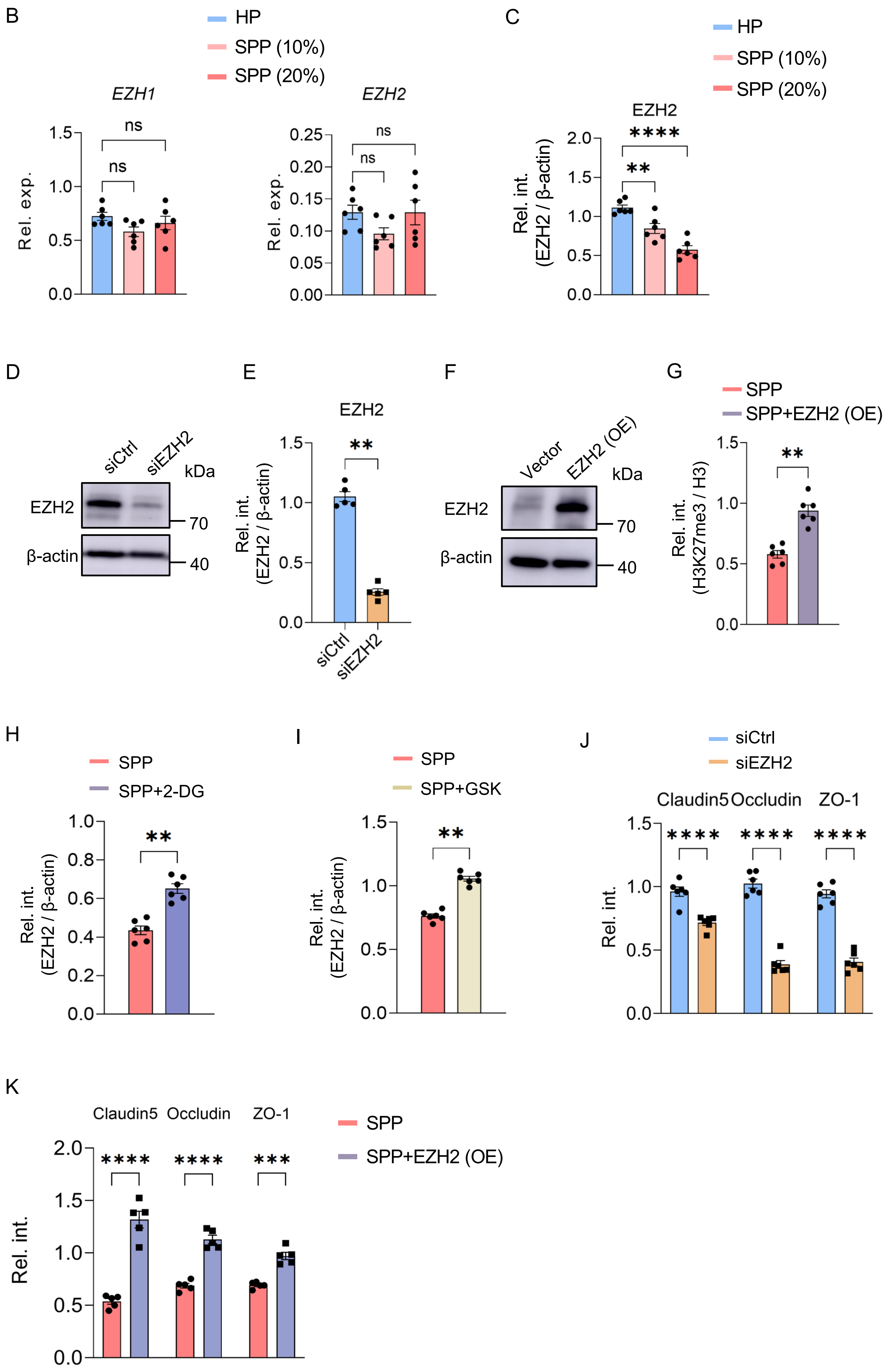

Fig. S5. The regulation of EZH2 on TJ-related proteins

(A) HRGECs were treated with 10% plasma from HCs or SLE patients for 24 h and subjected to mass spectrometry analysis. (B-C) Endothelial cells were incubated with 10% or 20% plasma from HCs or SLE patients for 24 h. EZH1 and EZH2 mRNA levels were assessed by qPCR, and EZH2 protein expression was quantified by ImageJ. Data are mean ± SEM, n = 6 individuals per group. (D-E) Endothelial cells were transfected with EZH2-targeting siRNA. EZH2 protein expression was examined by western blot and quantified by ImageJ. Data are mean ± SEM, n = 5 independent experiments per group. (F-G) Endothelial cells were transfected with an EZH2-overexpressing plasmid prior to treatment with 10% plasma derived from SLE patients. EZH2 and H3K27me3 protein abundances were detected via western blot and quantified using ImageJ. Data are mean ± SEM, n = 6 independent experiments per group (G). (H-I) Endothelial cells were stimulated with 10% SLE plasma for 24 h with or without 2-DG (1 mM) (H) or GSK2837808A (40 μM) (I). EZH2 protein levels were quantified by ImageJ. Data are mean ± SEM, n = 6 individuals per group. (J) TJ-associated protein levels were quantified by ImageJ in endothelial cells after EZH2 knockdown. Data are mean ± SEM, n = 6 independent experiments per group. (K) Levels of TJ-associated proteins in endothelial cells stimulated with 10% SLE patient-derived plasma for 24 h, with or without EZH2 overexpression, were quantified using ImageJ. Data are mean ± SEM, n = 5 individuals per group. ** $p < 0.01$, *** $p < 0.001$, and **** $p < 0.0001$ with paired t-test (E, G-I) and ANOVA plus Tukey's method (C, J-K).

Supplementary Figure 6

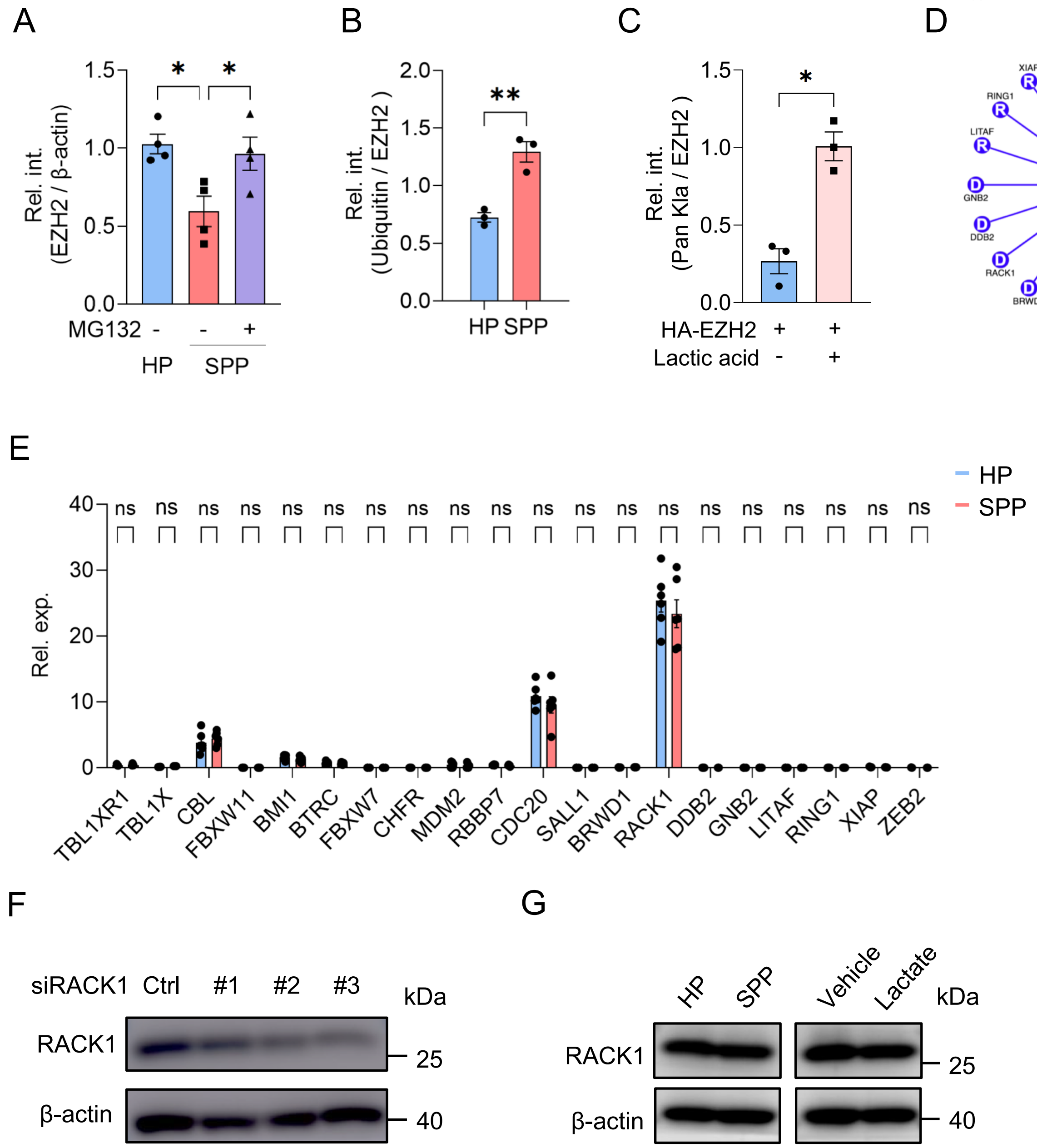

Fig. S6. RACK1 as a potential E3 ligase for EZH2

(A) Endothelial cells were treated with 10% plasma from HC or SLE patients for 24 h, with or without MG132 (2 μM) added during the final 4 h. EZH2 protein levels were quantified by ImageJ. Mean ± SEM from 4 individuals in each group. (B) Endothelial cells were incubated with 10% HC or SLE plasma for 24 h, and EZH2 ubiquitination levels were quantified by ImageJ. Mean ± SEM from 3 individuals in each group. (C) HA-EZH2 stable endothelial cells were cultured with or without lactic acid (10 mM) for 24 h, and EZH2 ubiquitination levels were quantified by ImageJ. Mean ± SEM from 3 independent experiments in each group. (D) Potential E3 ligase of EZH2 obtained from the UbiBrowser database. (E) mRNA levels of candidate E3 ligases in HRGECs treated with plasma from different groups were measured by qPCR. Mean ± SEM from 6 individuals in each group. (F) Western blot was performed to assess RACK1 protein levels in HEK293T cells transfected with three specific siRNAs targeting RACK1. (G) RACK1 expression in response to plasma or lactate treatment (40 mM) was evaluated by western blot. *$p < 0.05$ and **$p < 0.01$ with paired t test (C), unpaired t test (B) and ANOVA plus Tukey's method (A).

Supplementary Figure 7

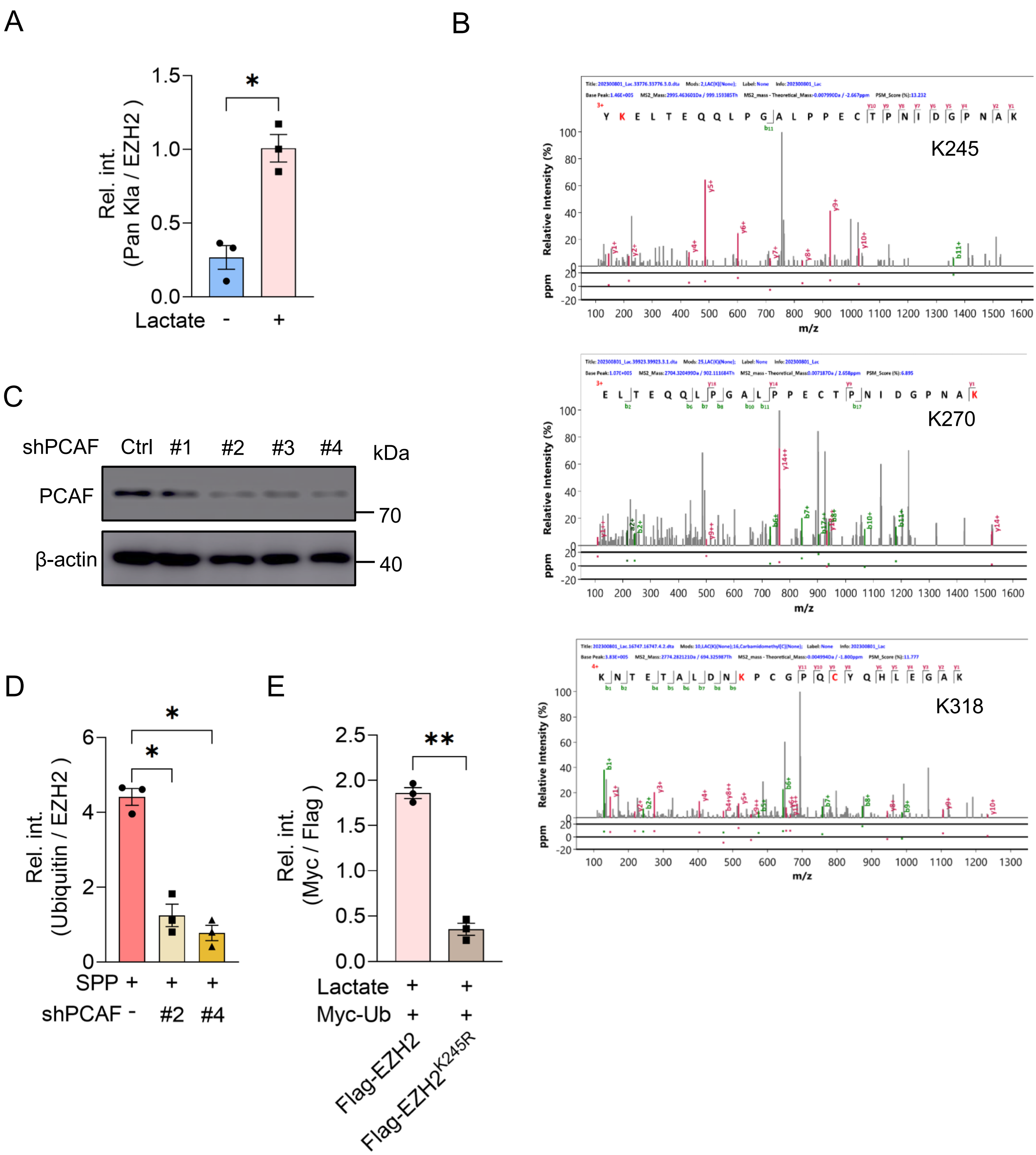

Fig. S7. Characterization of the acetyltransferase and lactylation sites of EZH2

(A) HRGECs were cultured with or without lactate (40 mM) for 24 h. EZH2 lactylation was quantified by ImageJ. Mean ± SEM from 3 independent experiments in each group. (B) HEK293T cells transfected with HA-EZH2 were treated with lactate (40 mM) for 24 h, then subjected to mass spectrometry analysis. (C) Western blot analysis of PCAF protein levels in HEK293T cells transfected with four specific shRNAs targeting PCAF. (D) HRGECs transfected with PCAF shRNA or control shRNA were stimulated with SLE plasma for 24 h. EZH2 ubiquitination was quantified by ImageJ. Mean ± SEM from 3 individuals in each group. (E) HEK293T cells expressing Flag-EZH2 (WT or K245R) were lactate-treated (40 mM), followed by anti-Flag immunoprecipitation. EZH2 ubiquitination was examined by immunoblotting and quantified via ImageJ. Mean ± SEM from 3 independent experiments in each group. *$p < 0.05$ and **$p < 0.01$ with paired t test (A, E) and ANOVA plus Tukey's method (D).

Supplementary Figure 8

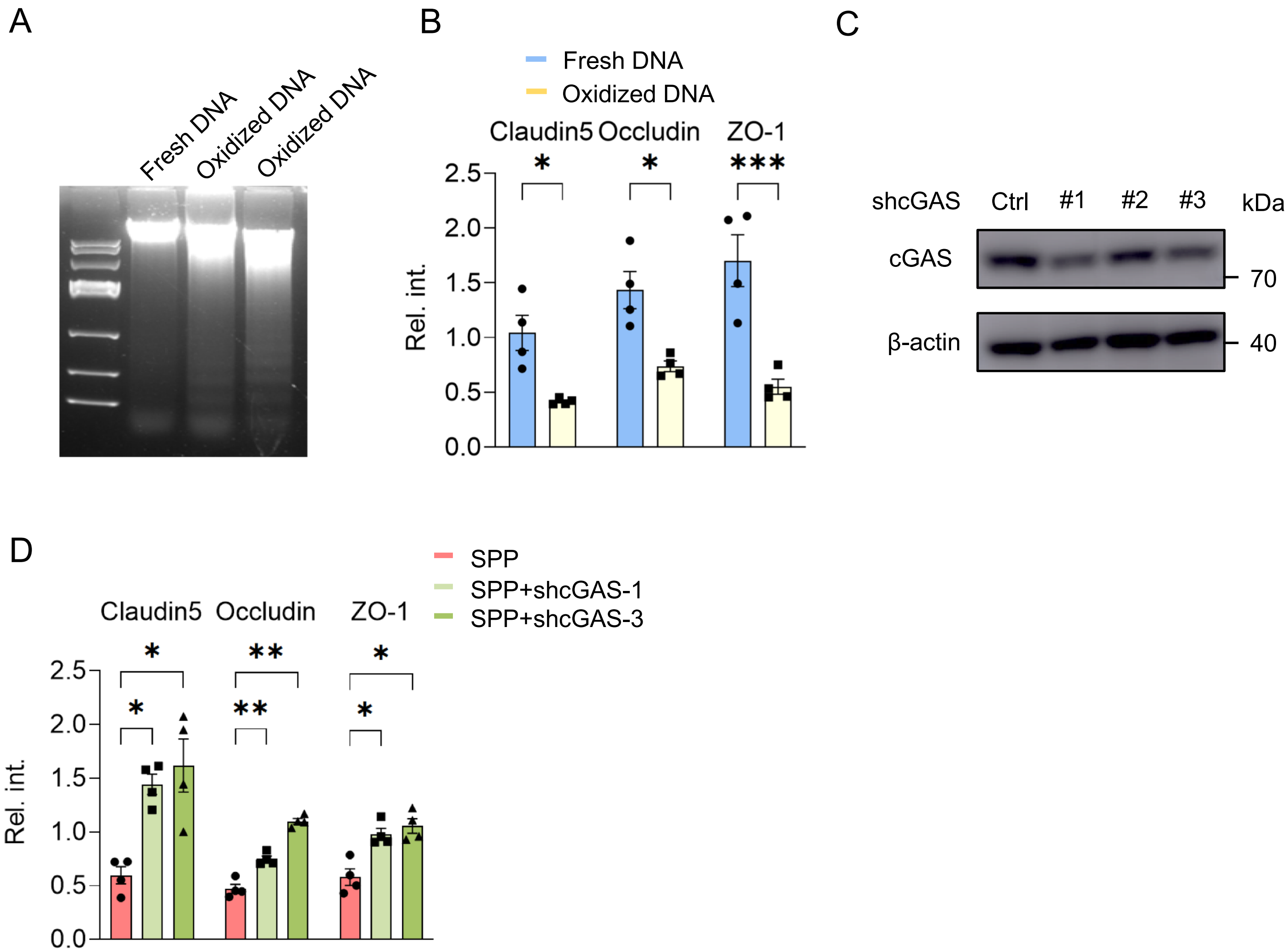

Fig. S8. cGAS acts as a sensor for oxidized DNA in endothelial cells

(A) Identification of extracted fresh DNA (from untreated endothelial cells) or circulating oxidized DNA (from the plasma of SLE patients). (B) Endothelial cells were treated with fresh DNA or SLE-derived oxidized DNA for 24 h. TJ-related protein expression was quantified by ImageJ. Mean ± SEM from 4 individuals in each group. (C) Western blot was performed to assess cGAS protein levels in HEK293T cells transfected with three specific shRNAs targeting cGAS. (D) HRGECs transfected with cGAS-targeting shRNA or non-targeting control shRNA were incubated with SLE plasma for 24 h. TJ-related protein expression was quantified by ImageJ. Mean ± SEM from 4 individuals in each group. *$p < 0.05$, **$p < 0.01$ and ***$p < 0.001$ with ANOVA plus Tukey's method (B, D).

Supplementary Figure 9

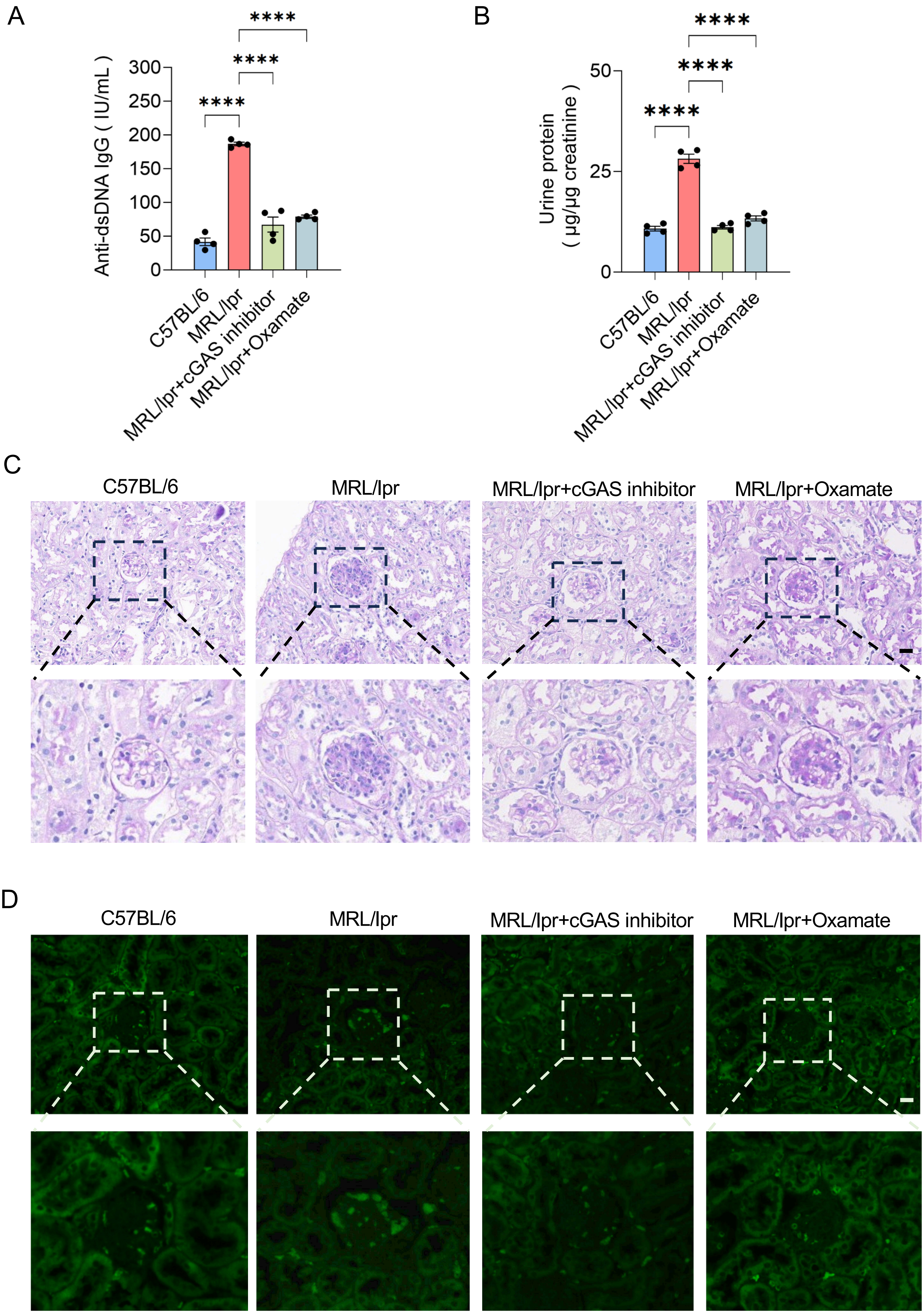

Fig. S9 RU.521 and oxamate ameliorate LN-related pathologies in MRL/lpr mice

(A-D) MRL/lpr mice were treated with RU.521, oxamate, or vehicle control. (A) Plasma anti-dsDNA IgG levels measured by ELISA. (B) Urinary protein-to-creatinine ratio quantified by Bradford assay. (C) Glomerular histopathology assessed by PAS staining. Scale bar: 20 μm. (D) Glomerular IgG deposition detected by immunofluorescence. Scale bar: 20 μm. Data in (A, B) are presented as mean ± SEM (n = 4 per group). ****$p < 0.0001$ with ANOVA plus Tukey's method (A-B).

Supplementary Figure 10

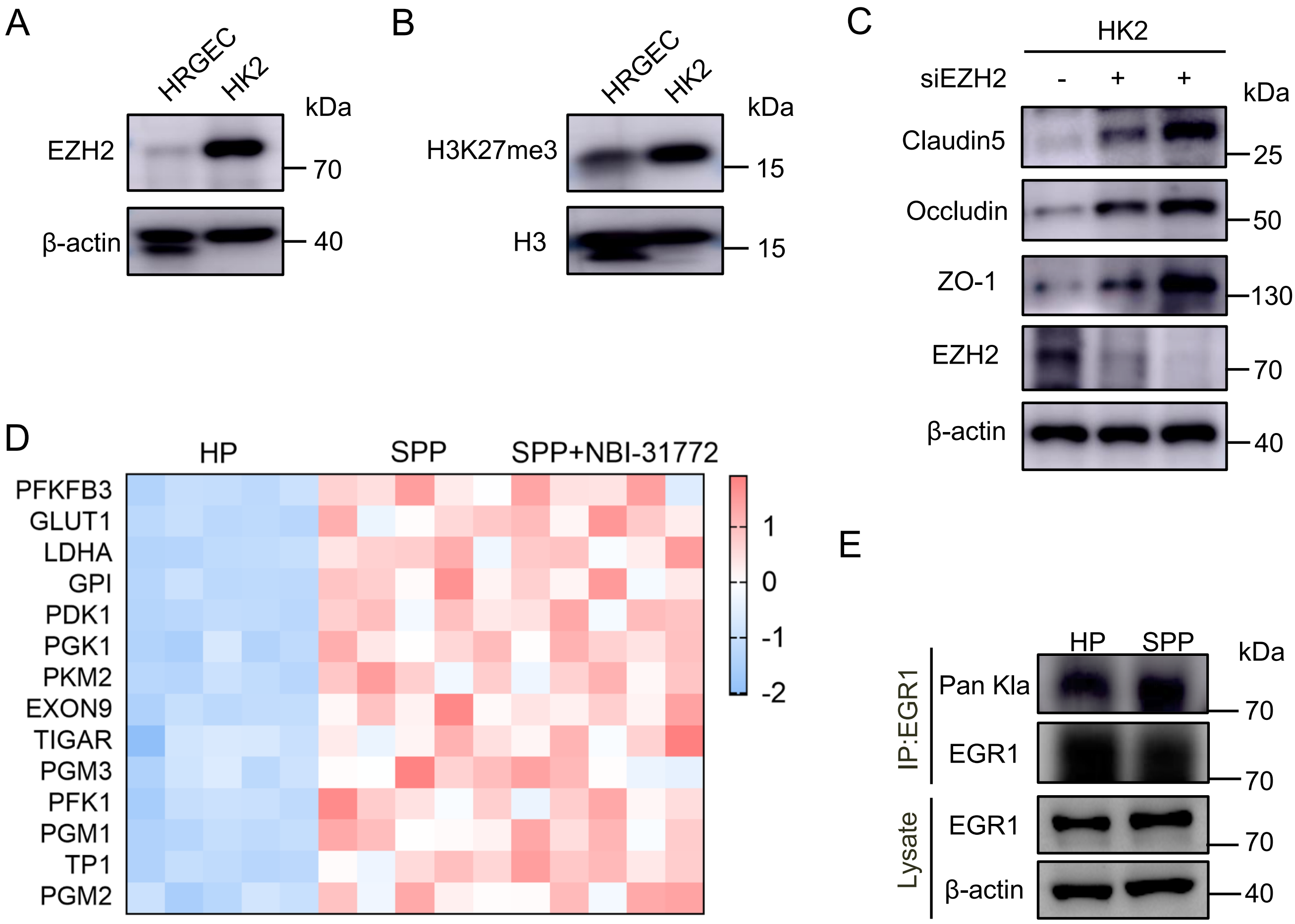

Fig. S10 Assessment of confounding factors in the HRGEC system

(A-B) EZH2 and H3K27me3 protein expression in HRGECs versus HK2 cells was assessed by western blot. (C) TJ-related protein expression was examined by western blot in HK2 cells following EZH2 knockdown. (D) HRGECs were treated with 10% plasma from HCs or SLE patients for 24 h, with or without NBI-31772 (100 nM) co-treatment. mRNA levels of glycolysis-related genes were measured by qPCR. (E) HRGECs were incubated with 10% HC or SLE plasma for 24 h. EGR1 lactylation was evaluated by immunoprecipitation followed by western blot.

Table S1. Features of SLE patients and healthy individuals

**Demographic parameters**	**SLE**	**Healthy**
No. of subjects	108	64
Sex (Female, %)	97 (89.8%)	57 (89.1%)
Age (mean ± SEM [years])	29.2±8.5	28.5±6.8
SLEDAI	13.2±3.8	N/A
Disease duration (mean ± SEM [months])	6.3±4.9	N/A
	Treatments	
Untreated (n, %)	49 (45.4%)	N/A
Corticosteroids (n, %)	41 (38.0%)	N/A
Antimalarials (n, %)	57 (52.8%)	N/A
Immunosuppressants (Cyclophosphamide, mycophenolate mofetil and calcineurin inhibitors, n, %)	33 (30.6%)	N/A

Table S2. Sequence of primers targeting different genes for qPCR

Oligo name	Forward primer (5' to 3')	Reverse primer (5' to 3')
β-actin	AGTCCCTGCCCTTTGTACACA	GATCCGAGGGCCTCACTAAAC
ZO-1	CAGCCGGTCACGATCTCCT	TCCGGAGACTGCCATTGC
Occludin	CAGGAACCGAGAGCCAGGT	ATAAACCAATCTGCTGCGTCCTA
Claudin-5	CTGCTGGTTCGCCAACATT	TGCGACACGGGCACAG
VE-cadherin	GCGACTACCAGGACGCTTTCA	CATGTATCGGAGGTCGATGGTG
ICAM-1	GCAAGAAGATAGCCAACCA	TGCCAGTTCCACCCGTTC
ICAM-2	ATGACACGGTCCTCCAATGCCA	GCACTCAATGGTGAAGGACTTGC
VCAM-2	CATGACCTGTTCCAGCGAGG	CATTCACGAGGCCACCACTC
HK1	GCTCTCCGATGAAACTCTCATAG	GGACCTTACGAATGTTGGCAA
PFK1	GGTGCCCGTGTTCTTCTTGT	AAGCATCATCGAAACGCTCTC
PGK1	CCACTGGTGCTTCTGGCATA	ATGAGAGCTTTGGTTCCCCG
LDHA	GGAGATCCATCATCTCTCCC	GGCTGTOGCCATCAGTATCT
PGM1	GGAGGAGGAGCTGGAGAAGT	CCTTCATCAGCAGATCATTTC
PGM2	CAGCCAGAGGAGCAAGTAAG	GTCTTGGCATTCTGGTGATG
GLUT3	GCTGGGCATCGTGTTGTGA	GCACTTTGTAGGATACGAGGAAG
GPI	GCTGAACGTTGTCAAACCCCC	AAACATGTTCGAGTTCTGGGA
GLUT1	TTGCAGGCTTCTCCAACTGGAC	CAGAACCAGGAGCACAGTGAAG
PKM2	ATGGCTGACACATTCCTGGAGC	CCTTCAACGTCTCCACTGATCG
TIGAR	CCAAAGCAGCCAGGGAAGAGTG	CCGCTTCTTTCAGGATTAGTTGAC
LDHB	GGACAAGTTGGTATGGCGTGTG	AAGCTCCCATGCTGCAGATCCA
PDK1	CATGTCACGCTGGGTAATGAGG	CTCAACACGAGGTCTTGGTGCA
PDK3	CGCTCTCCATCAAACAATTCCT	CCACTGAAGGGCGGTTAAGTA-3
PFKM	GCTTCTAGCTCATGTCAGACCC	CCAATCCTCACAGTGGAGCGAA
PFKFB3	GGCAGGAGAATGTGCTGGTCAT	CATAAGCGACAGGCGTCAGTTTC
PGM3	GGAGGCAATCAATGACCTGGTG	CCAAGCTCACTTCATGTGCAAGG
PKM1 (exon9)	TGCAGCACCTGATAGCTCGG	GTGAGCACTCCTGCCAGACTC
18SrRNA	AGTCCCTGCCCTTTGTACACA	GATCCGAGGGCCTCACTAAAC
DLAT	GTTGATGTCAGTGTTGCGGT	CCCTGGAATTCACTGTGCTG
DLD	CCGACTTCATCCTGATACACT	TTAGTGGTAACGAGTCAGGA
PDHA	GATGAGCAGACTAGTACCGC	CCGGTAGCTGTGATGAGAAG
EZH2	AGGACGGCTCCTCTAACCAT	CTTGGTGTTGCACTGTGCTT
RACK1	AGCAGCAACCCCTATCATCGTC	TGAGATCCCATAACATGGCCT
PLVAP	CCGGGTCATCTACACGAACAA	CCTTGAATTGATCTCTGCATTGC
