# Supplementary material for: Lactate controls glomerular endothelial barrier integrity in lupus nephritis
Source: JCI Insight. 2026 Aug 18;11(18):e205034. doi: 10.1172/jci.insight.205034 (PMC13596713; doi:10.1172/jci.insight.205034)
Supplement: Supplementary file 2 [file jciinsight-11-205034-s075.pdf]

1E

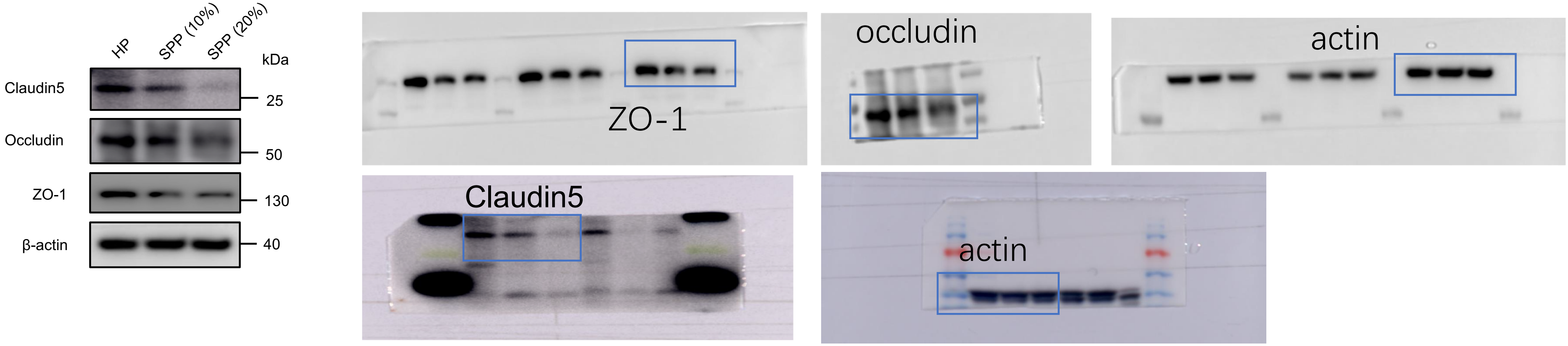
HP
SPP (10%)
SPP (20%)
kDa
Claudin5
Occludin
ZO-1
β-actin
25
50
130
40
ZO-1
occludin
actin
Claudin5
actin

2L

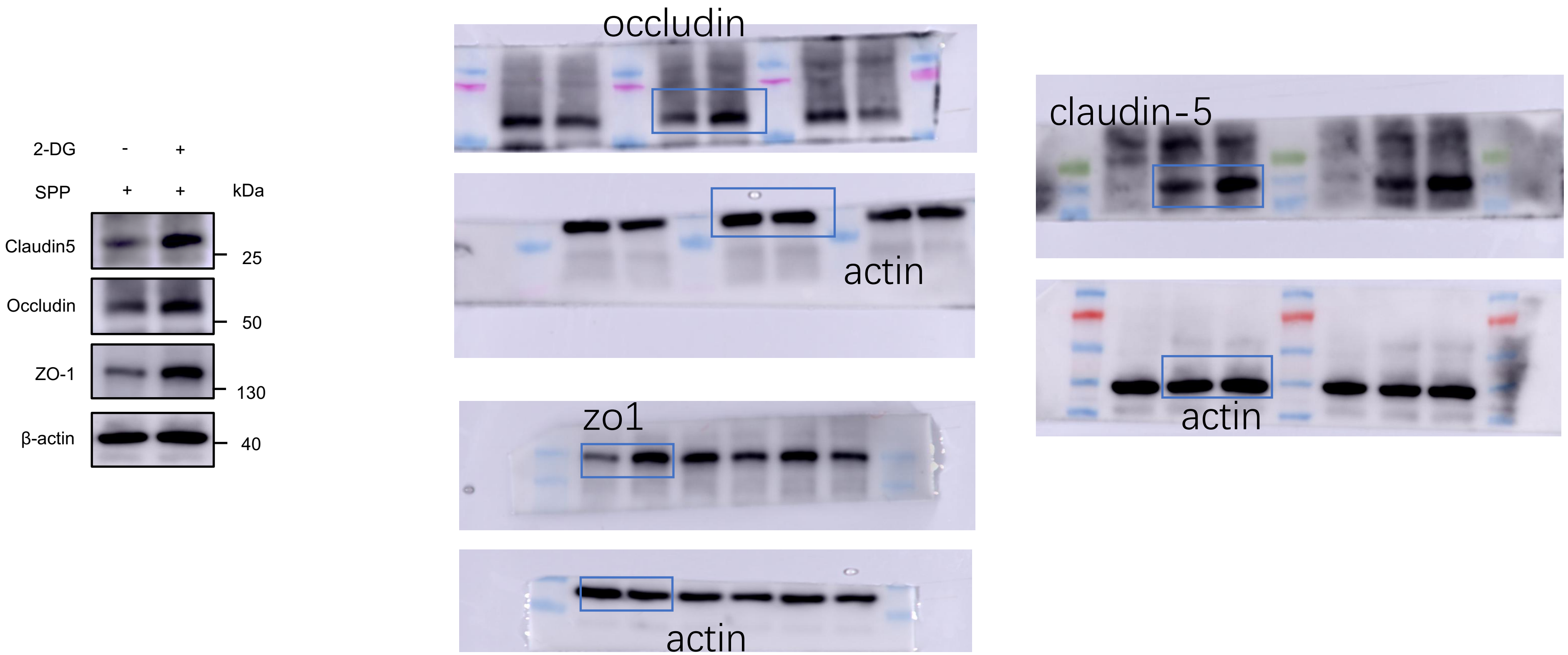
2-DG - +
SPP + +
kDa
Claudin5
Occludin
ZO-1
β-actin
25
50
130
40
occludin
actin
claudin-5
actin
zo1
actin

2O

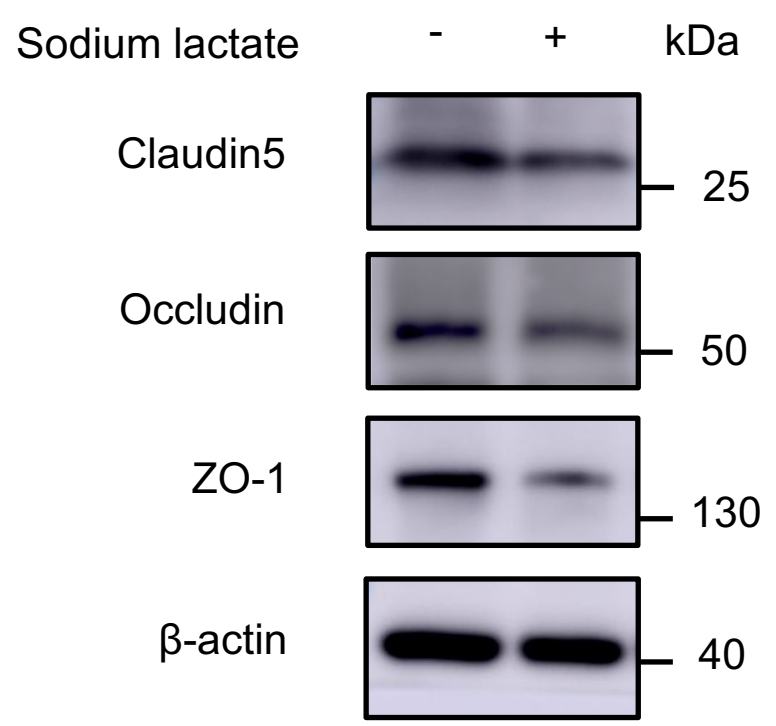

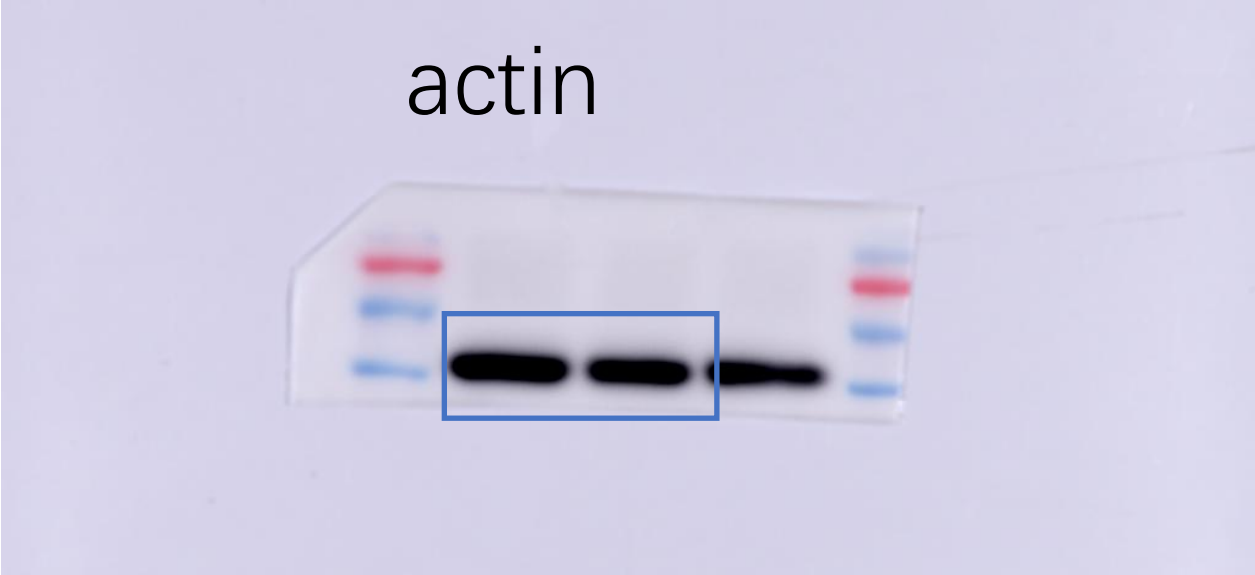

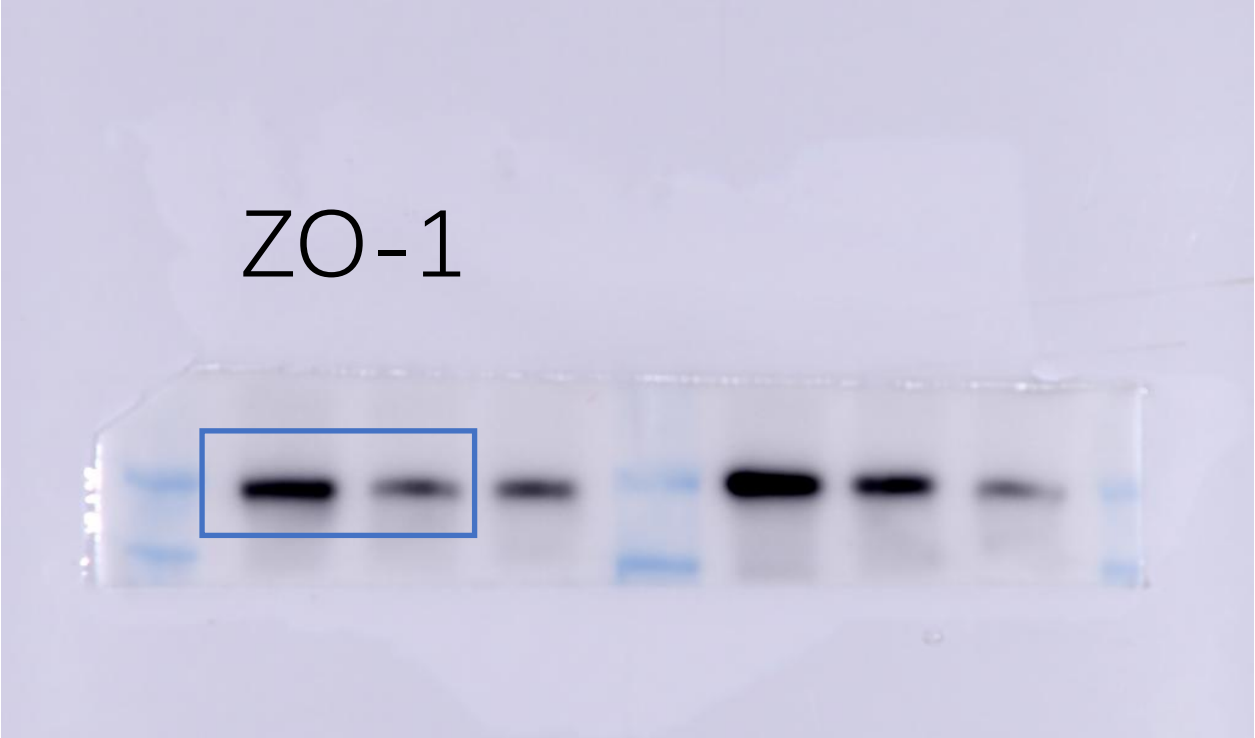

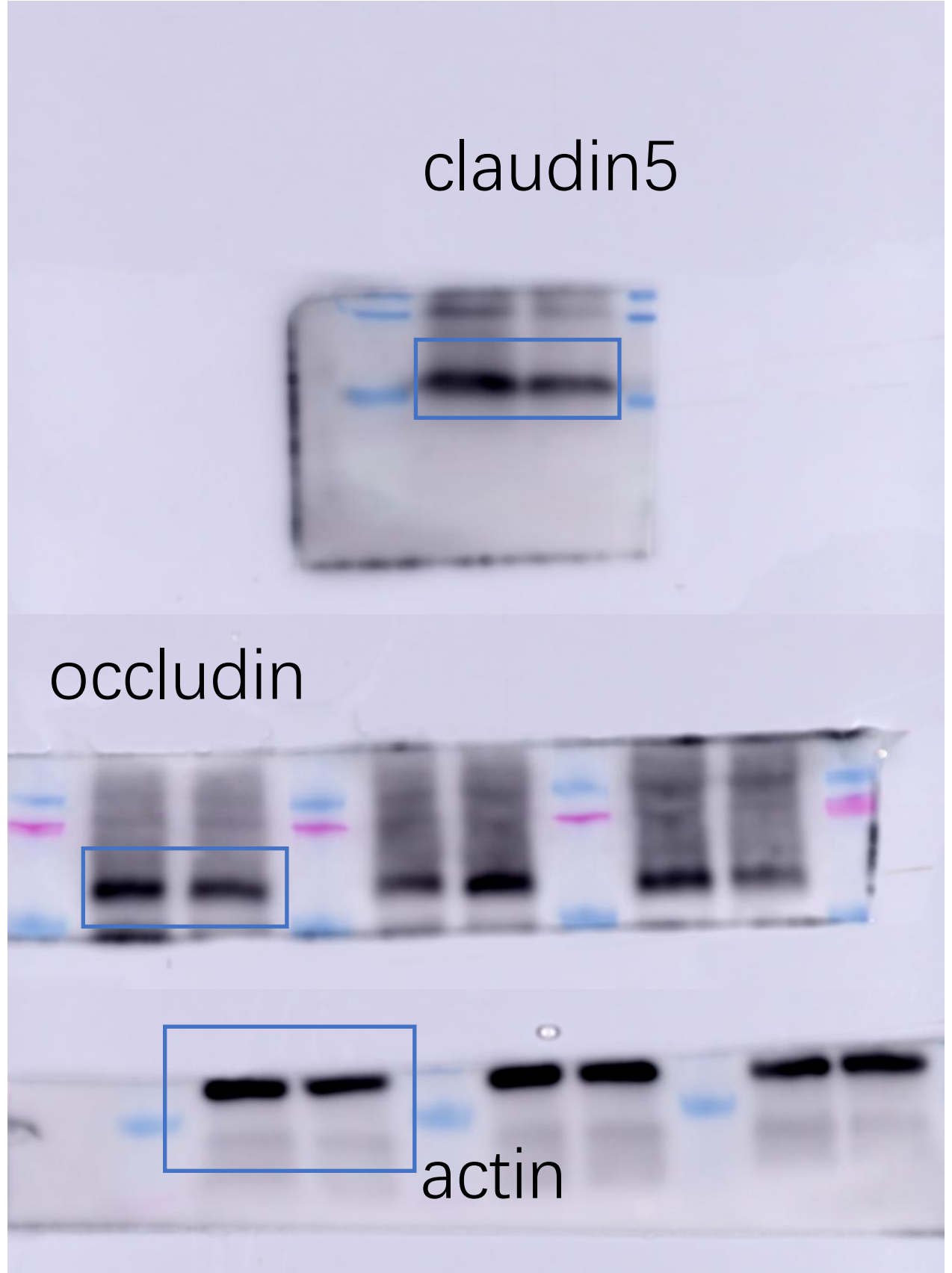

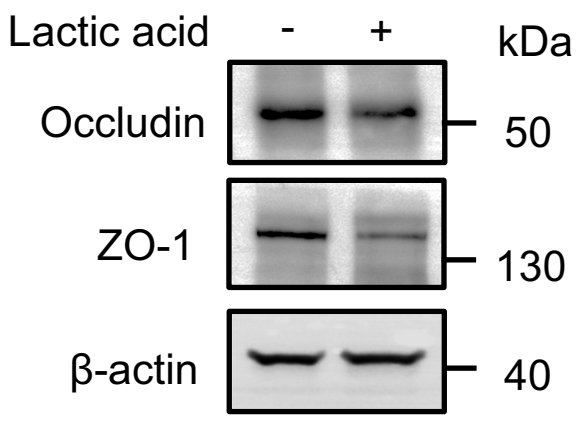

ZO-1

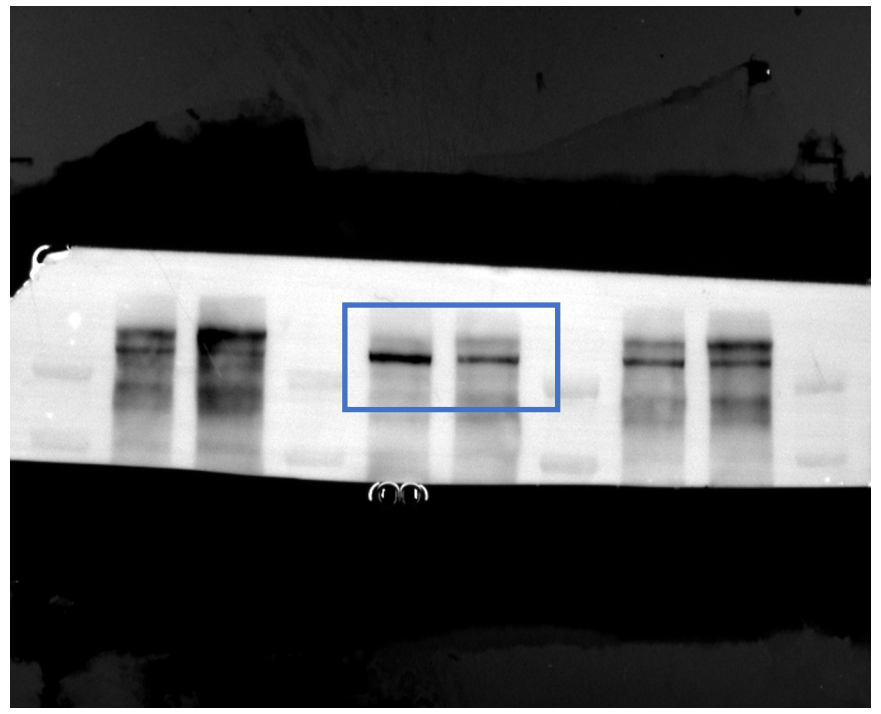

occludin

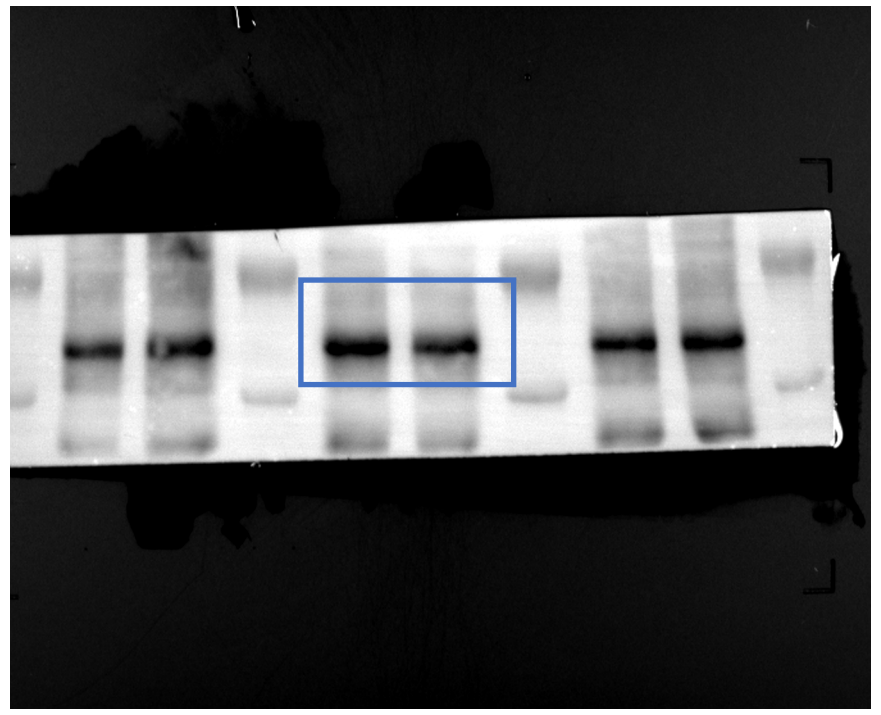

actin

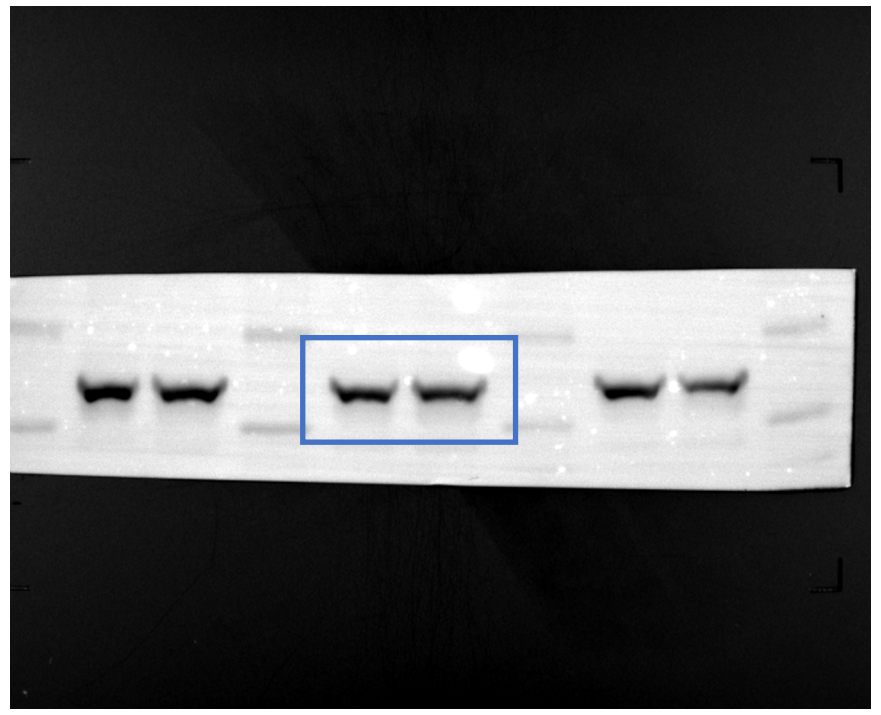

2Q

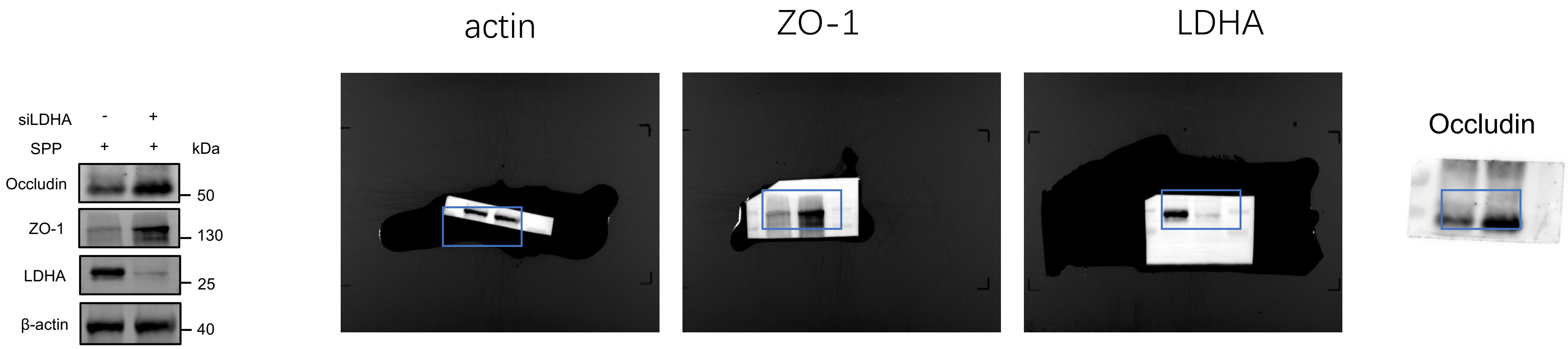
actin
ZO-1
LDHA
Occludin
siLDHA - +
SPP + +
kDa
Occludin
50
ZO-1
130
LDHA
25
β-actin
40

2S

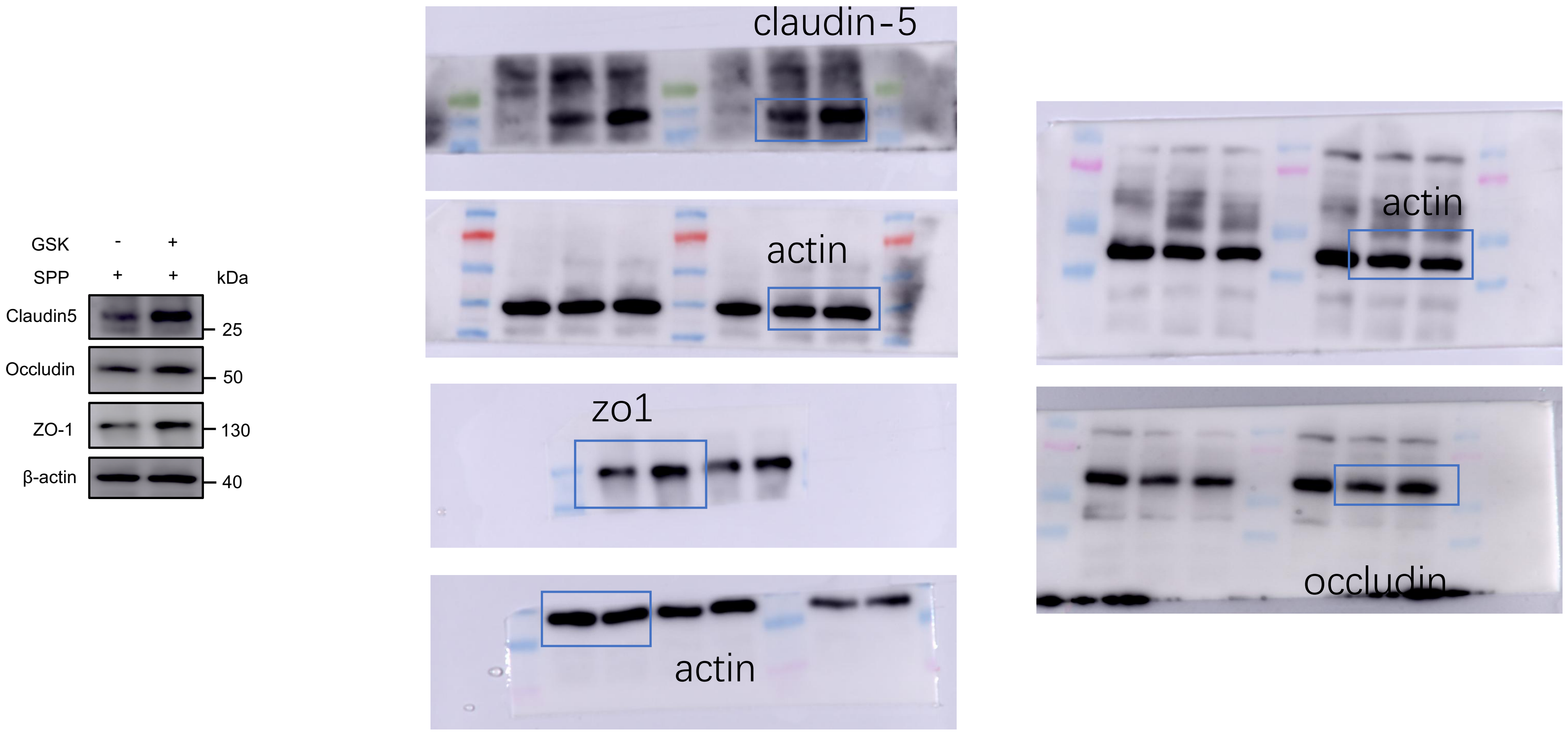
GSK - +
SPP + +
kDa
Claudin5
25
Occludin
50
ZO-1
130
β-actin
40
claudin-5
actin
zo1
actin
actin
occludin

3A

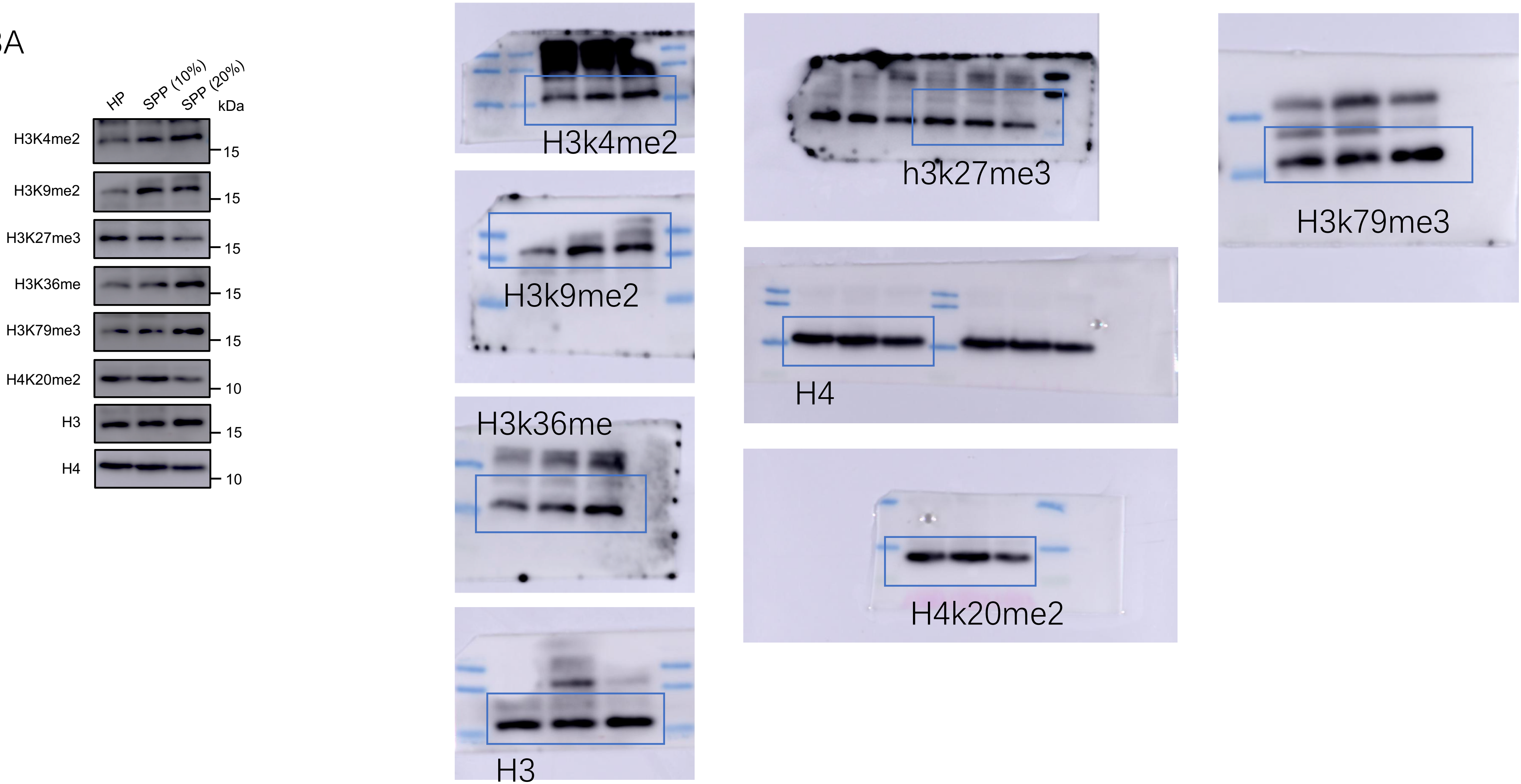
HP
SPP (10%)
SPP (20%)
kDa
H3K4me2
15
H3K9me2
15
H3K27me3
15
H3K36me
15
H3K79me3
15
H4K20me2
10
H3
15
H4
10
H3k4me2
h3k27me3
H3k79me3
H3k9me2
H4
H3k36me
H4k20me2
H3

3B

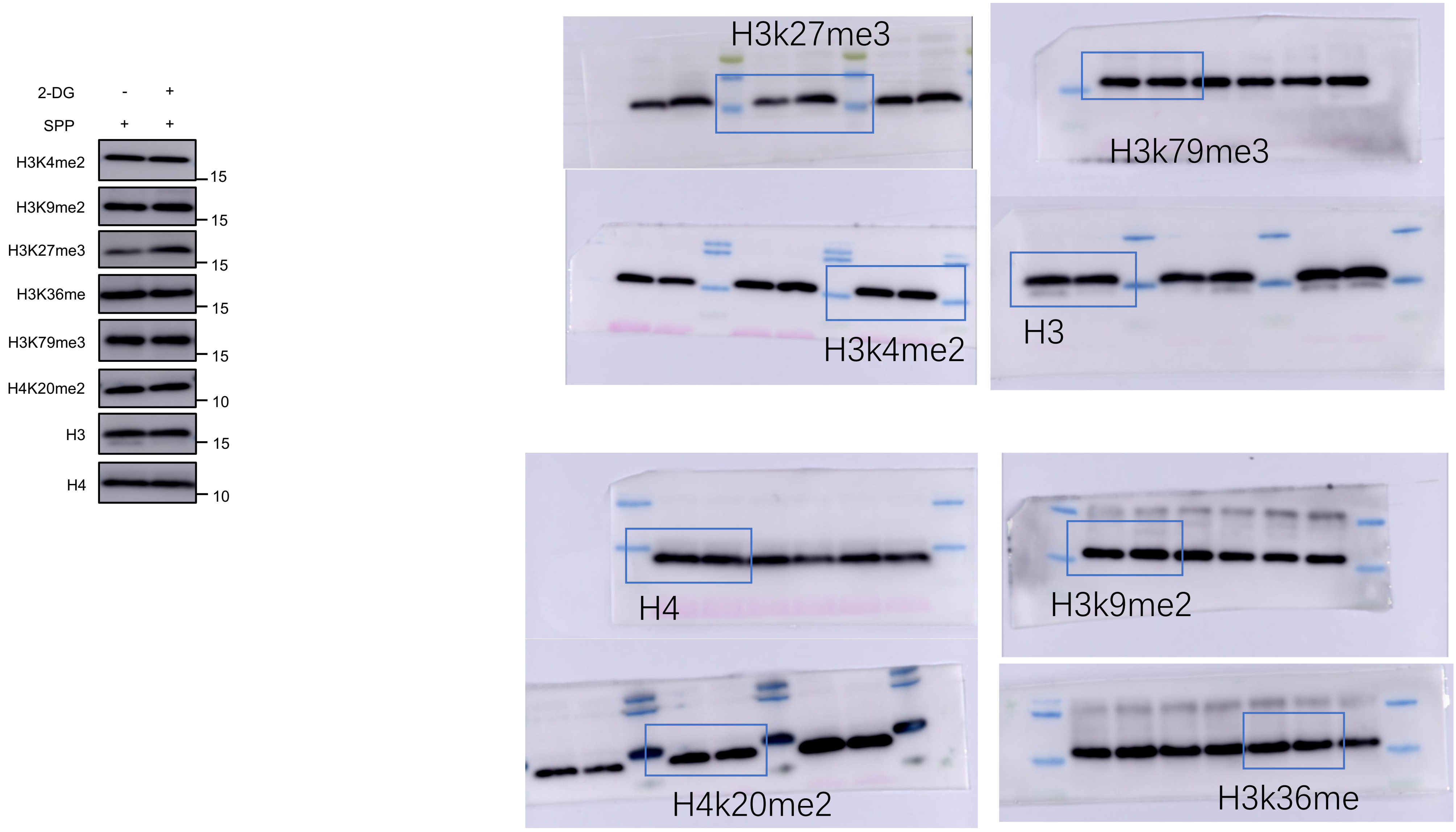

2-DG - +
SPP + +
H3K4me2
H3K9me2
H3K27me3
H3K36me
H3K79me3
H4K20me2
H3
H4
15
15
15
15
15
10
15
10
H3k27me3
H3k79me3
H3k4me2
H3
H4
H3k9me2
H4k20me2
H3k36me

3C

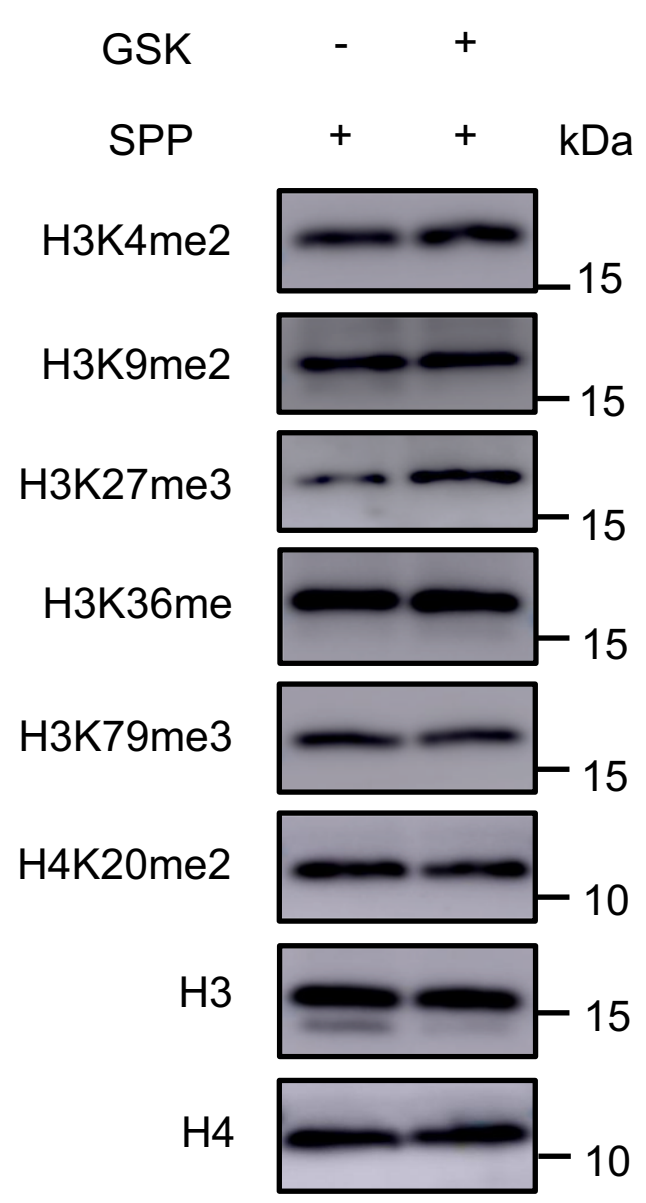

GSK - +
SPP + +
kDa
H3K4me2
15
H3K9me2
15
H3K27me3
15
H3K36me
15
H3K79me3
15
H4K20me2
10
H3
15
H4
10

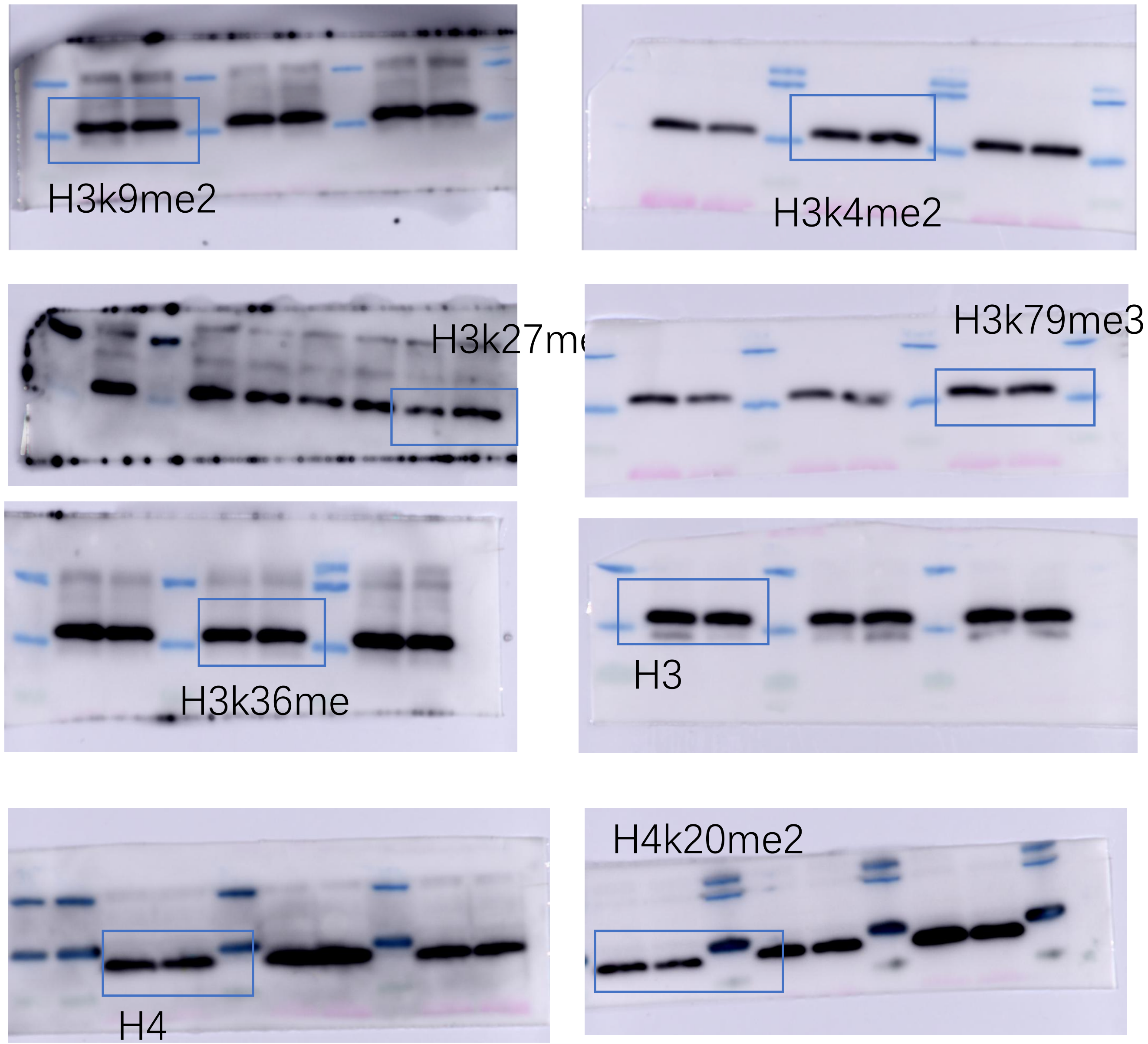

H3k9me2
H3k4me2
H3k27m
H3k79me3
H3k36me
H3
H4
H4k20me2

3D

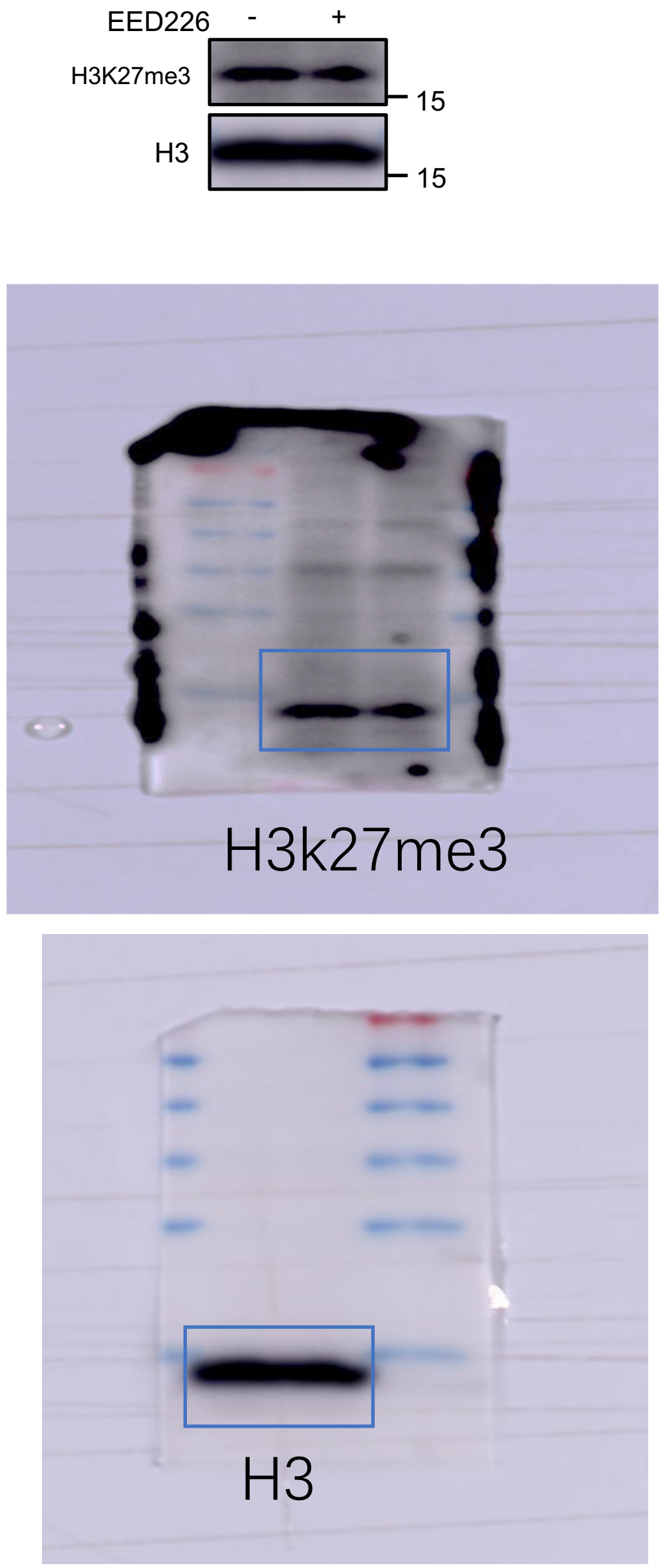
EED226 - +
H3K27me3
15
H3
15
H3k27me3
H3

3F

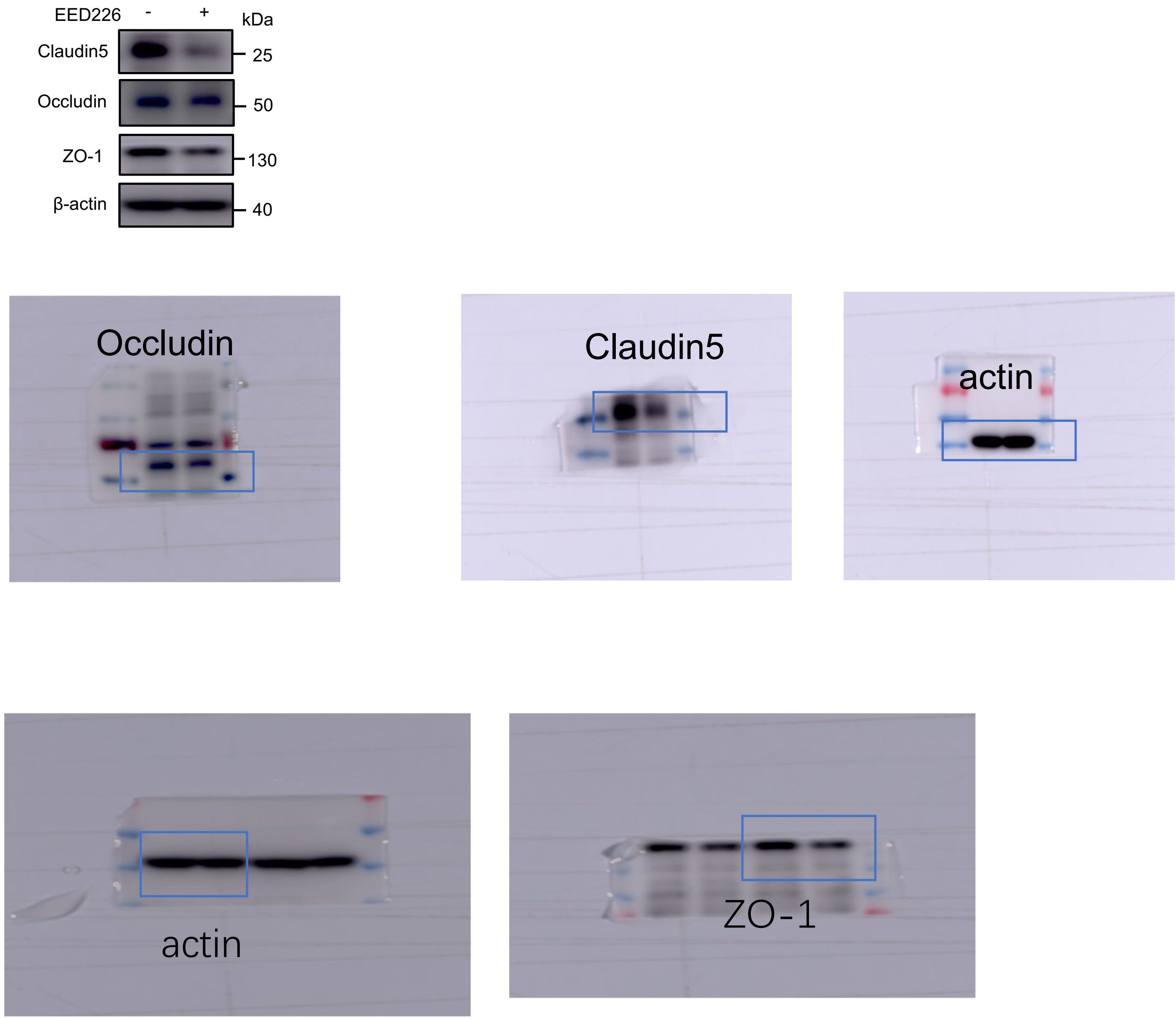
EED226 - +
kDa
Claudin5
25
Occludin
50
ZO-1
130
β-actin
40
Occludin
Claudin5
actin
actin
ZO-1

3G

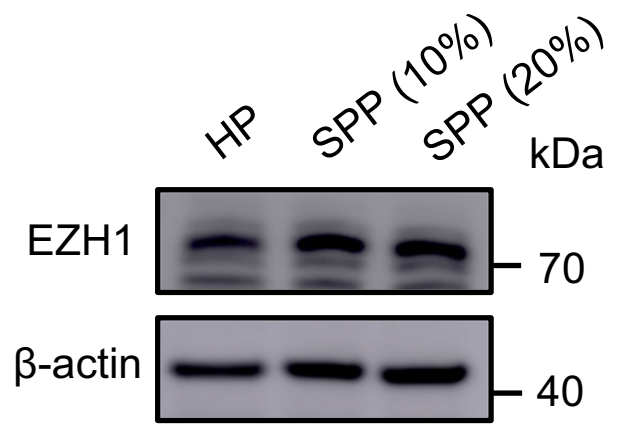
HP
SPP (10%)
SPP (20%)
kDa
EZH1
70
β-actin
40

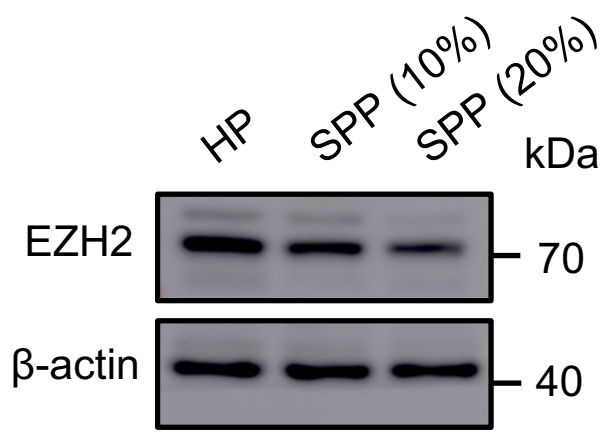
HP
SPP (10%)
SPP (20%)
kDa
EZH2
70
β-actin
40

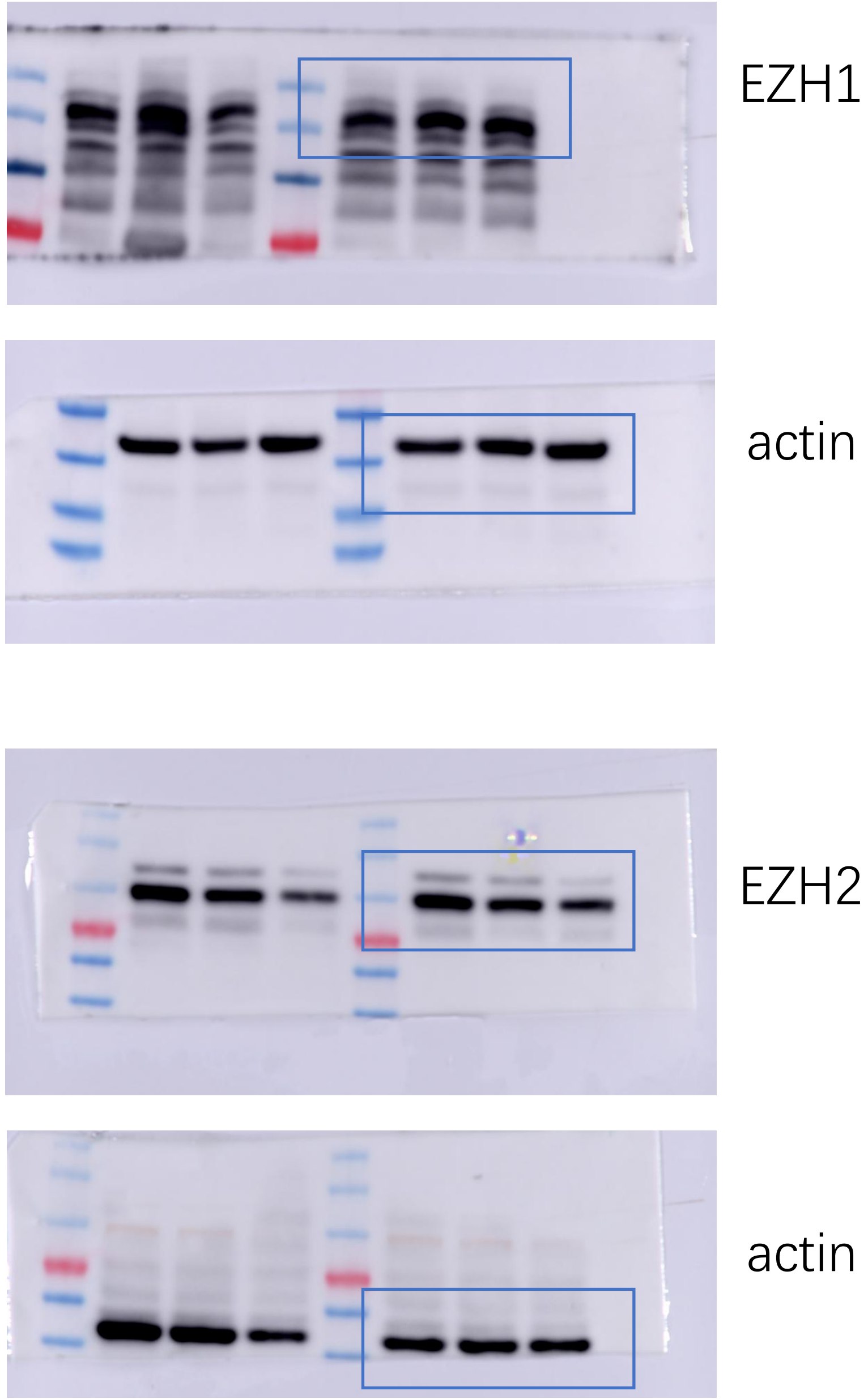
EZH1
actin
EZH2
actin

3H

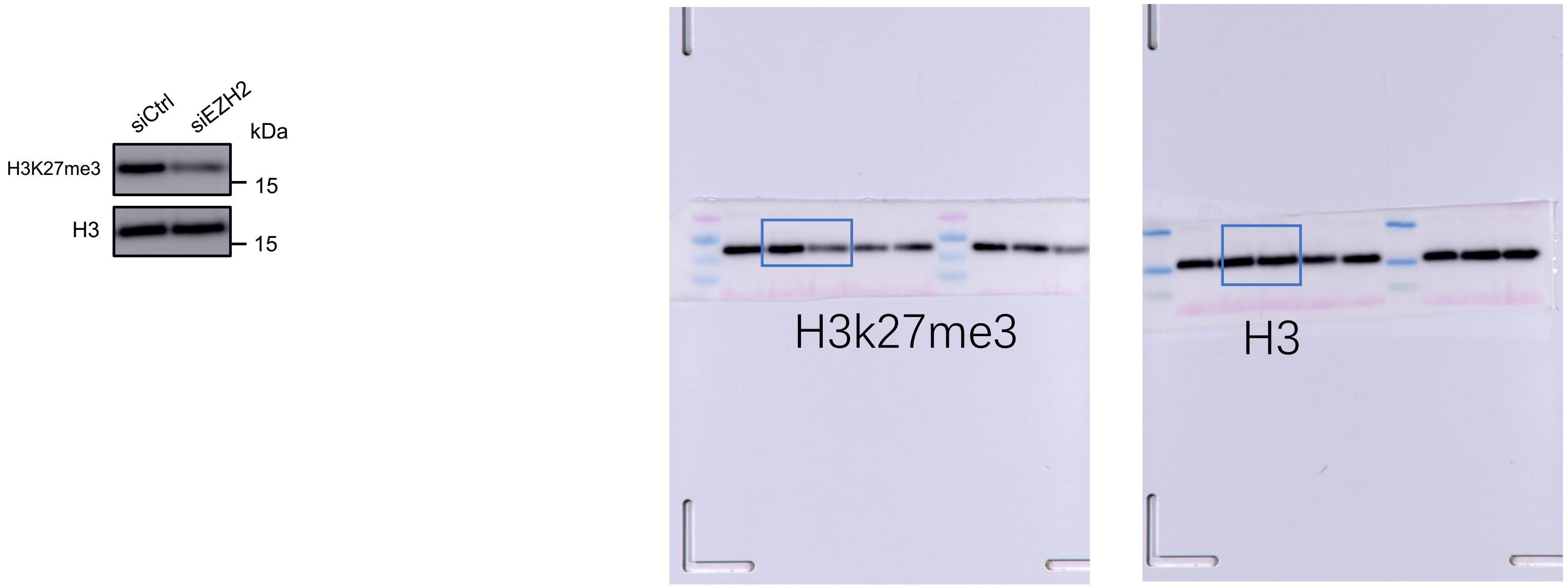
siCtrl
siEZH2
kDa
H3K27me3
15
H3
15
H3k27me3
H3

3I

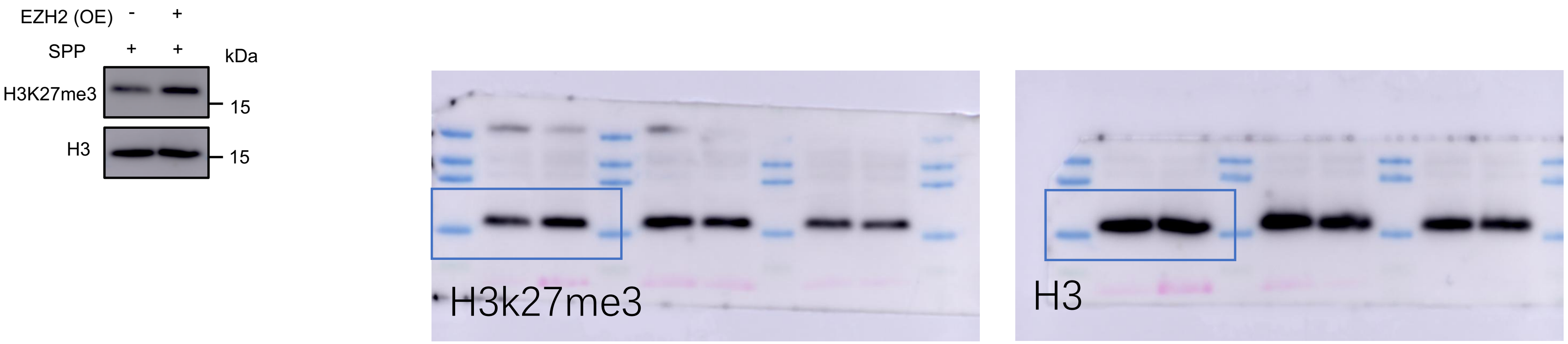
EZH2 (OE) - +
SPP + +
kDa
H3K27me3
15
H3
15
H3k27me3
H3

3J

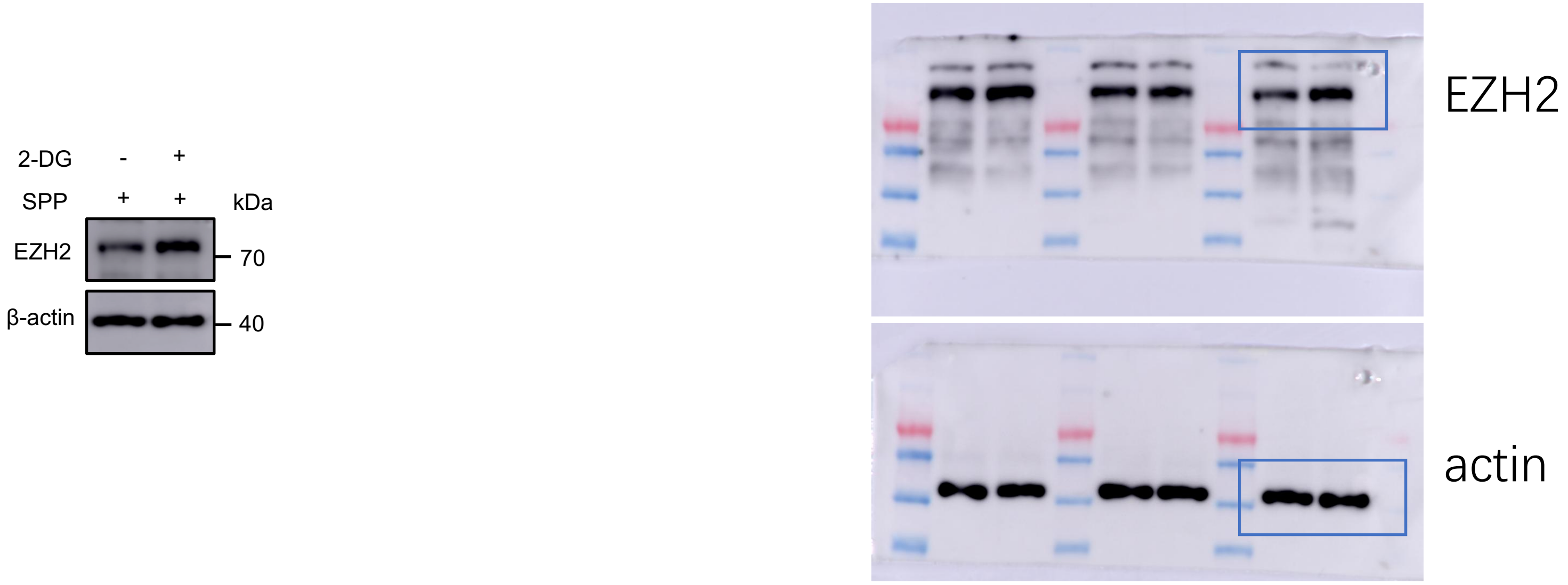
2-DG - +
SPP + +
kDa
EZH2
70
β-actin
40
EZH2
actin

3K

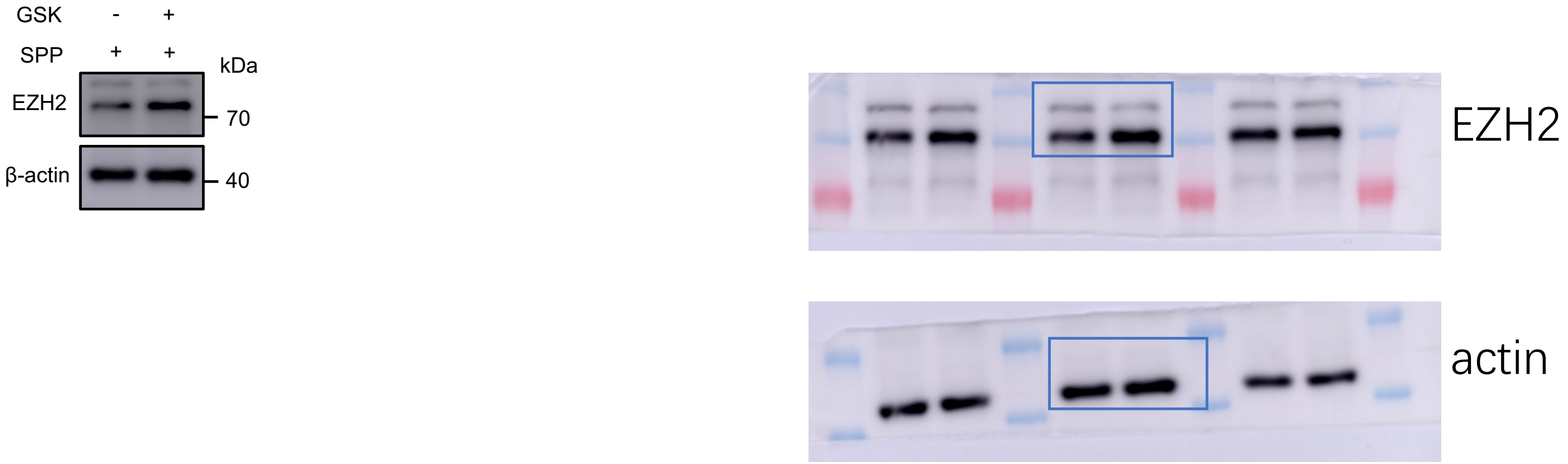
GSK - +
SPP + +
kDa
EZH2
70
β-actin
40
EZH2
actin

3L

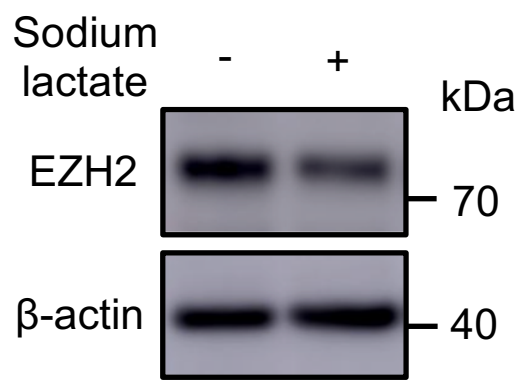
Sodium lactate
-
+
kDa
EZH2
70
β-actin
40

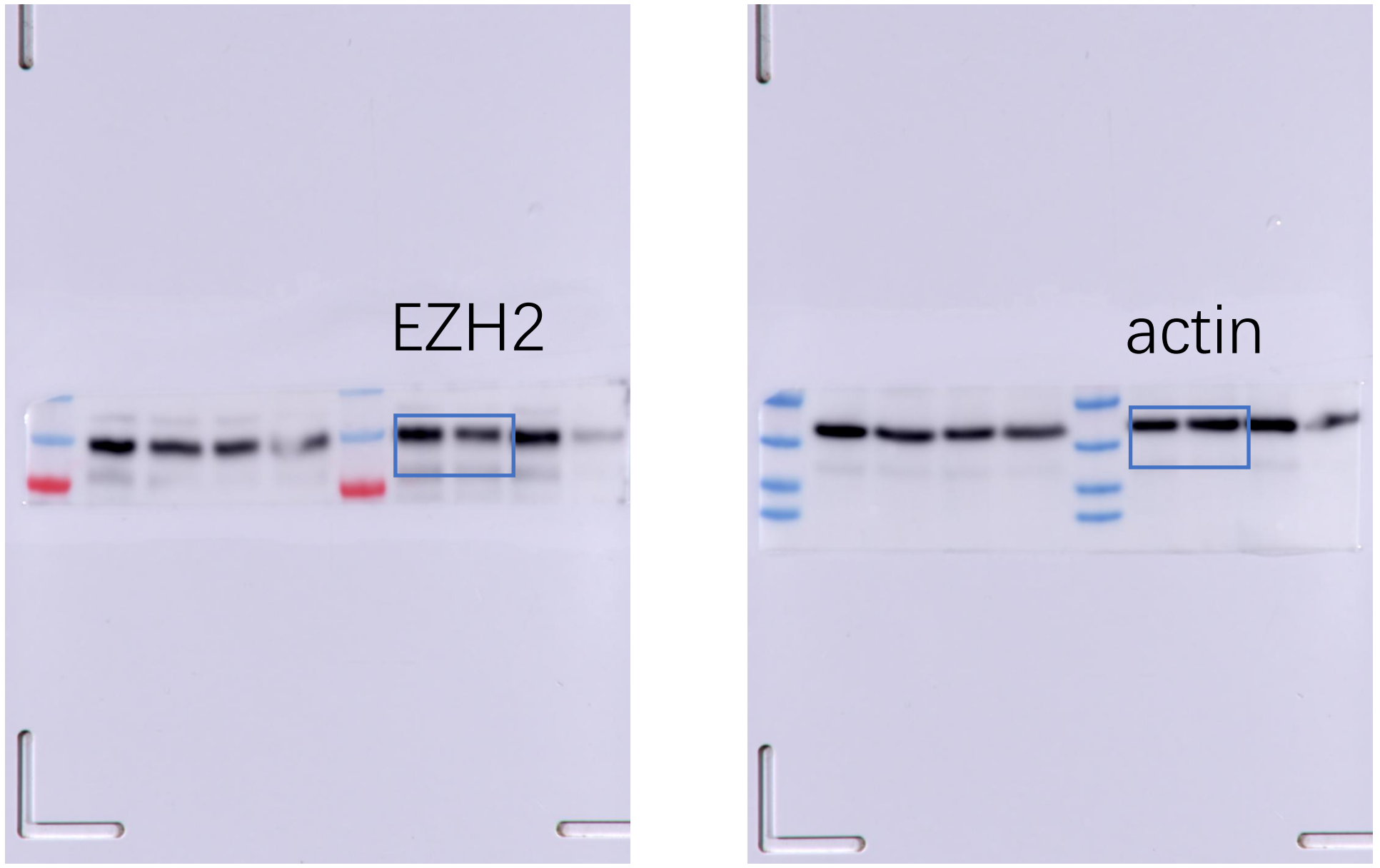
EZH2
actin

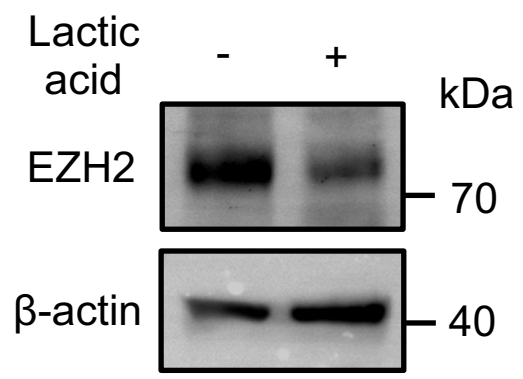
Lactic acid
-
+
kDa
EZH2
70
β-actin
40

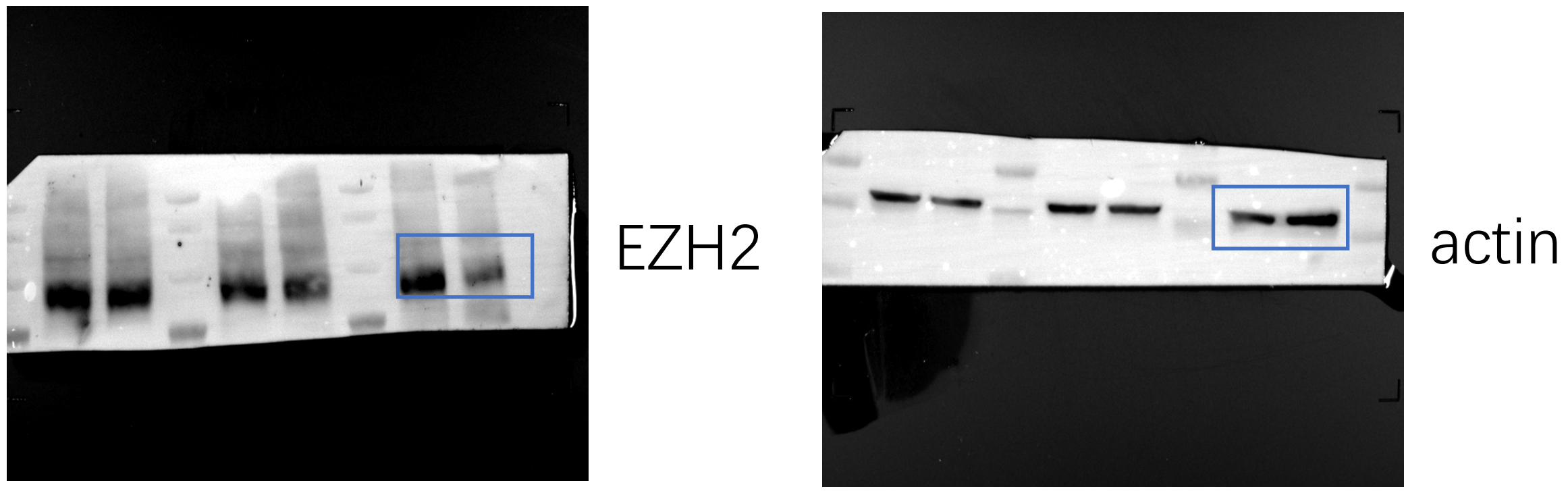
EZH2
actin

3N

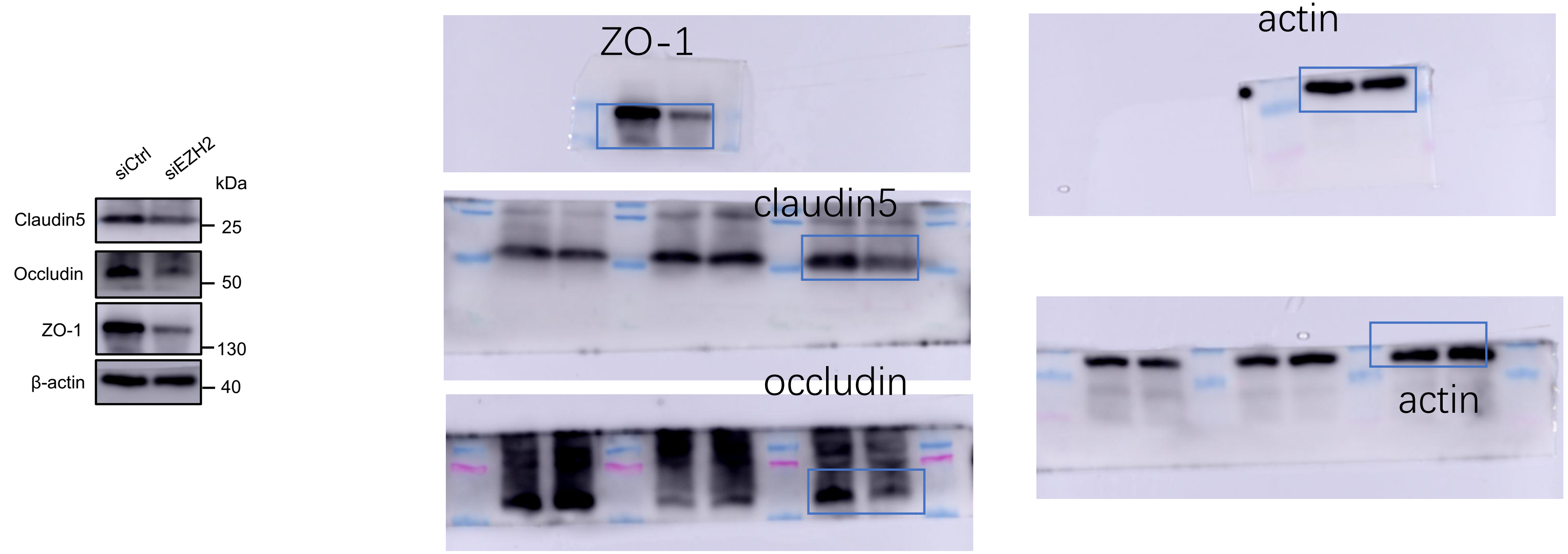
siCtrl
siEZH2
kDa
Claudin5
25
Occludin
50
ZO-1
130
β-actin
40
ZO-1
actin
claudin5
occludin
actin

3P

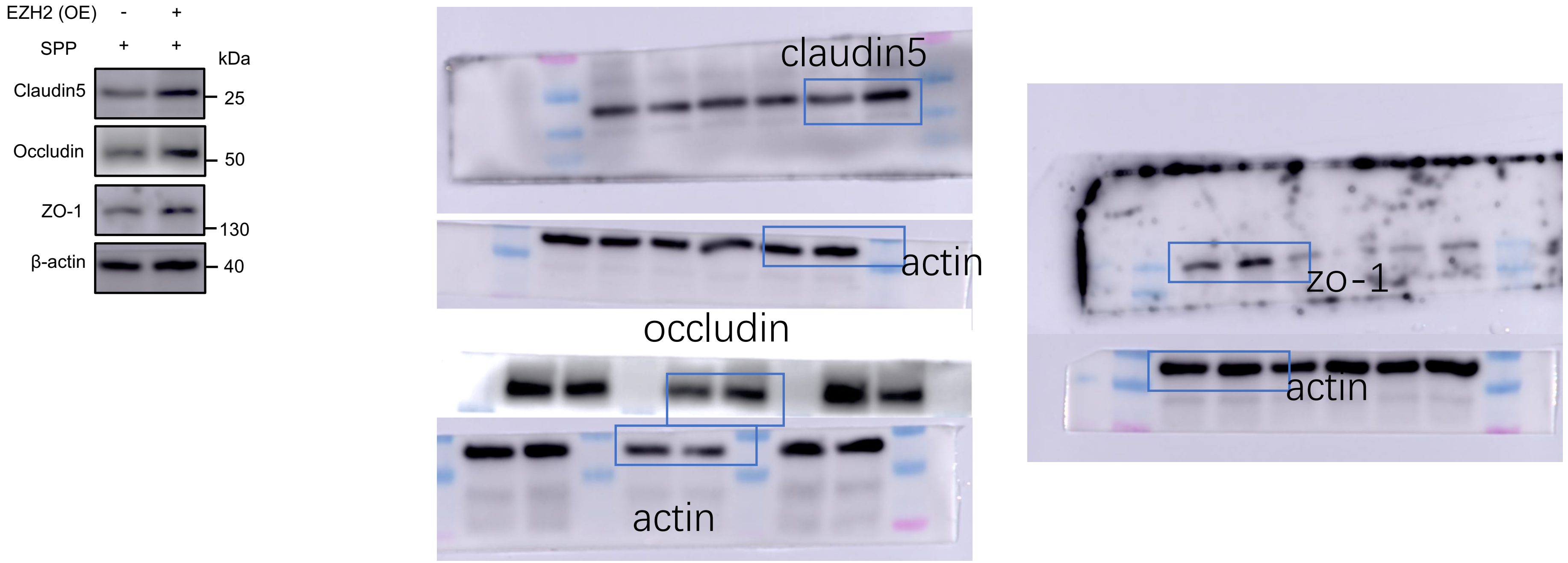
EZH2 (OE) - +
SPP + +
kDa
Claudin5
25
Occludin
50
ZO-1
130
β-actin
40
claudin5
actin
occludin
actin
zo-1
actin

3R

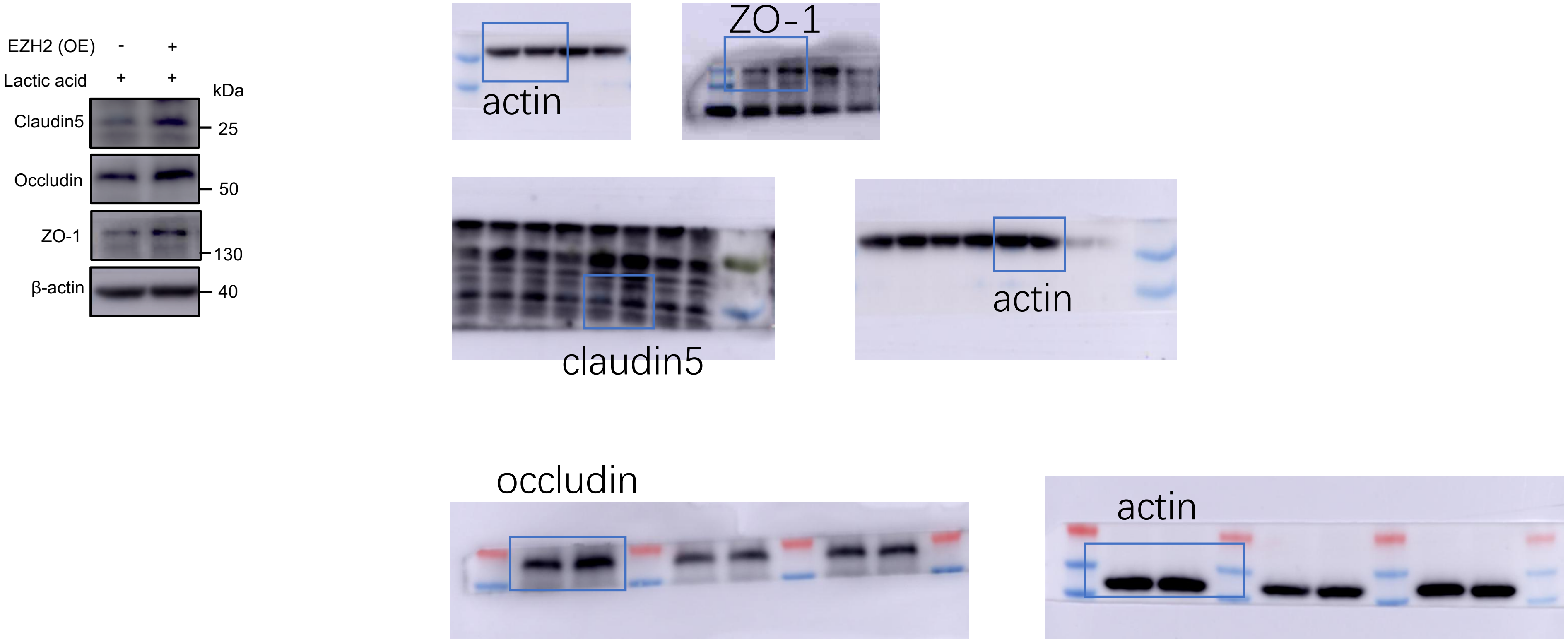

EZH2 (OE)
-
+
Lactic acid
+
+
kDa
Claudin5
25
Occludin
50
ZO-1
130
β-actin
40
actin
ZO-1
claudin5
actin
occludin
actin

4A

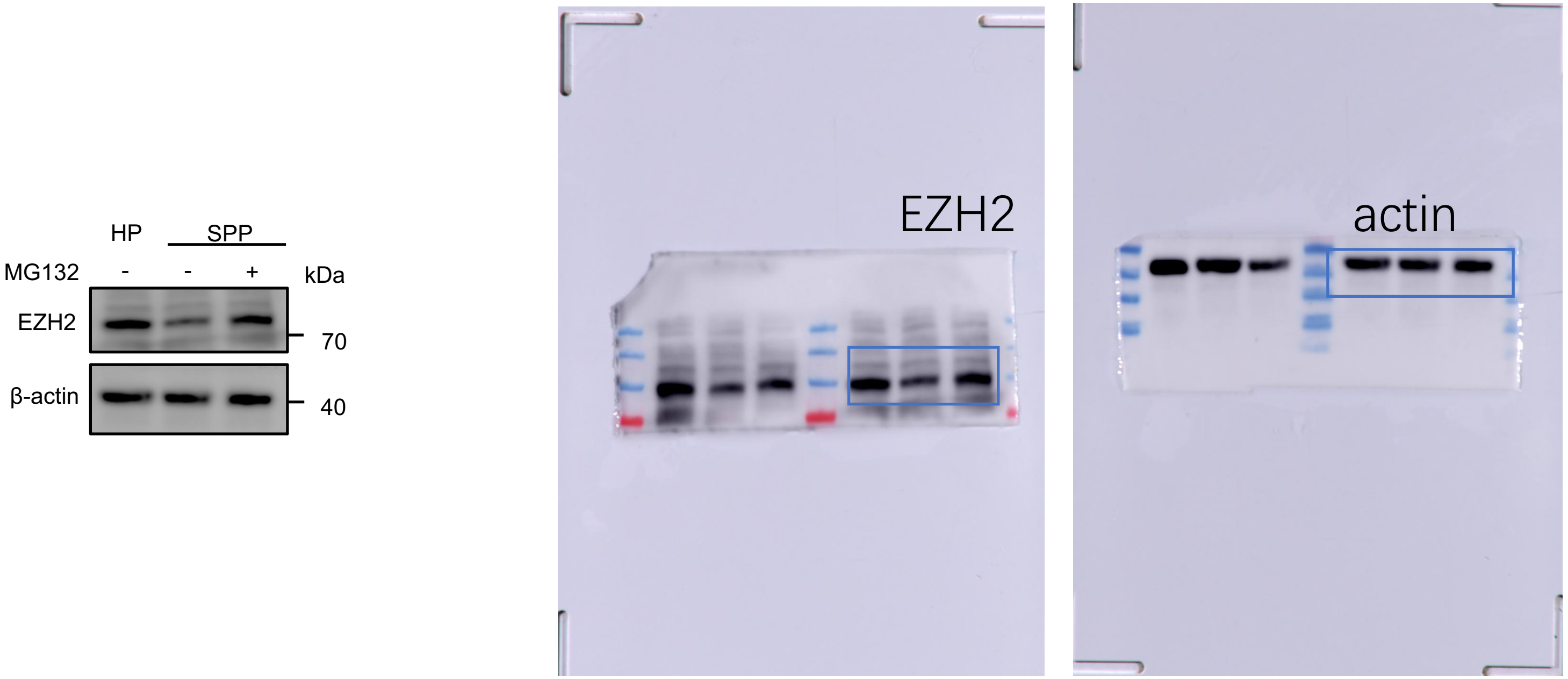

HP
SPP
MG132
-
-
+
kDa
EZH2
70
β-actin
40
EZH2
actin

4C

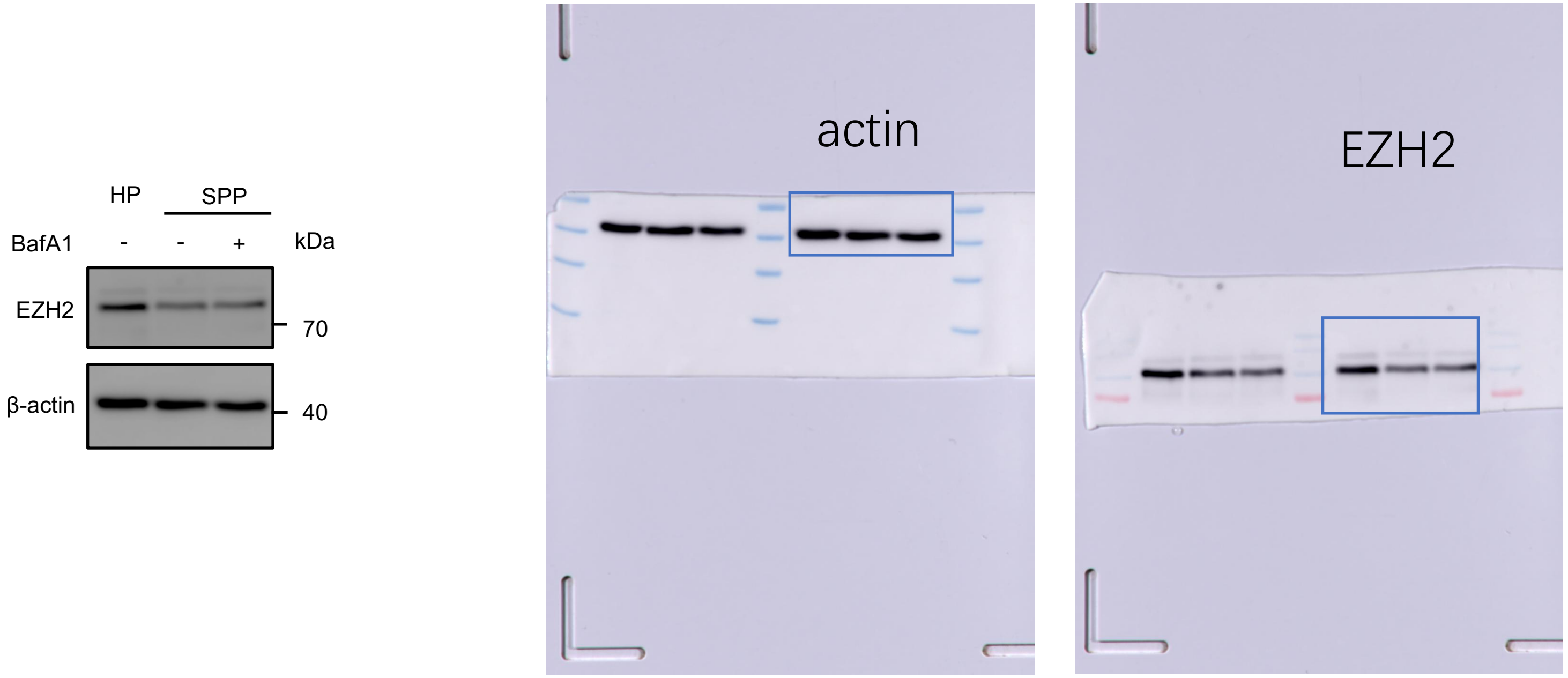

HP
SPP
BafA1
-
-
+
kDa
EZH2
70
β-actin
40
actin
EZH2

4D

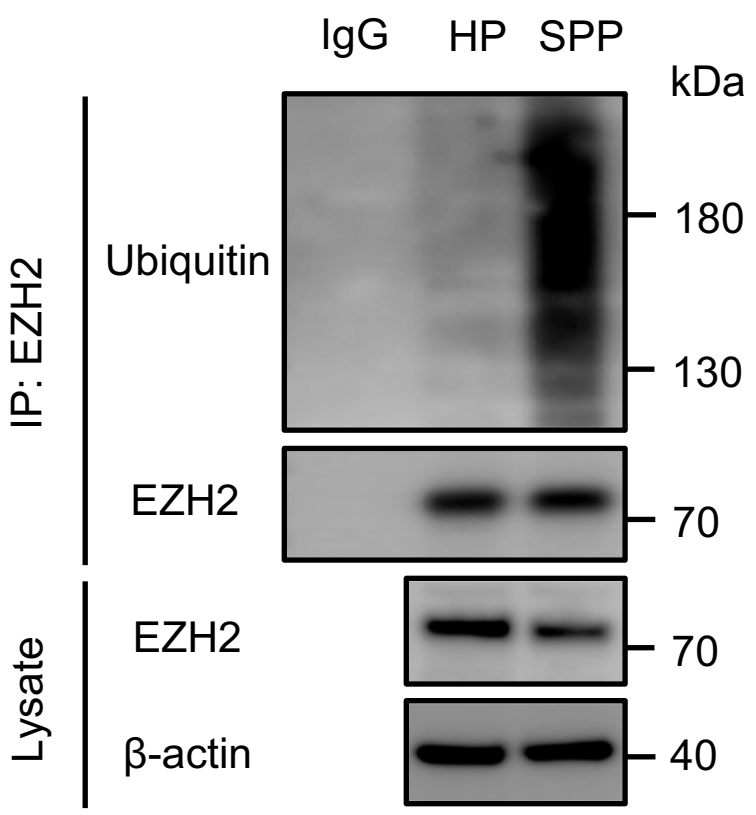
IgG
HP
SPP
kDa
IP: EZH2
Ubiquitin
EZH2
Lysate
EZH2
β-actin
180
130
70
70
40

4E

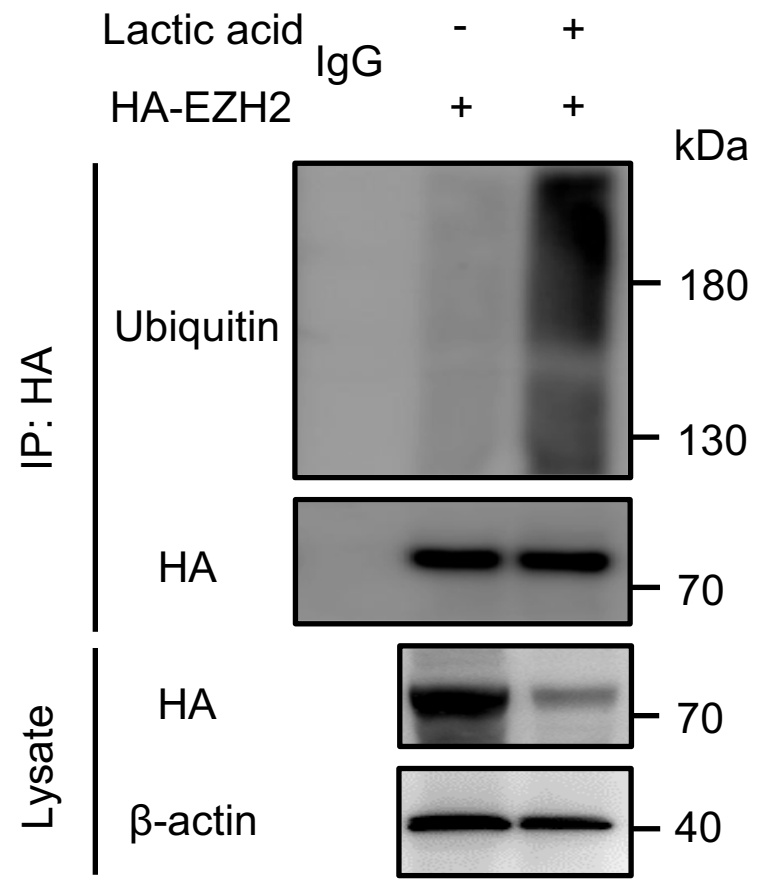
Lactic acid
IgG
HA-EZH2
-
+
+
+
kDa
IP: HA
Ubiquitin
HA
Lysate
HA
β-actin
180
130
70
70
40

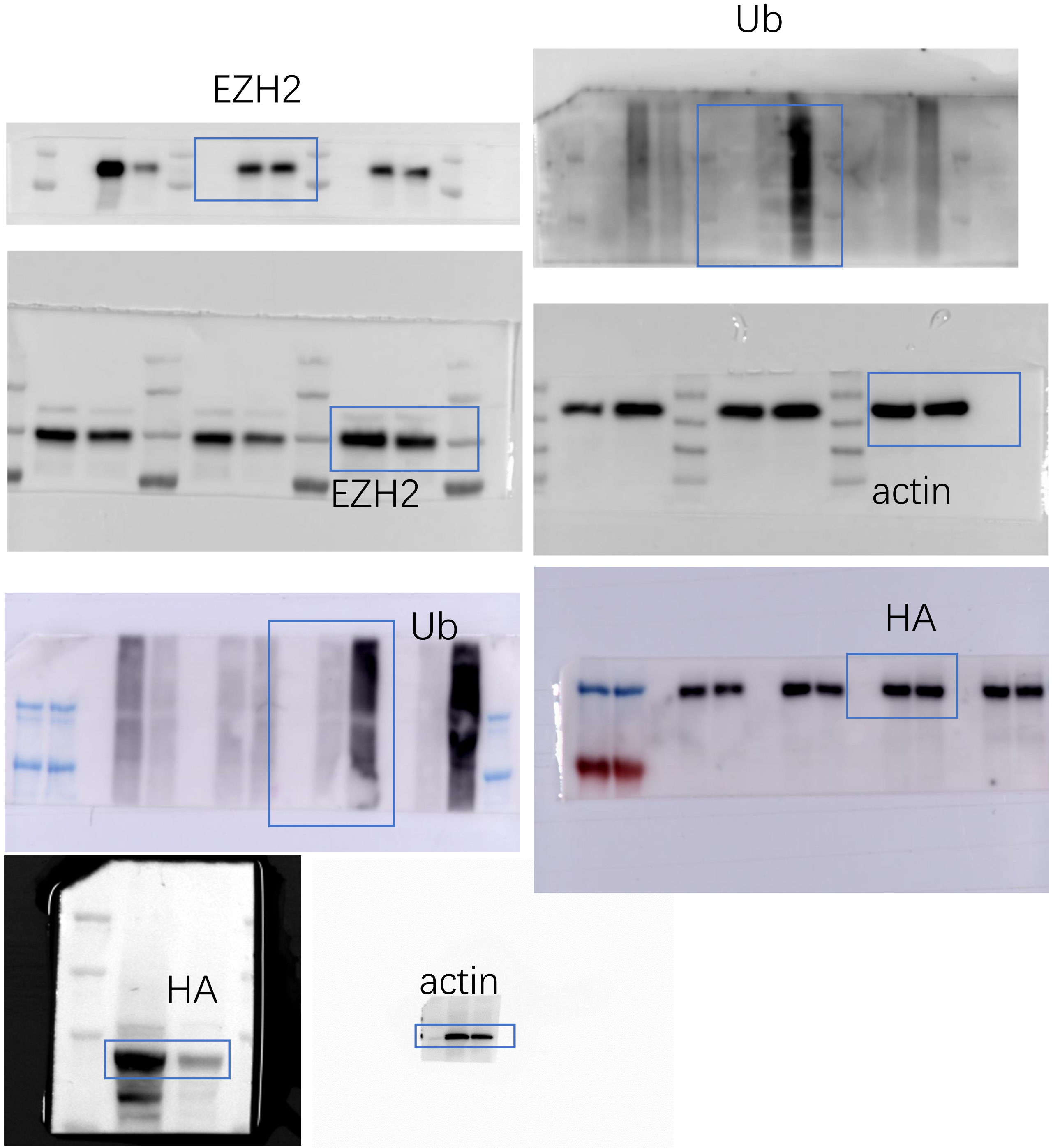
EZH2
Ub
EZH2
actin
Ub
HA
HA
actin

4F

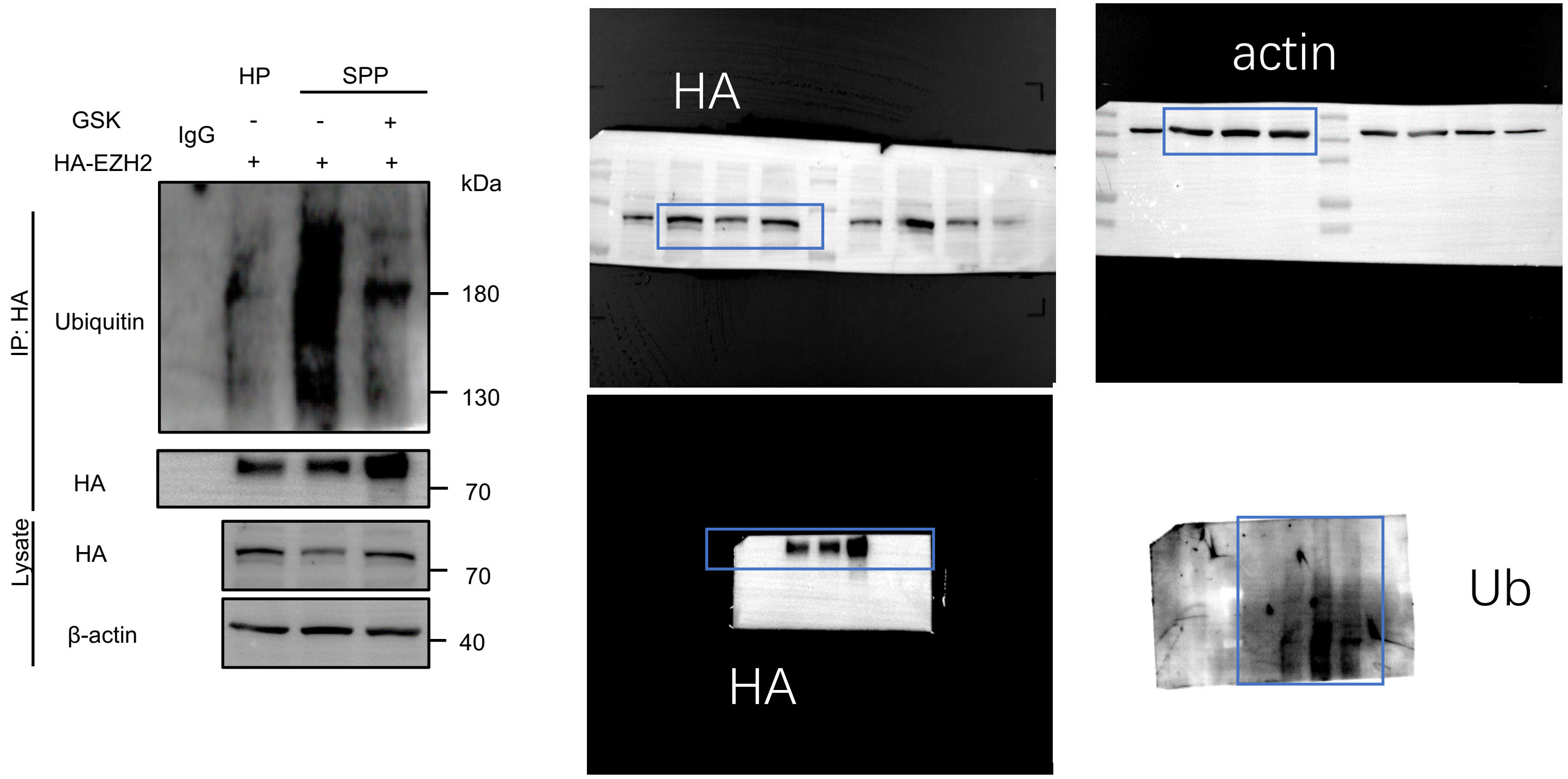
HP
SPP
GSK
IgG
HA-EZH2
kDa
IP: HA
Ubiquitin
180
130
HA
70
Lysate
HA
70
β-actin
40
HA
actin
HA
Ub

4G

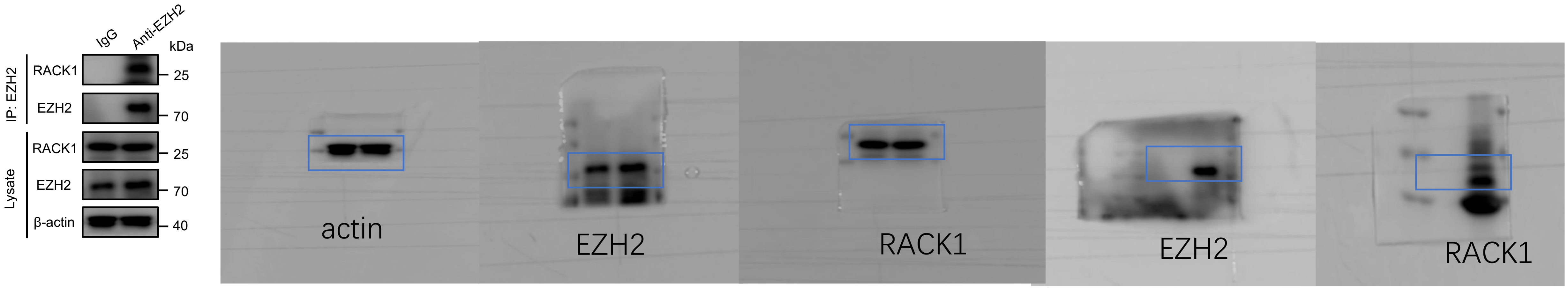
IgG
Anti-EZH2
kDa
IP: EZH2
RACK1
25
EZH2
70
Lysate
RACK1
25
EZH2
70
β-actin
40
actin
EZH2
RACK1
EZH2
RACK1

4H

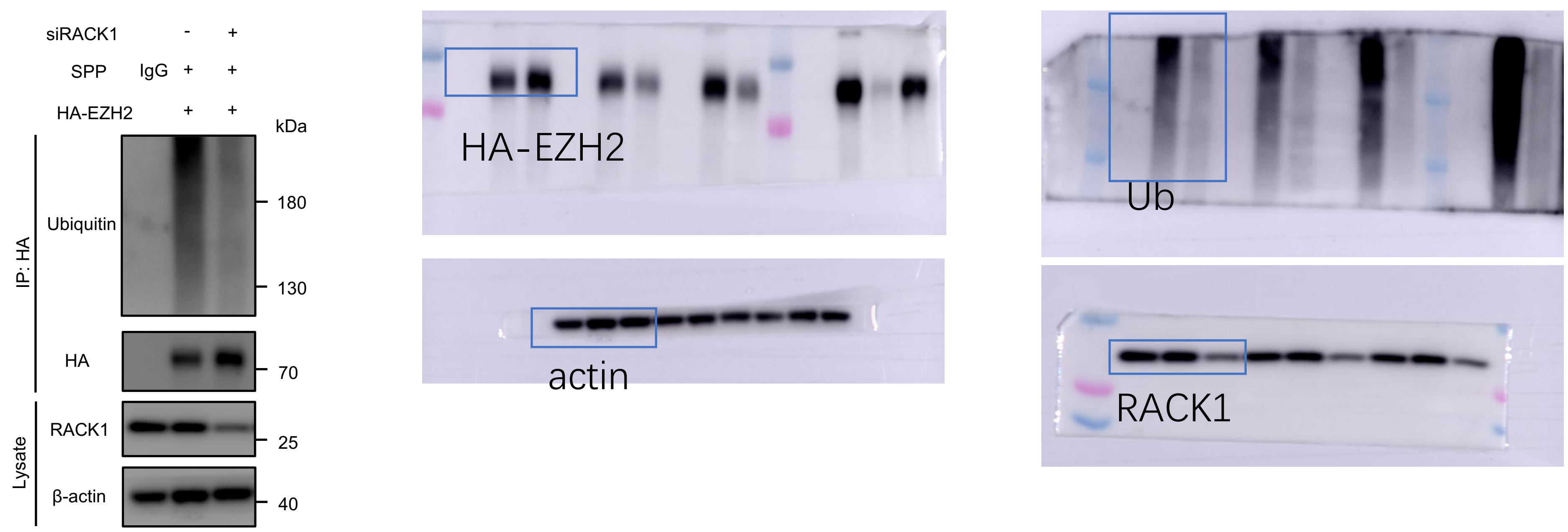
siRACK1 - +
SPP IgG + +
HA-EZH2 + +
kDa
IP: HA
Ubiquitin
180
130
HA
70
Lysate
RACK1
25
β-actin
40
HA-EZH2
actin
Ub
RACK1

4I

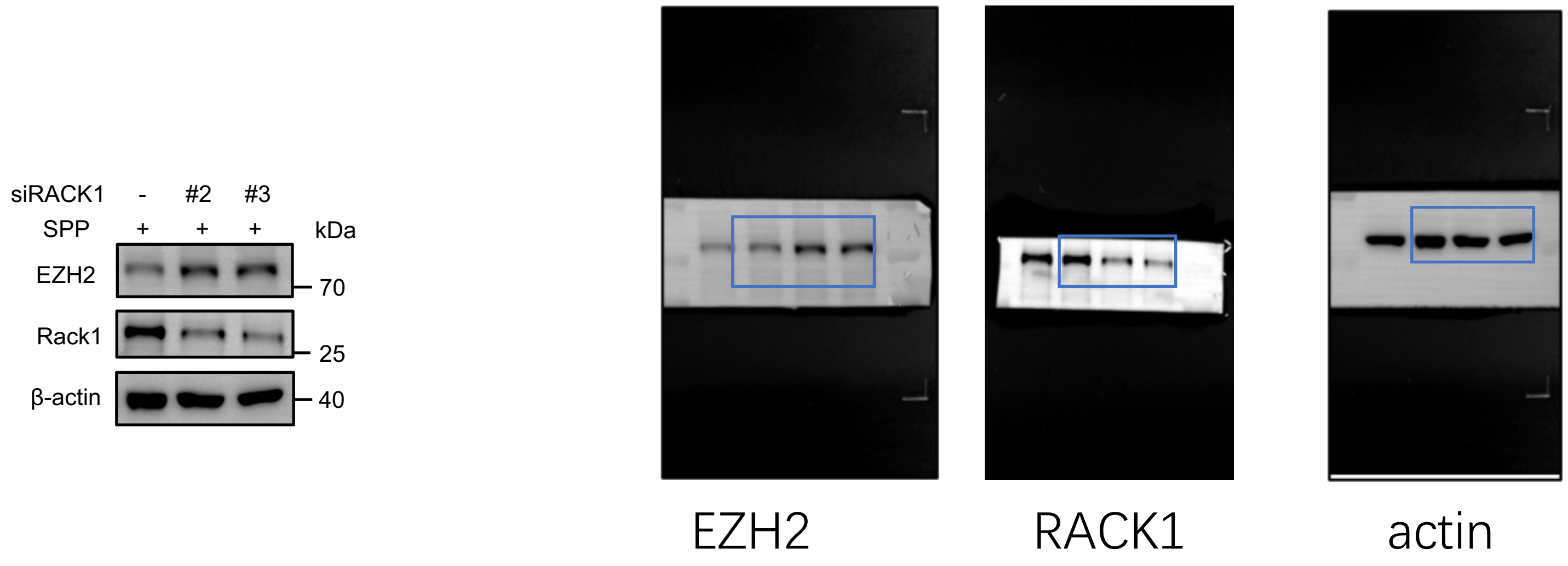
siRACK1 - #2 #3
SPP + + +
kDa
EZH2
70
Rack1
25
β-actin
40
EZH2
RACK1
actin

4J

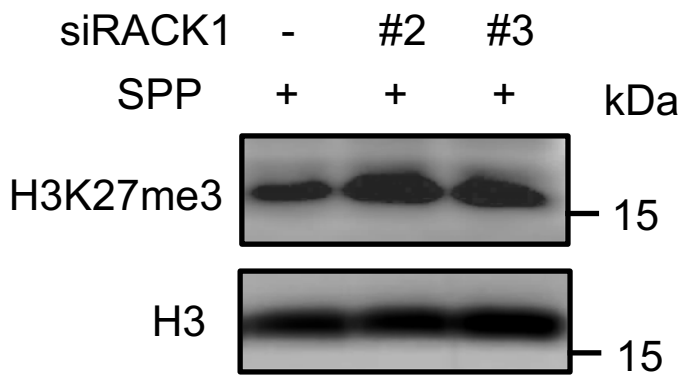
siRACK1 - #2 #3
SPP + + +
kDa
H3K27me3
15
H3
15

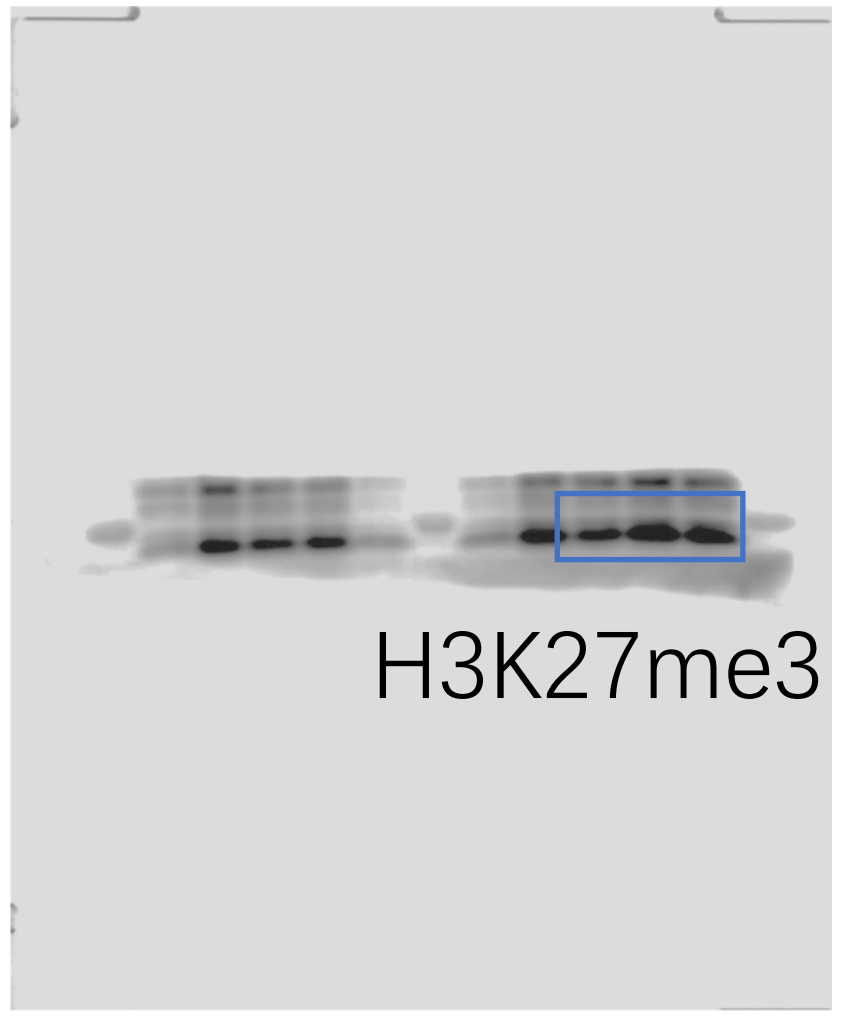
H3K27me3

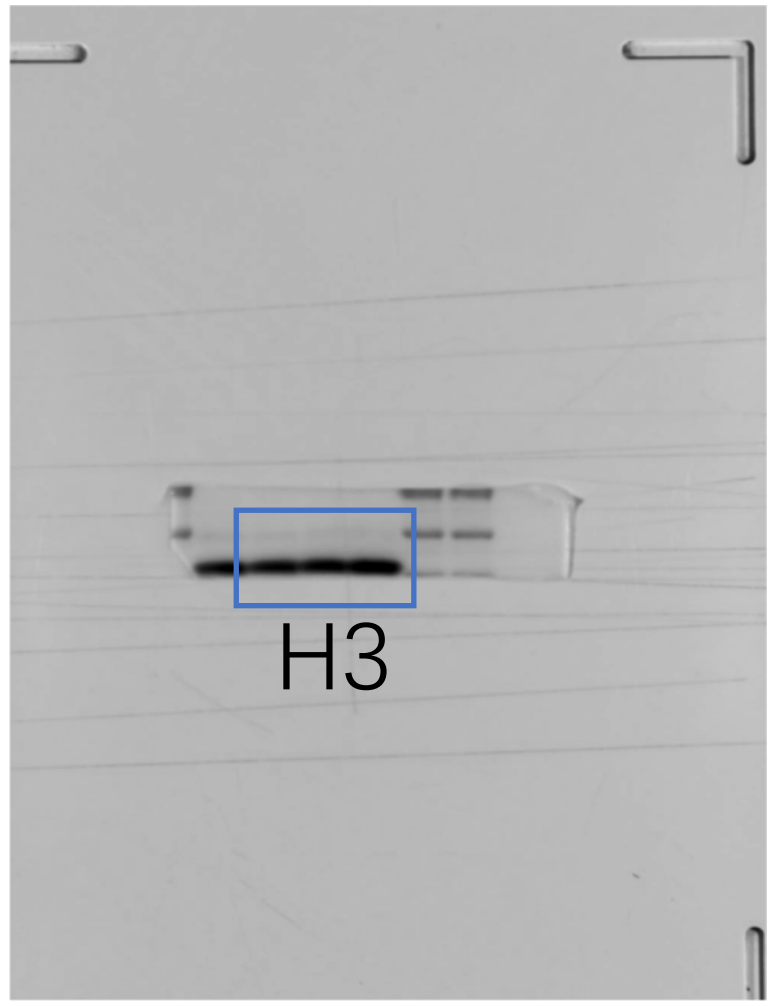
H3

4L

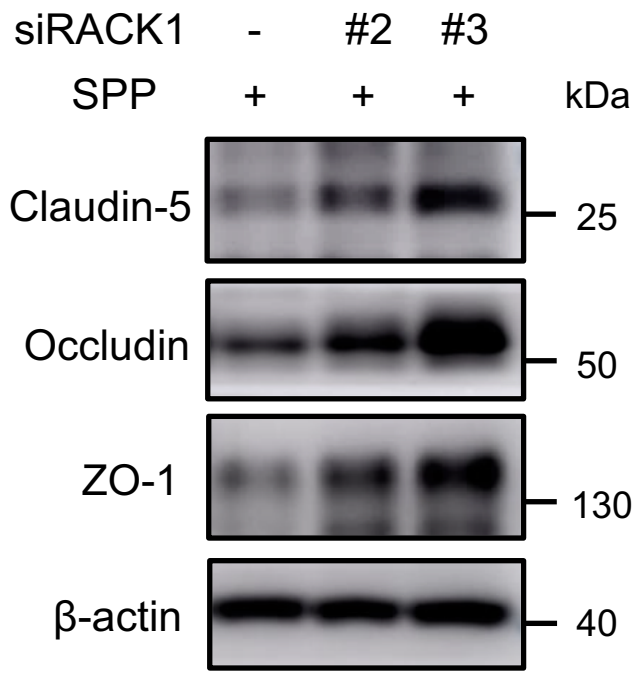
siRACK1 - #2 #3
SPP + + +
kDa
Claudin-5
25
Occludin
50
ZO-1
130
β-actin
40

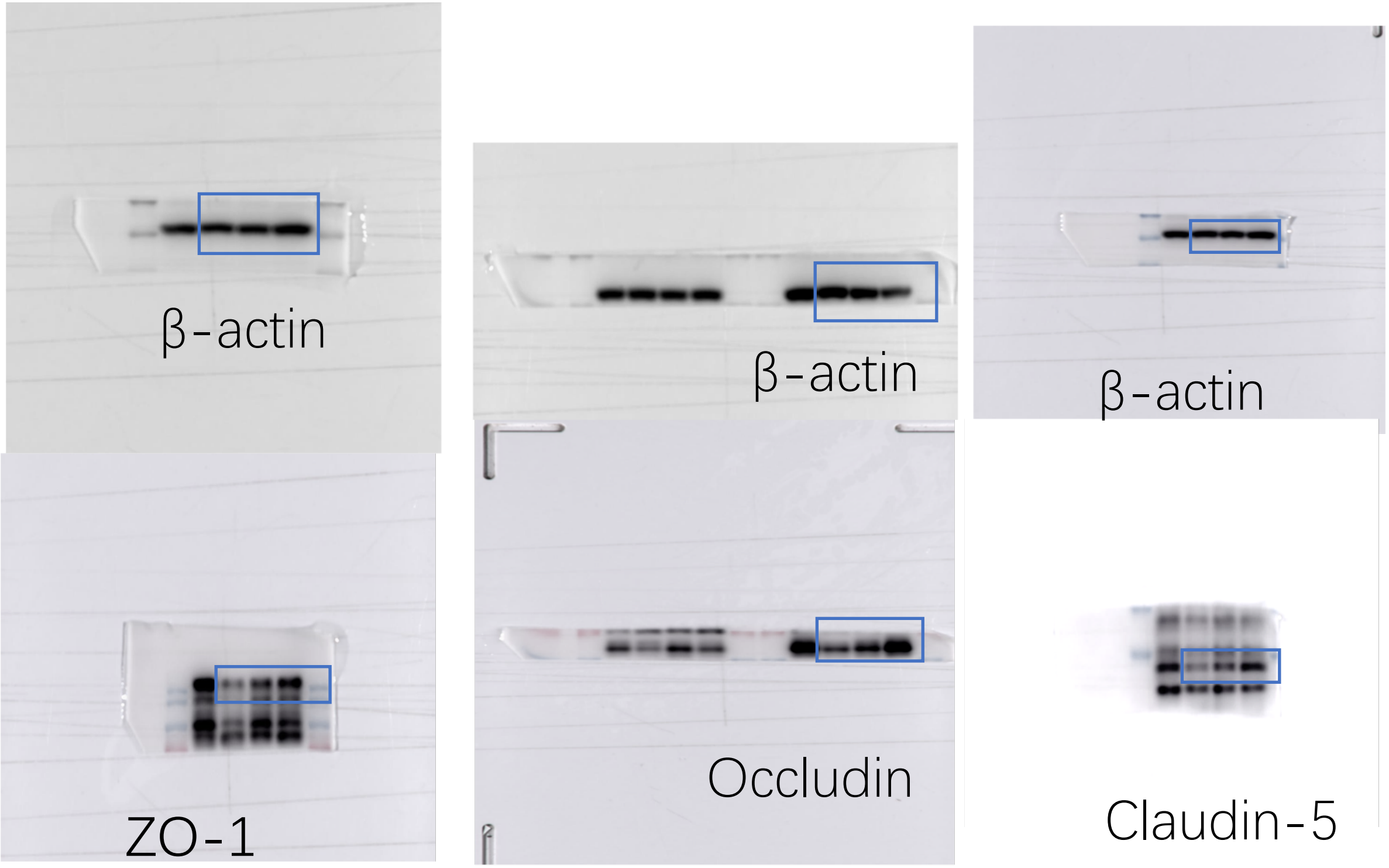
β-actin
β-actin
β-actin
ZO-1
Occludin
Claudin-5

4M

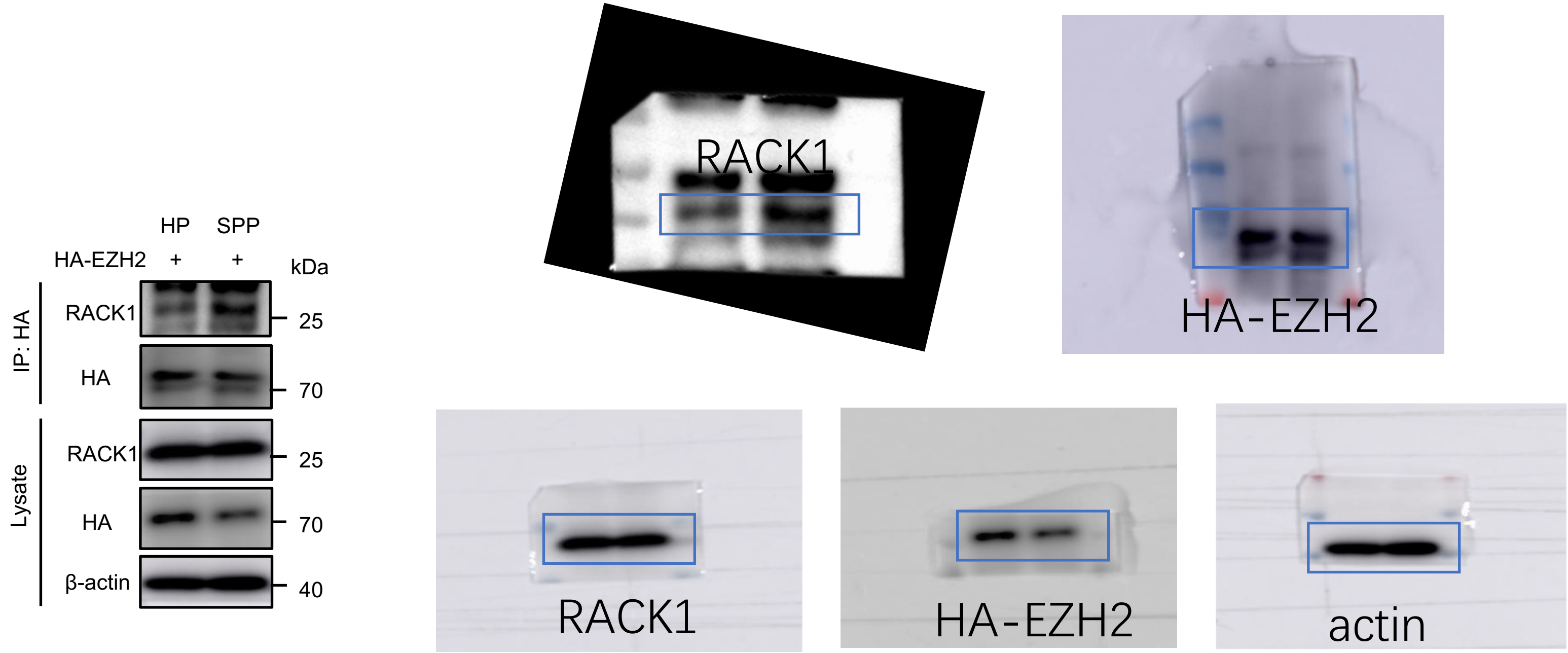
HP
SPP
HA-EZH2
+
+
kDa
IP: HA
RACK1
25
HA
70
Lysate
RACK1
25
HA
70
β-actin
40
RACK1
HA-EZH2
RACK1
HA-EZH2
actin

4N

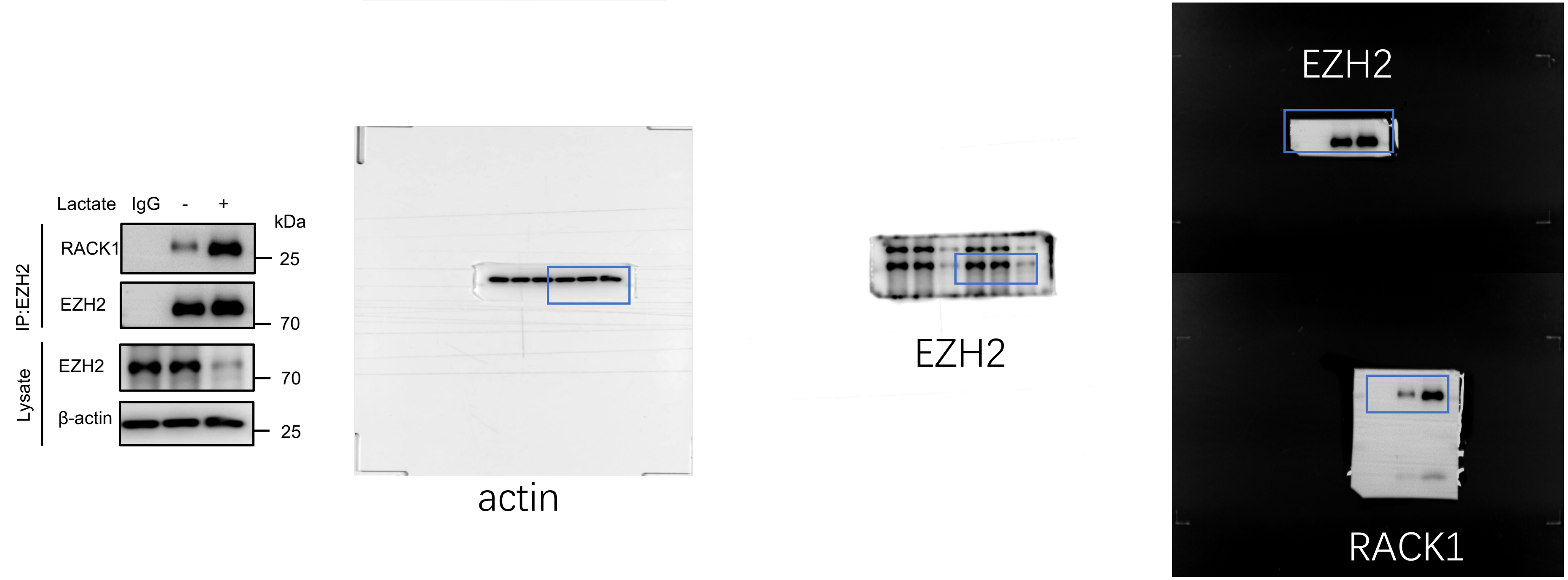
Lactate
IgG
-
+
kDa
IP:EZH2
RACK1
25
EZH2
70
Lysate
EZH2
70
β-actin
25
actin
EZH2
EZH2
RACK1

5A

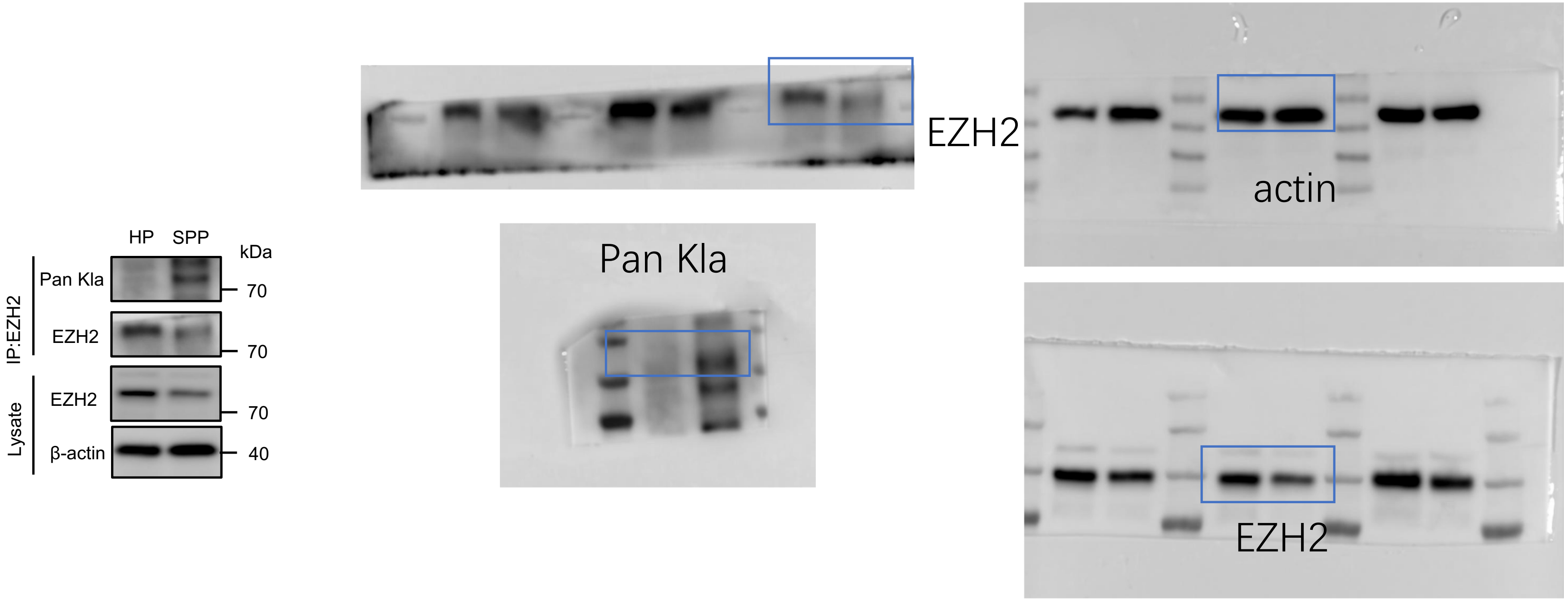
HP
SPP
kDa
IP:EZH2
Pan Kla
70
EZH2
70
Lysate
EZH2
70
β-actin
40
EZH2
actin
Pan Kla
EZH2

5B

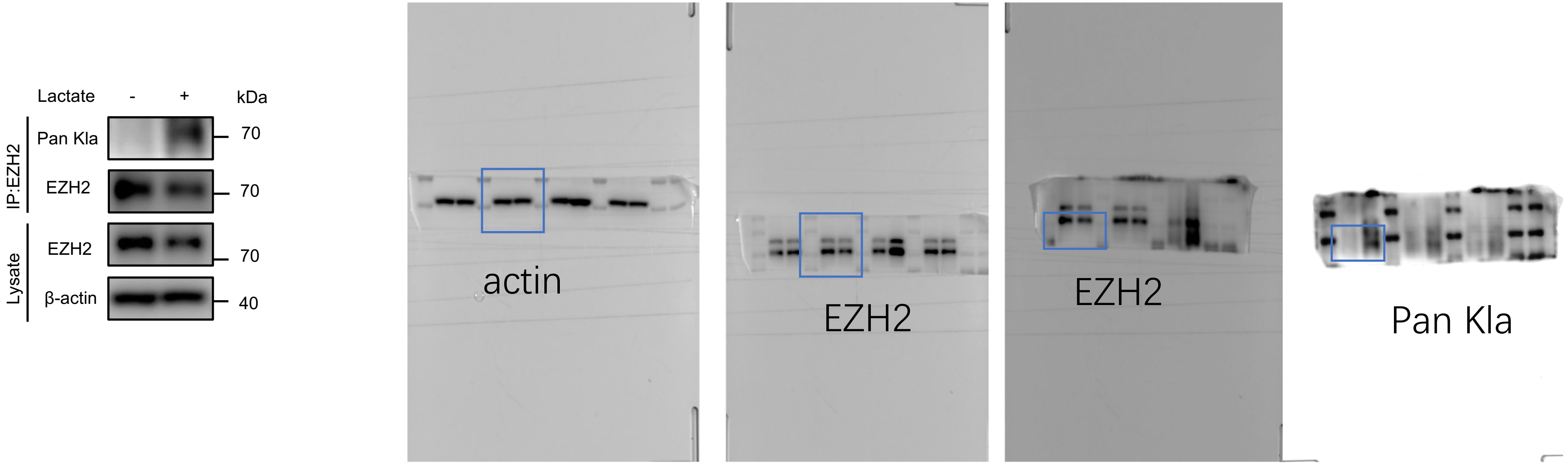
Lactate
-
+
kDa
IP:EZH2
Pan Kla
70
EZH2
70
Lysate
EZH2
70
β-actin
40
actin
EZH2
EZH2
Pan Kla

5C

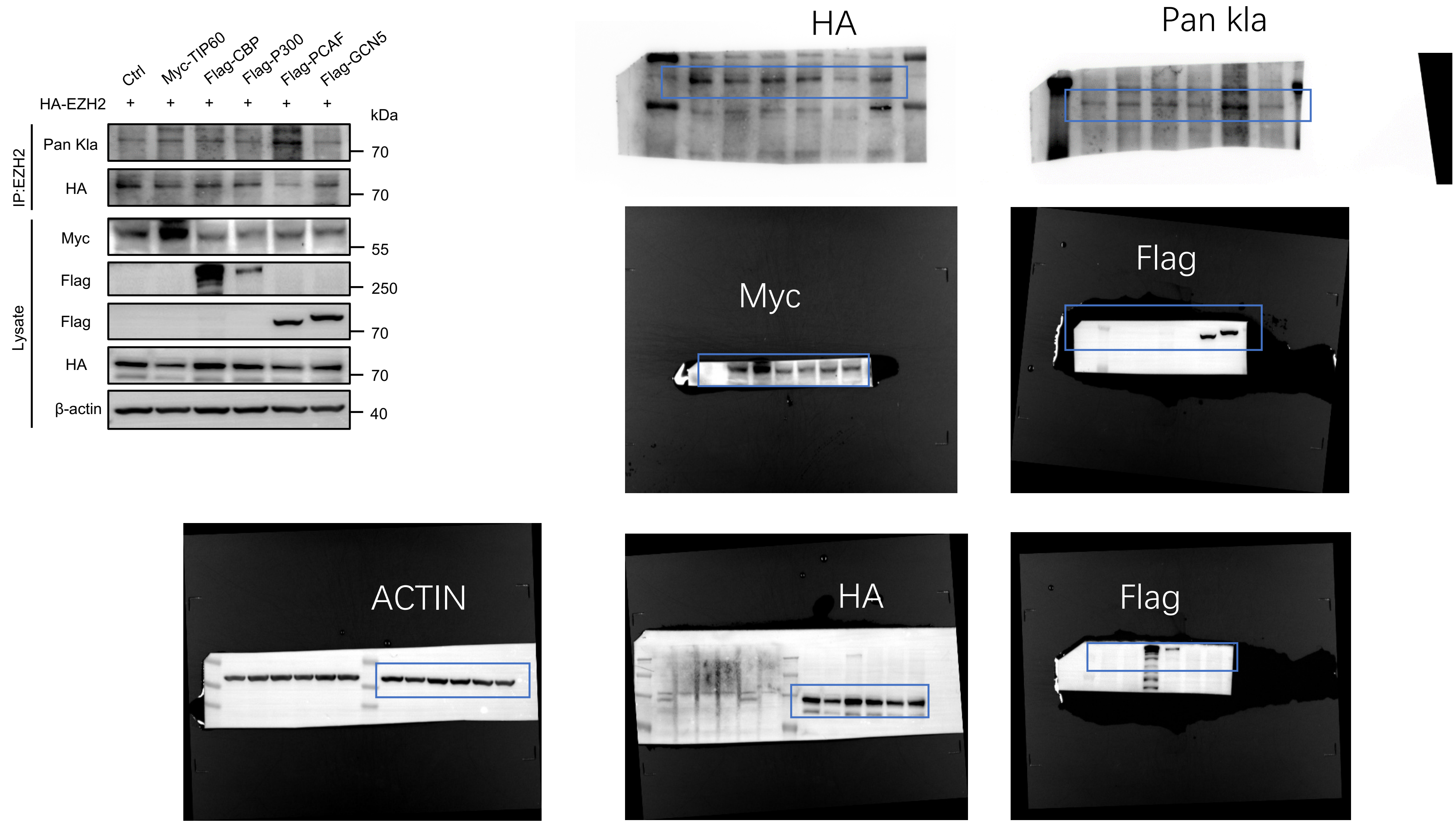

Ctrl
Myc-TIP60
Flag-CBP
Flag-P300
Flag-PCAF
Flag-GCN5
HA-EZH2
+ + + + + +
kDa
IP:EZH2
Pan Kla
70
HA
70
Lysate
Myc
55
Flag
250
Flag
70
HA
70
β-actin
40
HA
Pan kla
Myc
Flag
ACTIN
HA
Flag

5D

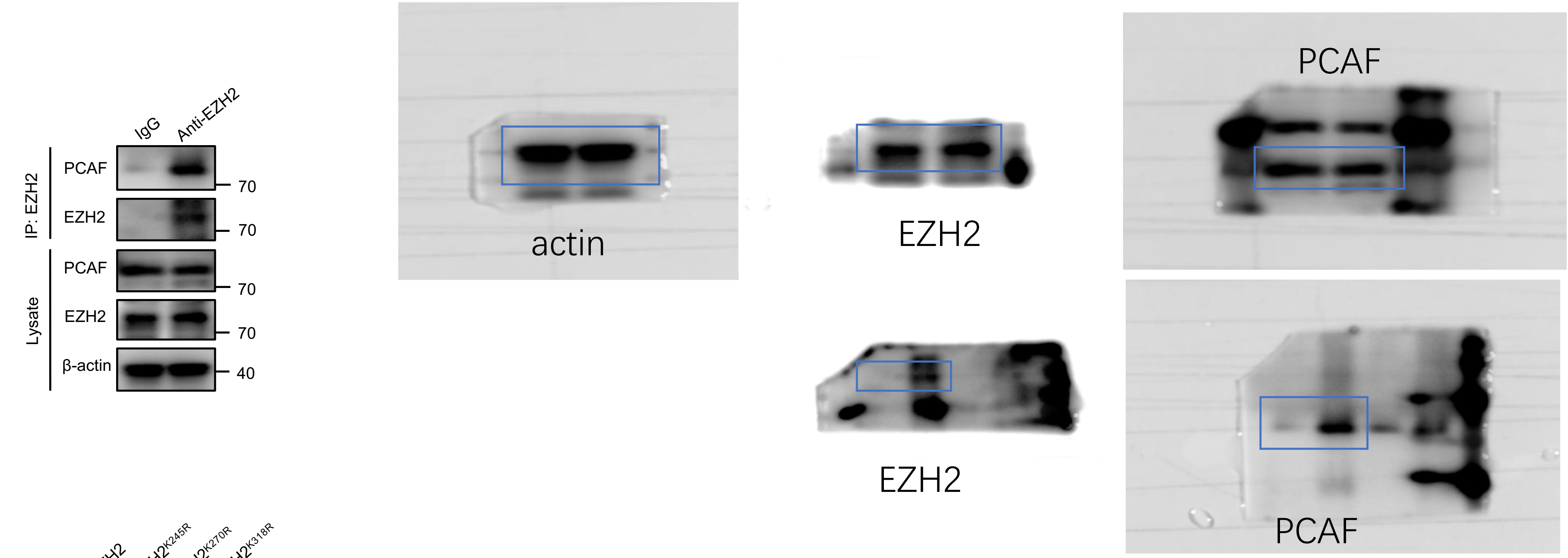

IgG
Anti-EZH2
IP: EZH2
PCAF
70
EZH2
70
Lysate
PCAF
70
EZH2
70
β-actin
40
actin
EZH2
PCAF
EZH2
PCAF

5E

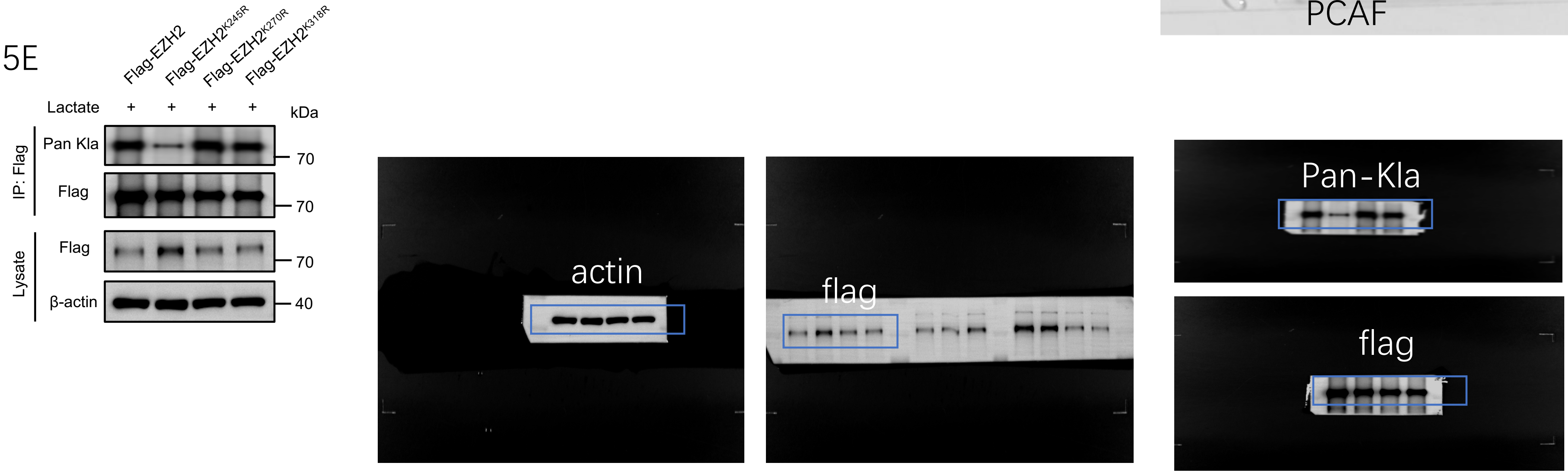

Flag-EZH2
Flag-EZH2K245R
Flag-EZH2K270R
Flag-EZH2K318R
Lactate
+
+
+
+
kDa
IP: Flag
Pan Kla
70
Flag
70
Lysate
Flag
70
β-actin
40
actin
flag
Pan-Kla
flag

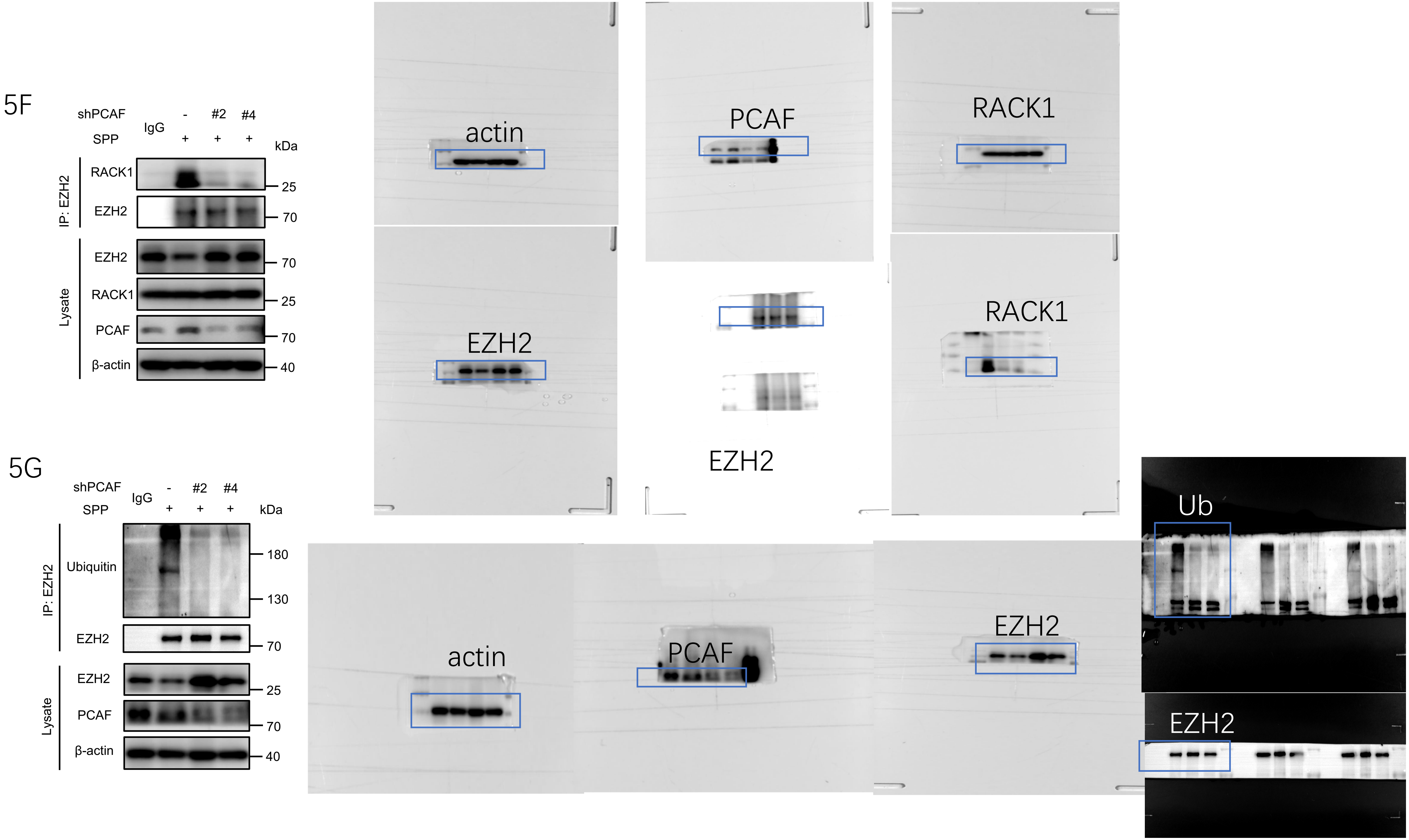
5F
shPCAF
SPP
IgG
-
#2
#4
+
+
+
kDa
IP: EZH2
RACK1
EZH2
Lysate
EZH2
RACK1
PCAF
β-actin
25
70
70
25
70
40
5G
shPCAF
SPP
IgG
-
#2
#4
+
+
+
kDa
IP: EZH2
Ubiquitin
EZH2
Lysate
EZH2
PCAF
β-actin
180
130
70
25
70
40
actin
PCAF
RACK1
EZH2
EZH2
RACK1
actin
PCAF
EZH2
Ub
EZH2

5H

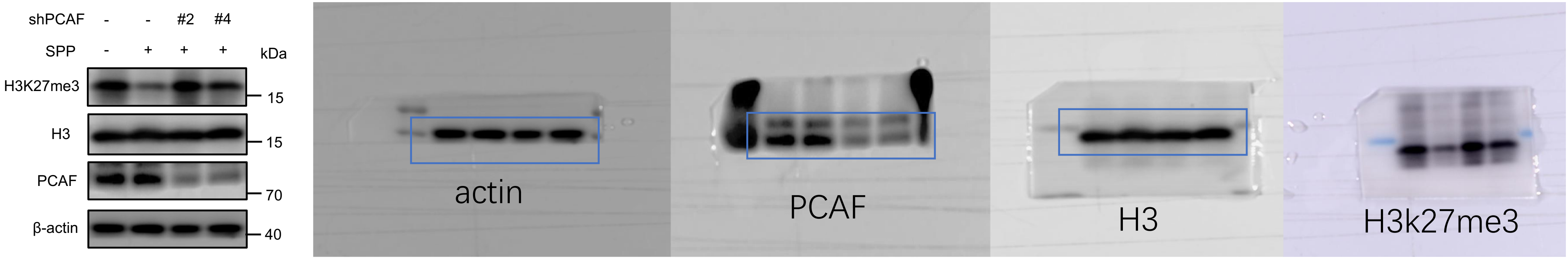
shPCAF - - #2 #4
SPP - + + +
kDa
H3K27me3
15
H3
15
PCAF
70
β-actin
40
actin
PCAF
H3
H3k27me3

5J

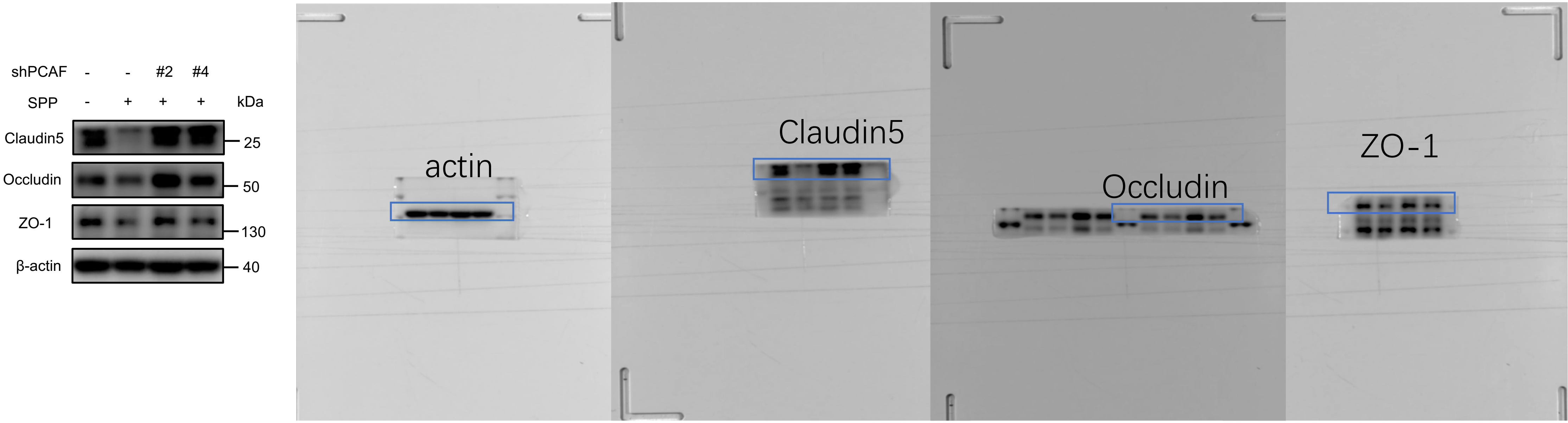
shPCAF - - #2 #4
SPP - + + +
kDa
Claudin5
25
Occludin
50
ZO-1
130
β-actin
40
actin
Claudin5
Occludin
ZO-1

5K

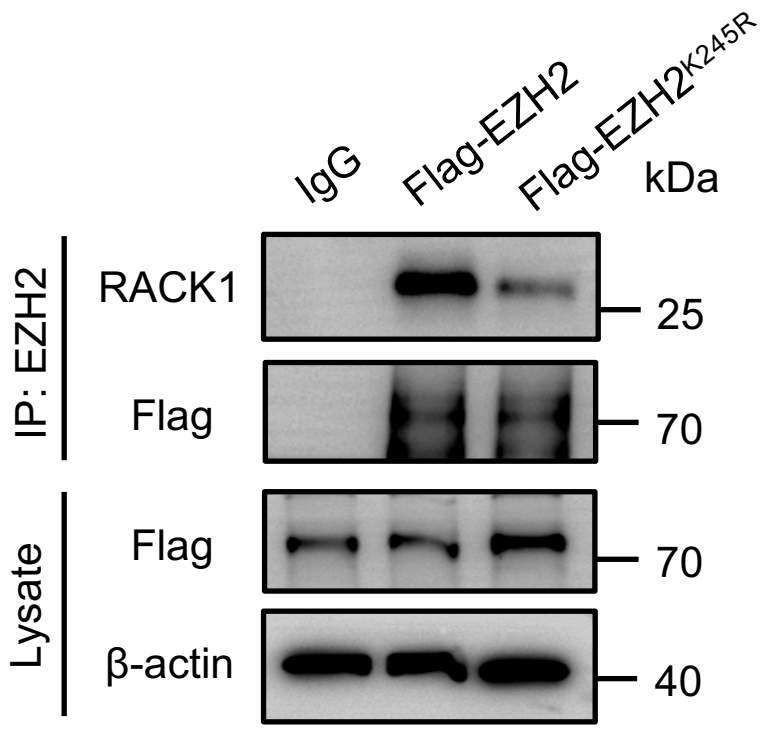
IgG
Flag-EZH2
Flag-EZH2K245R
kDa
IP: EZH2
RACK1
25
Flag
70
Lysate
Flag
70
β-actin
40

5L

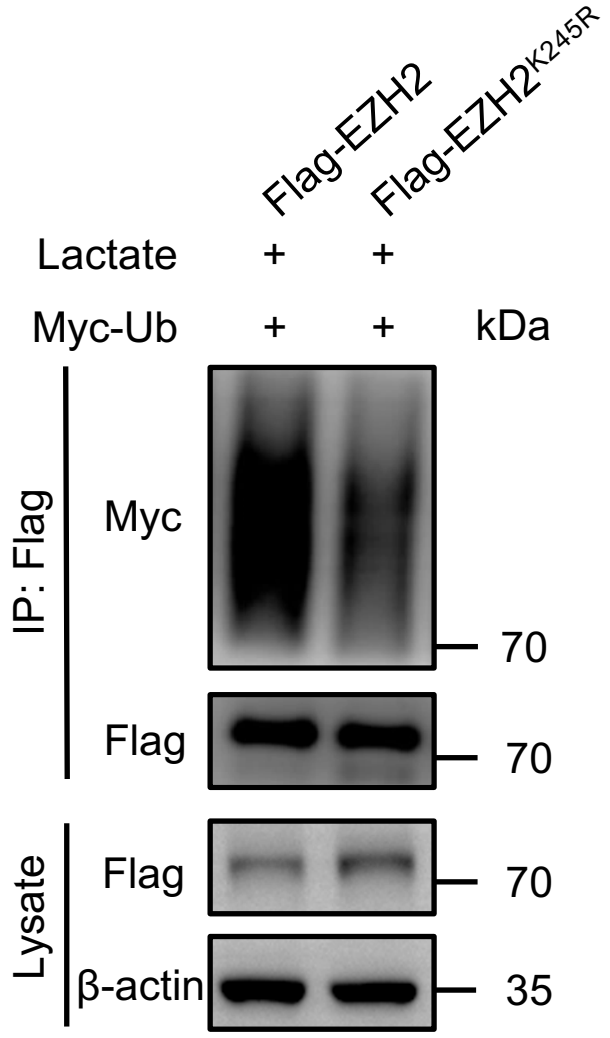
Flag-EZH2
Flag-EZH2K245R
Lactate + +
Myc-Ub + +
kDa
IP: Flag
Myc
70
Flag
70
Lysate
Flag
70
β-actin
35

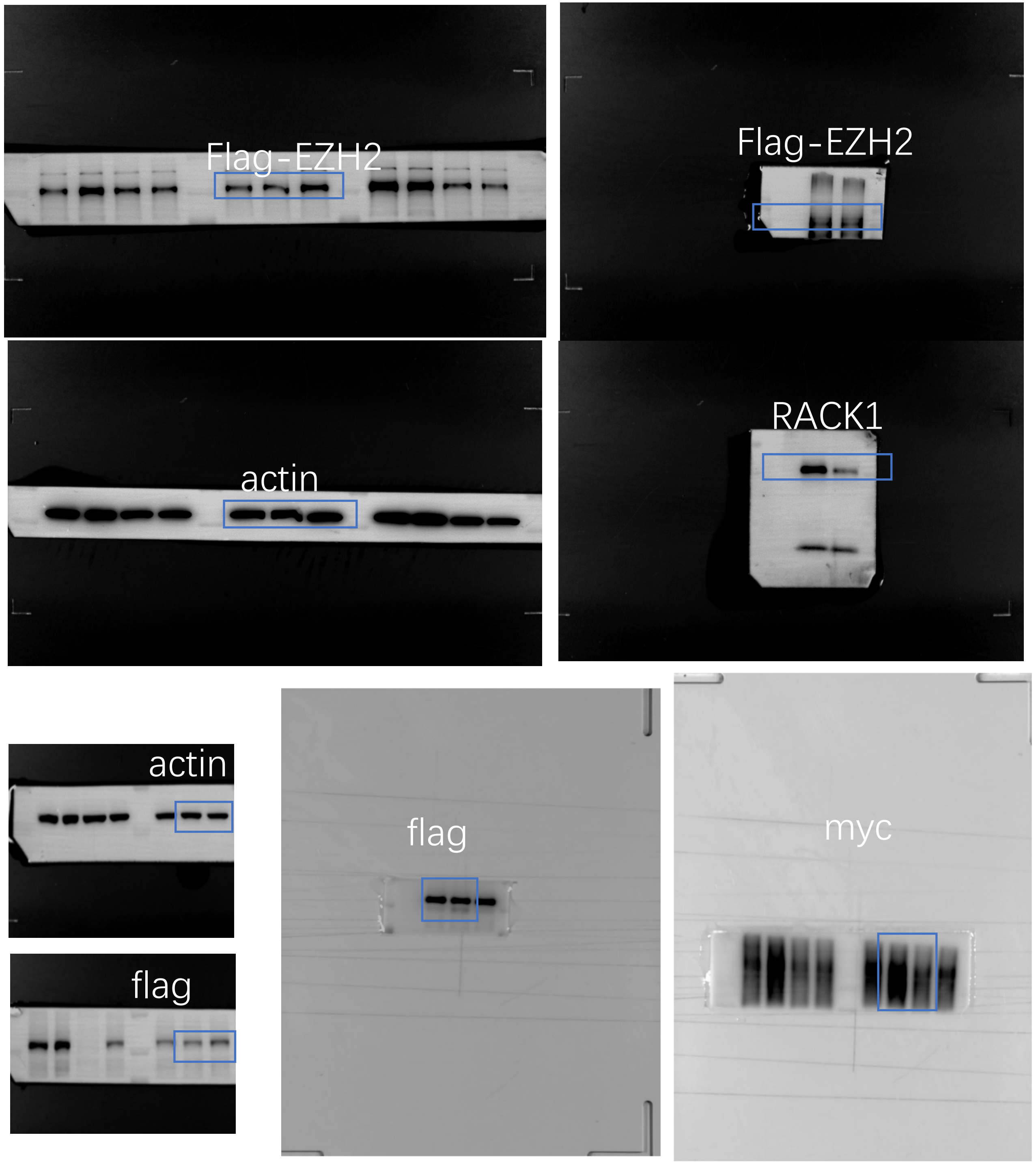
Flag-EZH2
Flag-EZH2
actin
RACK1
actin
flag
flag
myc

6B

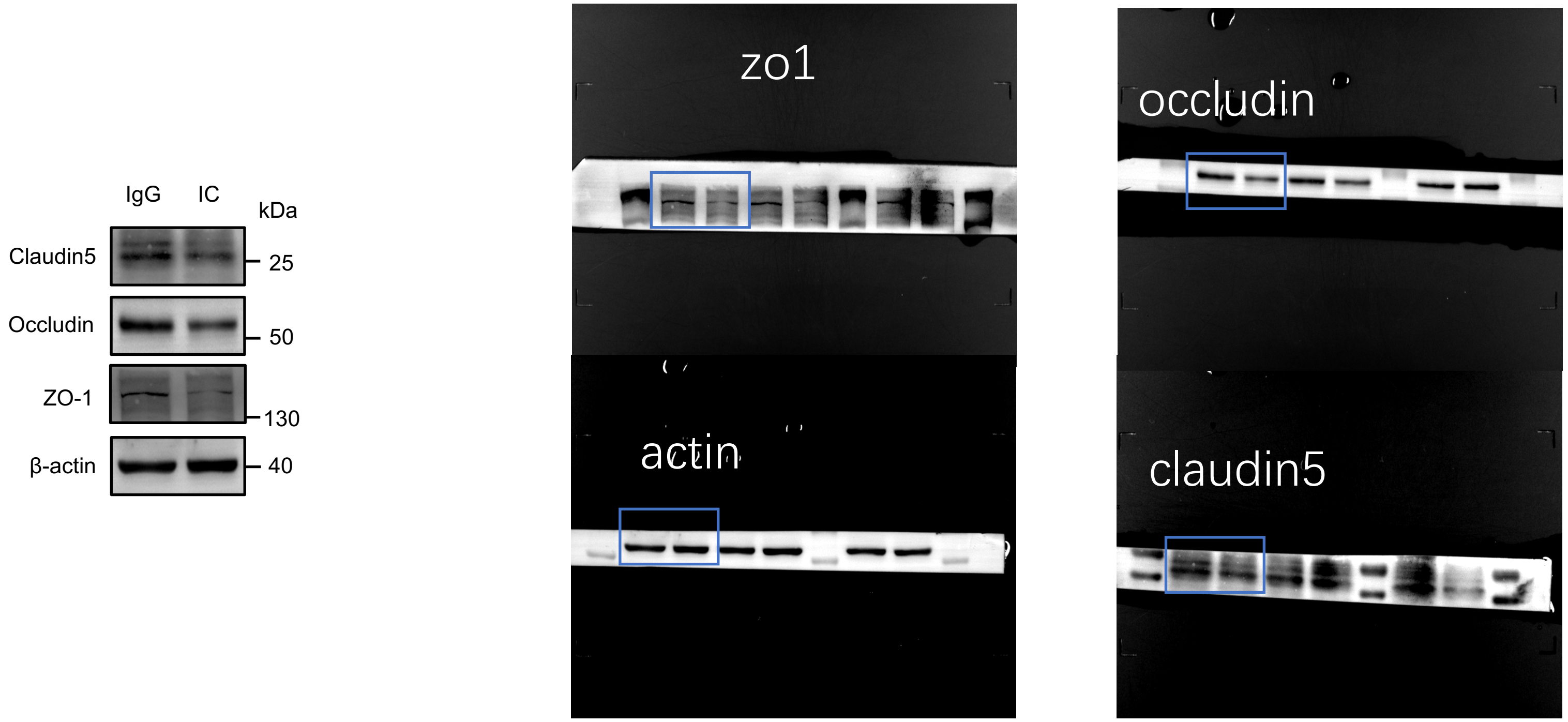
IgG
IC
kDa
Claudin5
25
Occludin
50
ZO-1
130
β-actin
40
zo1
occludin
actin
claudin5

6C

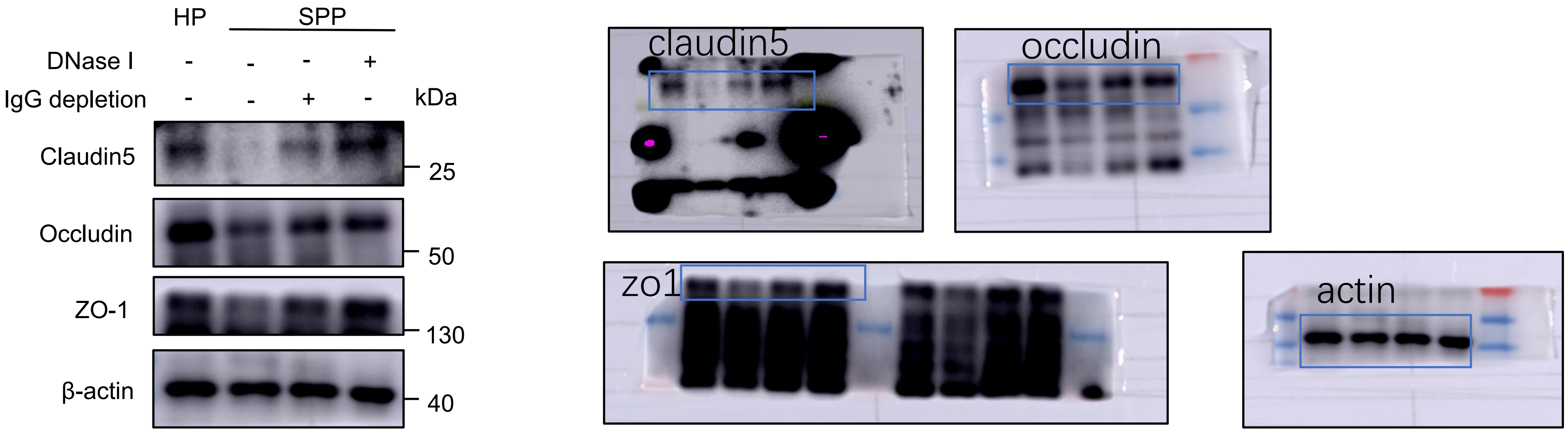
HP
SPP
DNase I
-
-
-
+
IgG depletion
-
-
+
-
kDa
Claudin5
25
Occludin
50
ZO-1
130
β-actin
40
claudin5
occludin
zo1
actin

6E

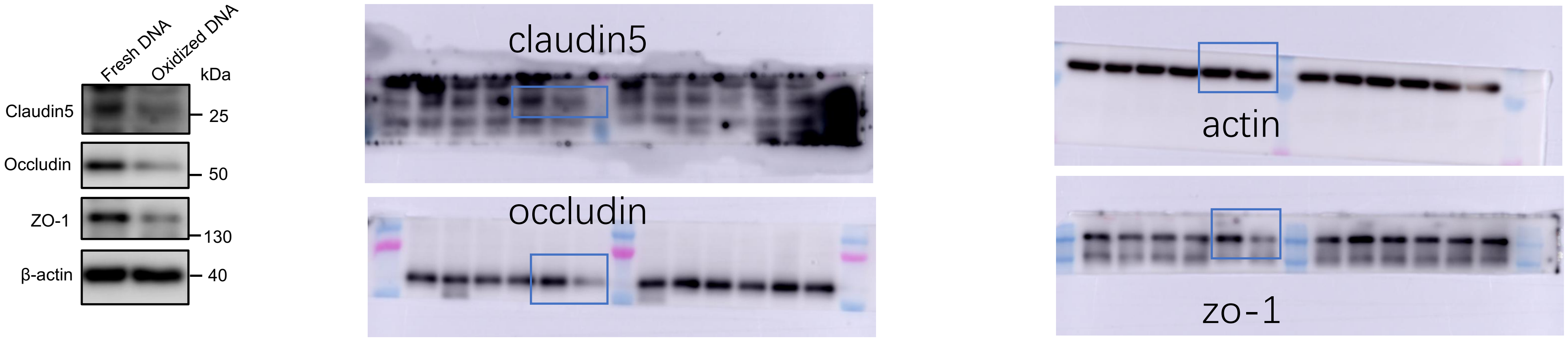
Fresh DNA
Oxidized DNA
kDa
Claudin5
25
Occludin
50
ZO-1
130
β-actin
40
claudin5
occludin
actin
zo-1

6I

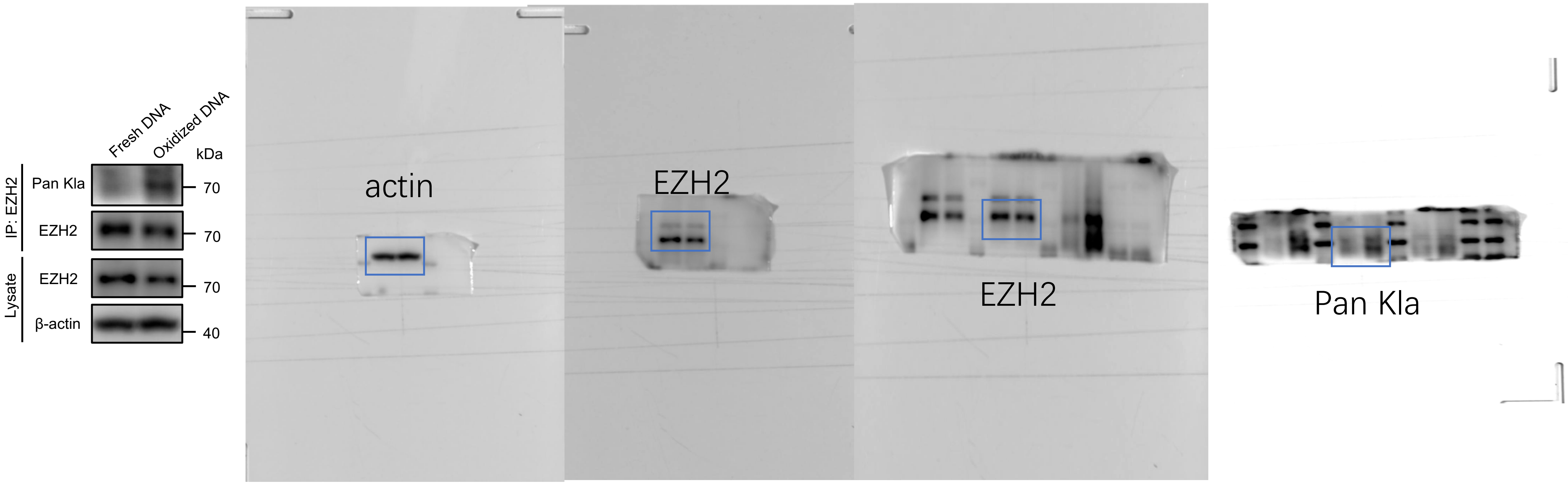
Fresh DNA
Oxidized DNA
kDa
IP: EZH2
Pan Kla
70
EZH2
70
Lysate
EZH2
70
β-actin
40
actin
EZH2
EZH2
Pan Kla

6J

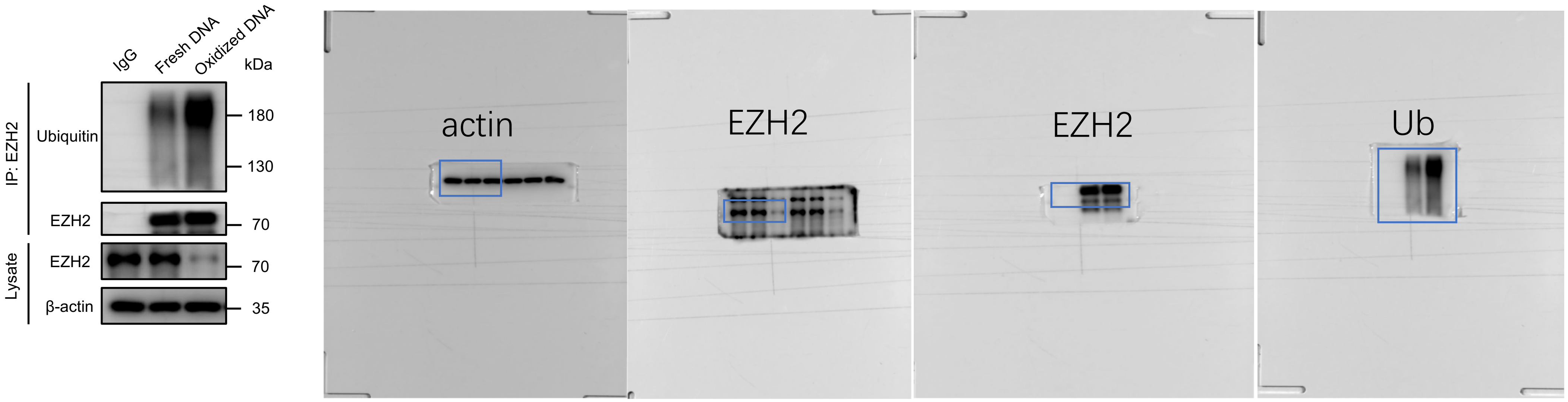
IgG
Fresh DNA
Oxidized DNA
kDa
IP: EZH2
Ubiquitin
180
130
EZH2
70
Lysate
EZH2
70
β-actin
35
actin
EZH2
EZH2
Ub

6N

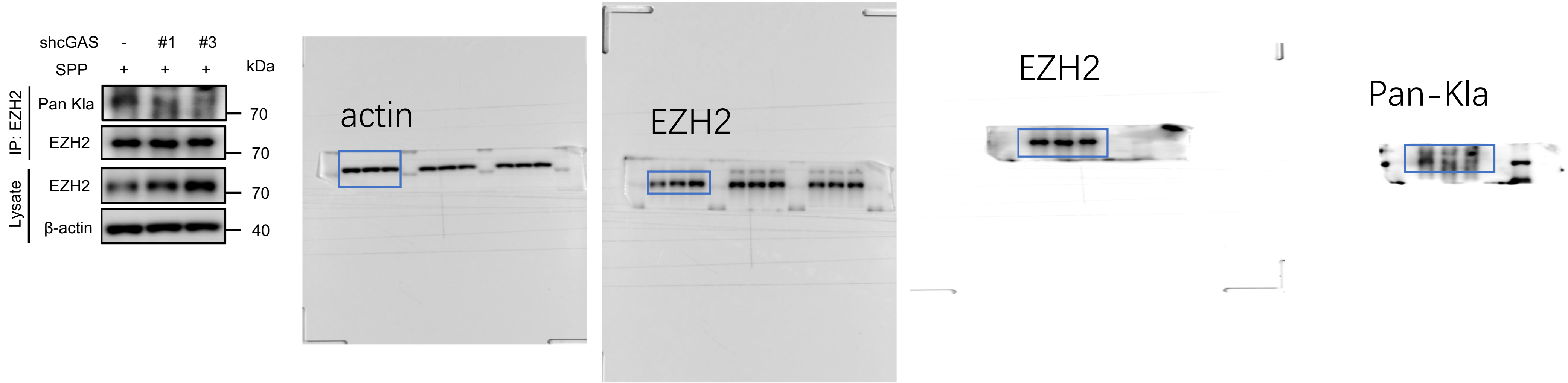
shcGAS - #1 #3
SPP + + +
kDa
IP: EZH2
Pan Kla
70
EZH2
70
Lysate
EZH2
70
β-actin
40
actin
EZH2
EZH2
Pan-Kla

6O

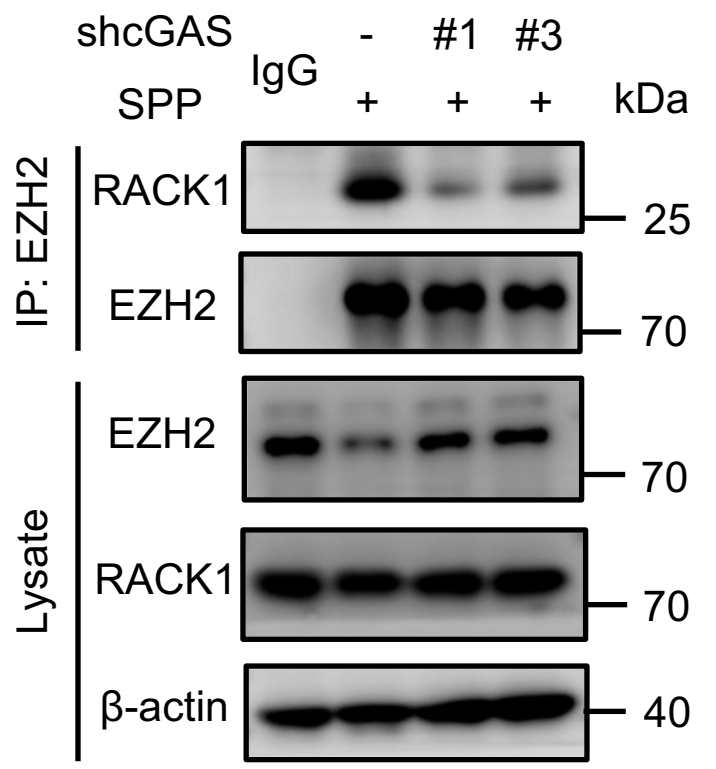

shcGAS
SPP
IgG
-
#1
#3
+
+
+
kDa
IP: EZH2
RACK1
25
EZH2
70
Lysate
EZH2
70
RACK1
70
β-actin
40

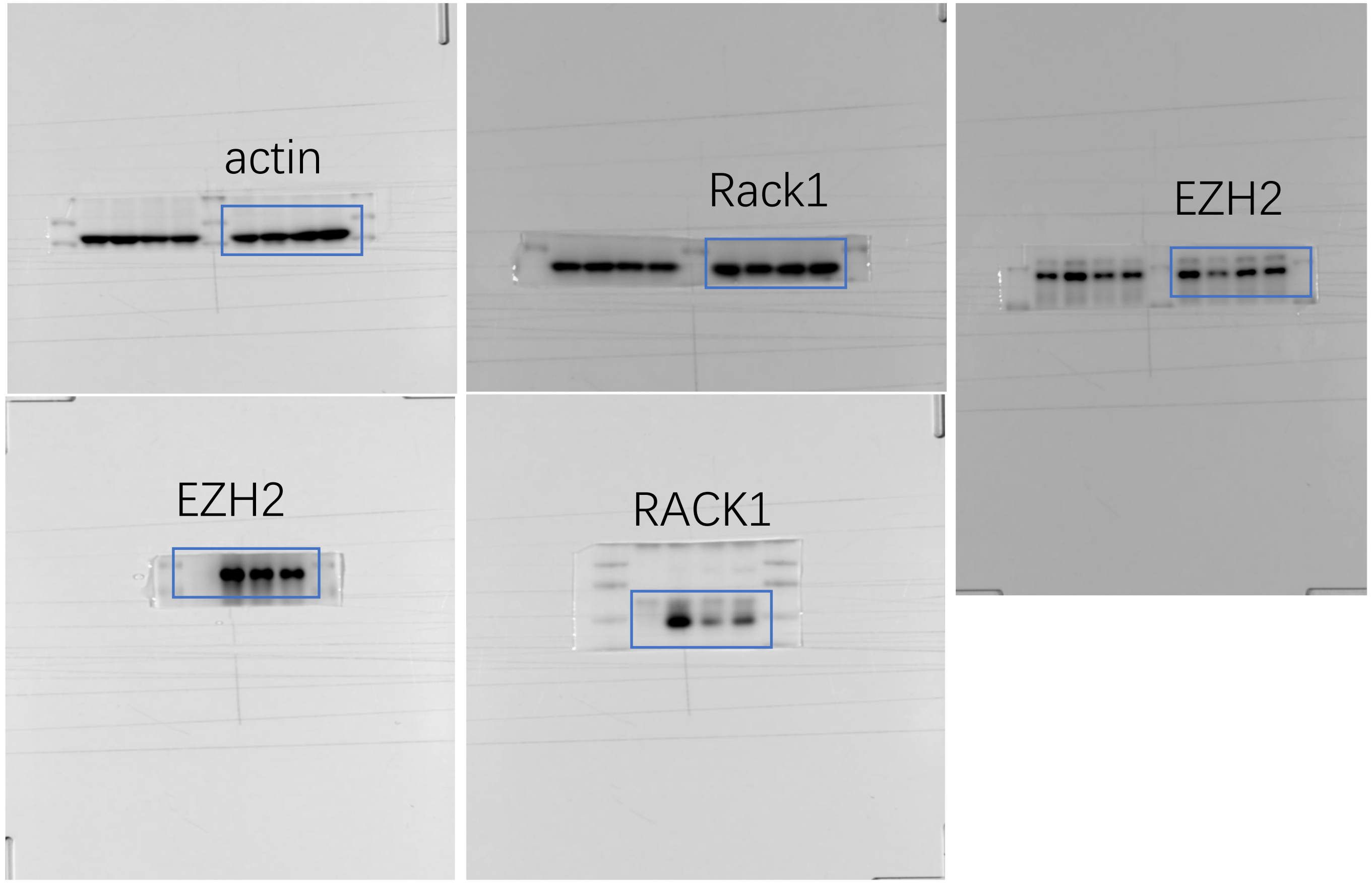

actin
Rack1
EZH2
EZH2
RACK1

6P

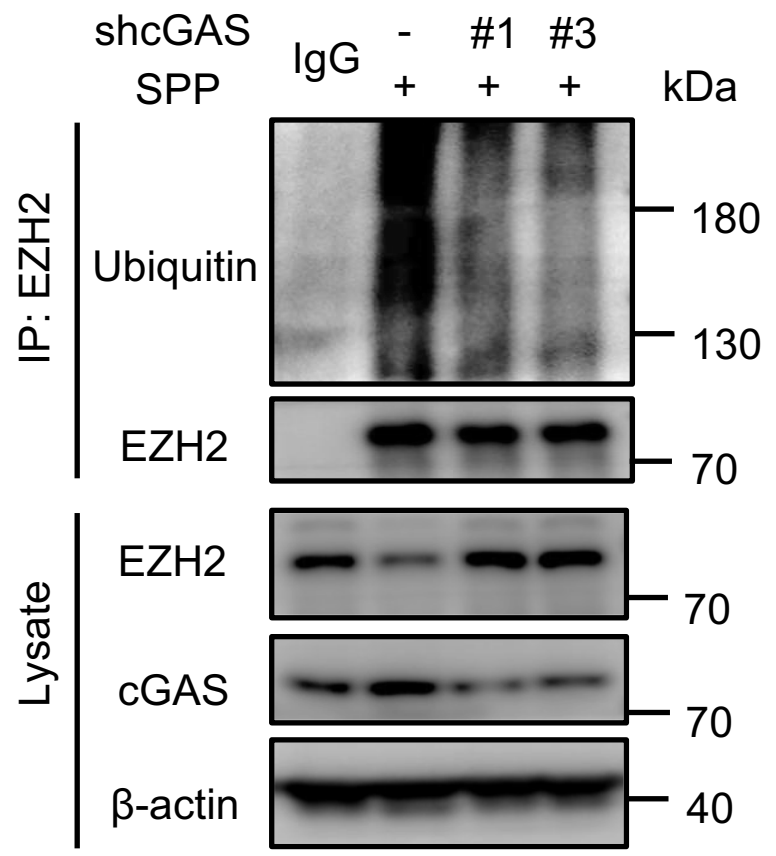

shcGAS
SPP
IgG
-
#1
#3
+
+
+
kDa
IP: EZH2
Ubiquitin
180
130
EZH2
70
Lysate
EZH2
70
cGAS
70
β-actin
40

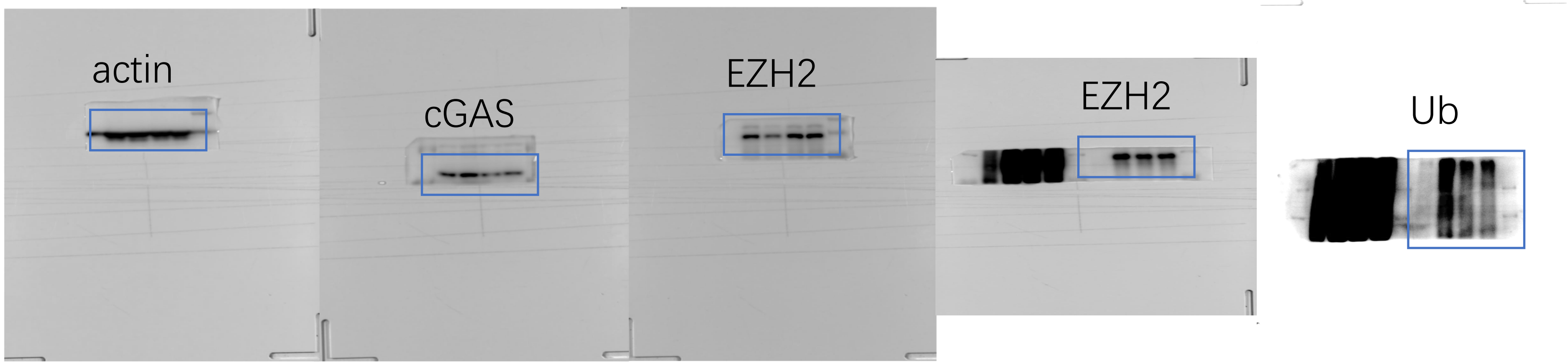

actin
cGAS
EZH2
EZH2
Ub

6Q

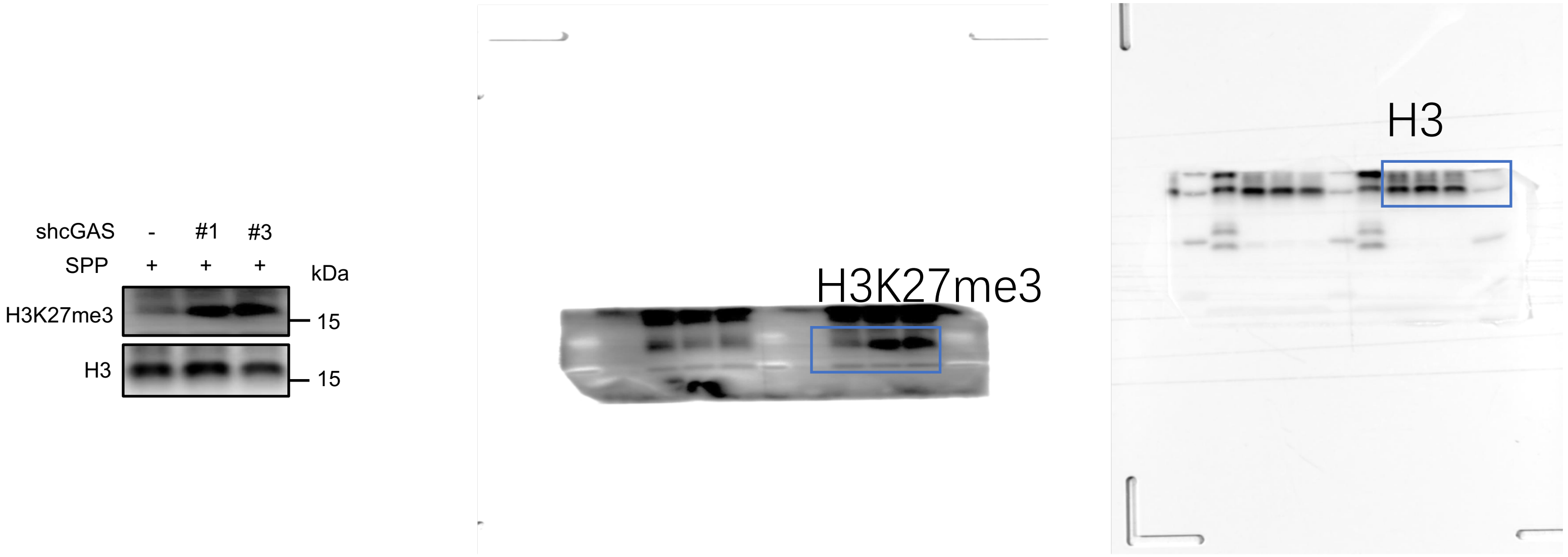
shcGAS - #1 #3
SPP + + +
kDa
H3K27me3
H3
15
15
H3K27me3
H3

6S

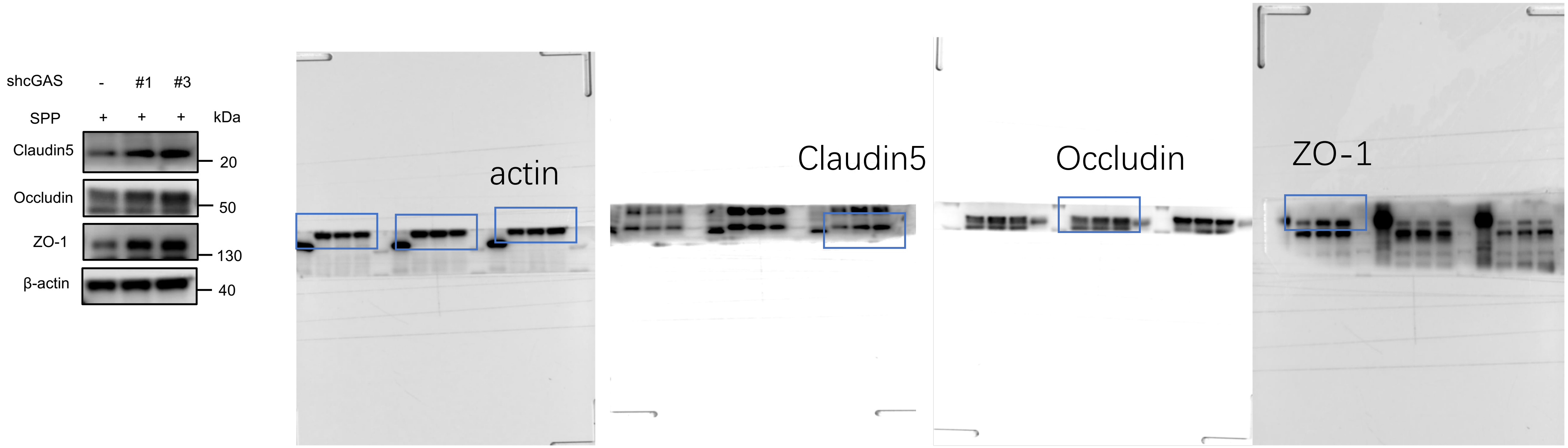
shcGAS - #1 #3
SPP + + +
kDa
Claudin5
Occludin
ZO-1
β-actin
20
50
130
40
actin
Claudin5
Occludin
ZO-1

S1E

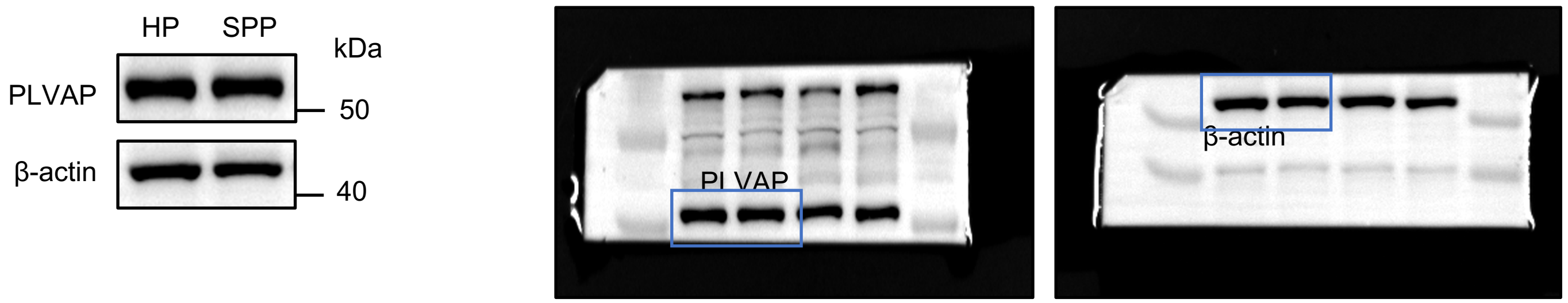

S1G

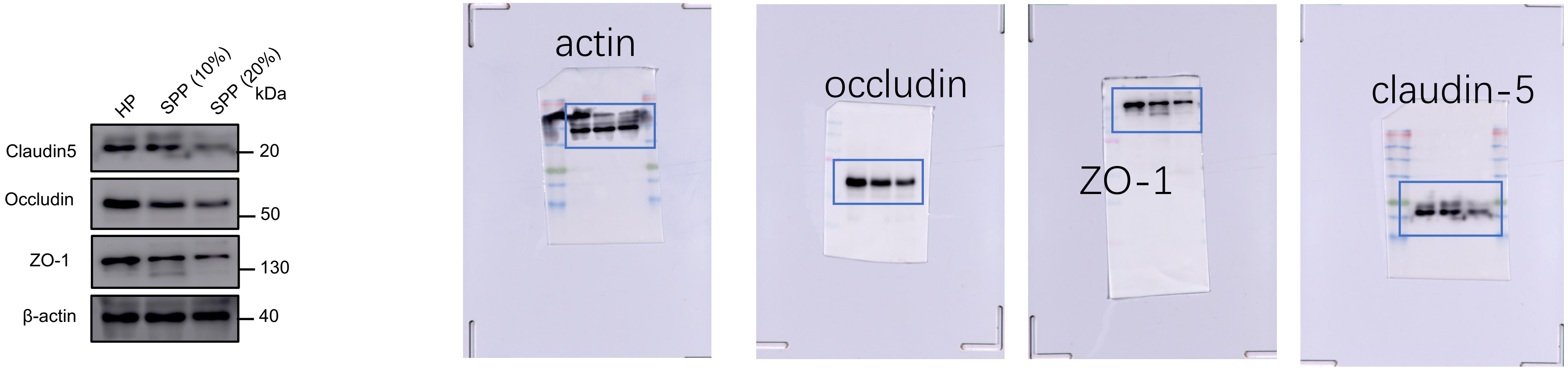
HP
SPP (10%)
SPP (20%)
kDa
Claudin5
Occludin
ZO-1
β-actin
20
50
130
40
actin
occludin
ZO-1
claudin-5

S5D

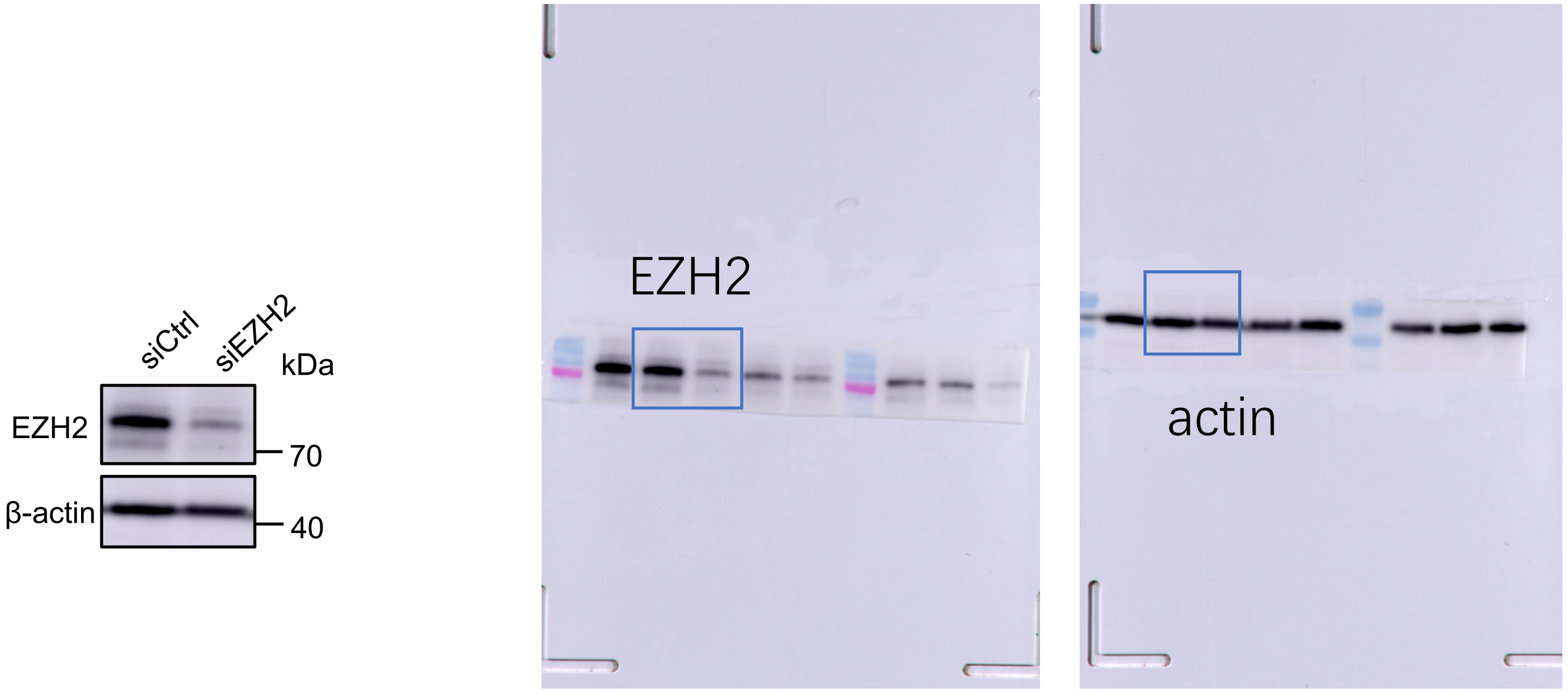
siCtrl
siEZH2
kDa
EZH2
β-actin
70
40
EZH2
actin

S5F

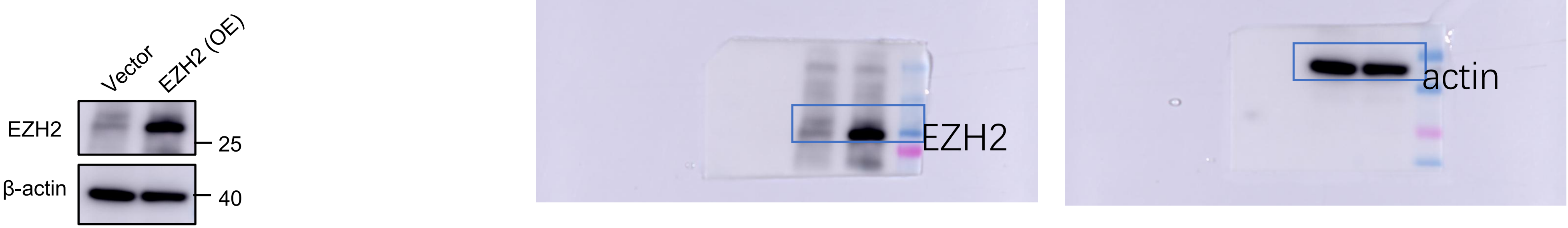

Vector
EZH2 (OE)
EZH2
β-actin
25
40
EZH2
actin

S6F

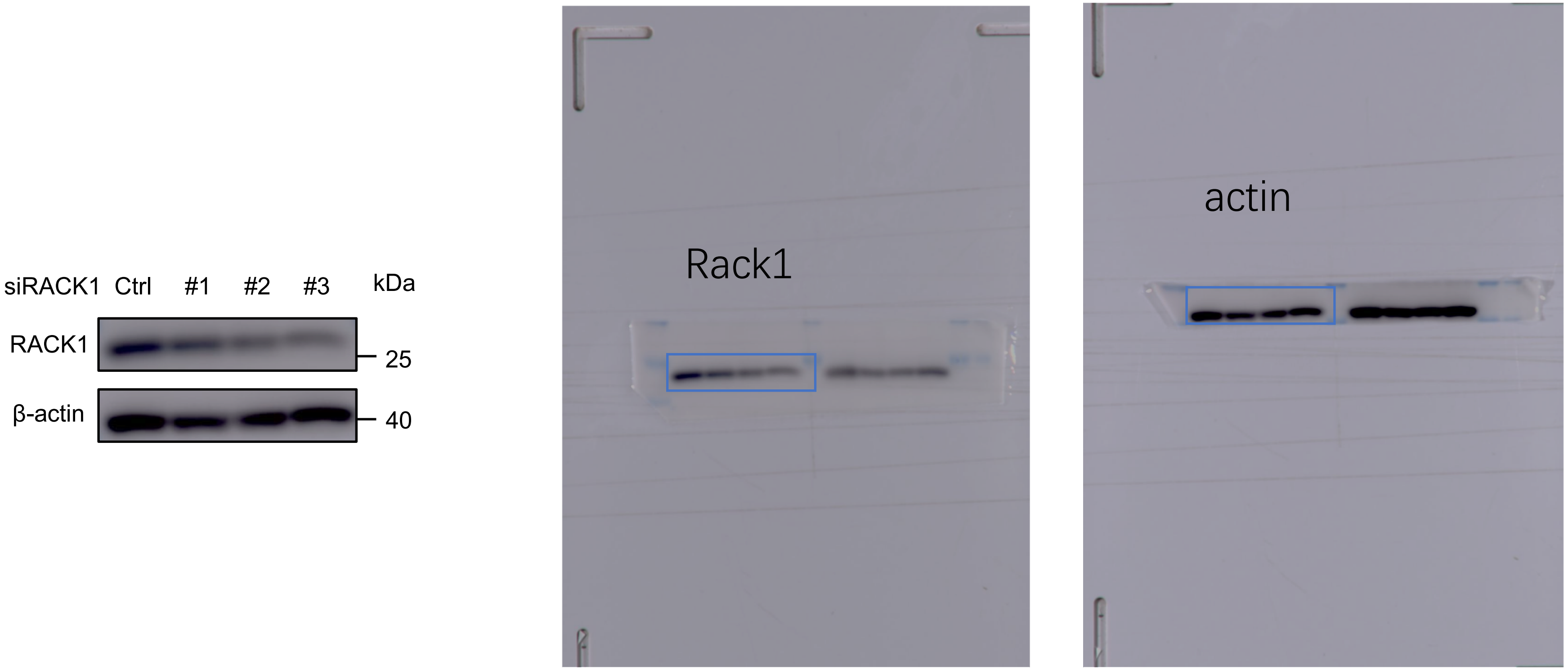

siRACK1
Ctrl
#1
#2
#3
kDa
RACK1
β-actin
25
40
Rack1
actin

S6G

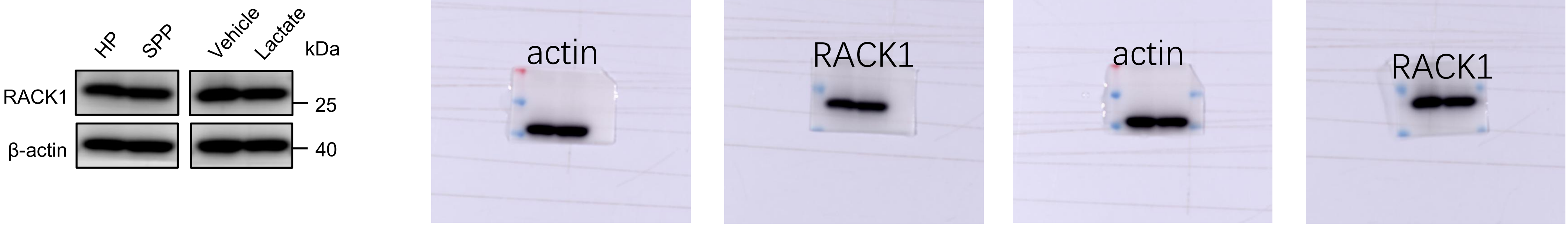
HP
SPP
Vehicle
Lactate
kDa
RACK1
β-actin
25
40
actin
RACK1
actin
RACK1

S7C

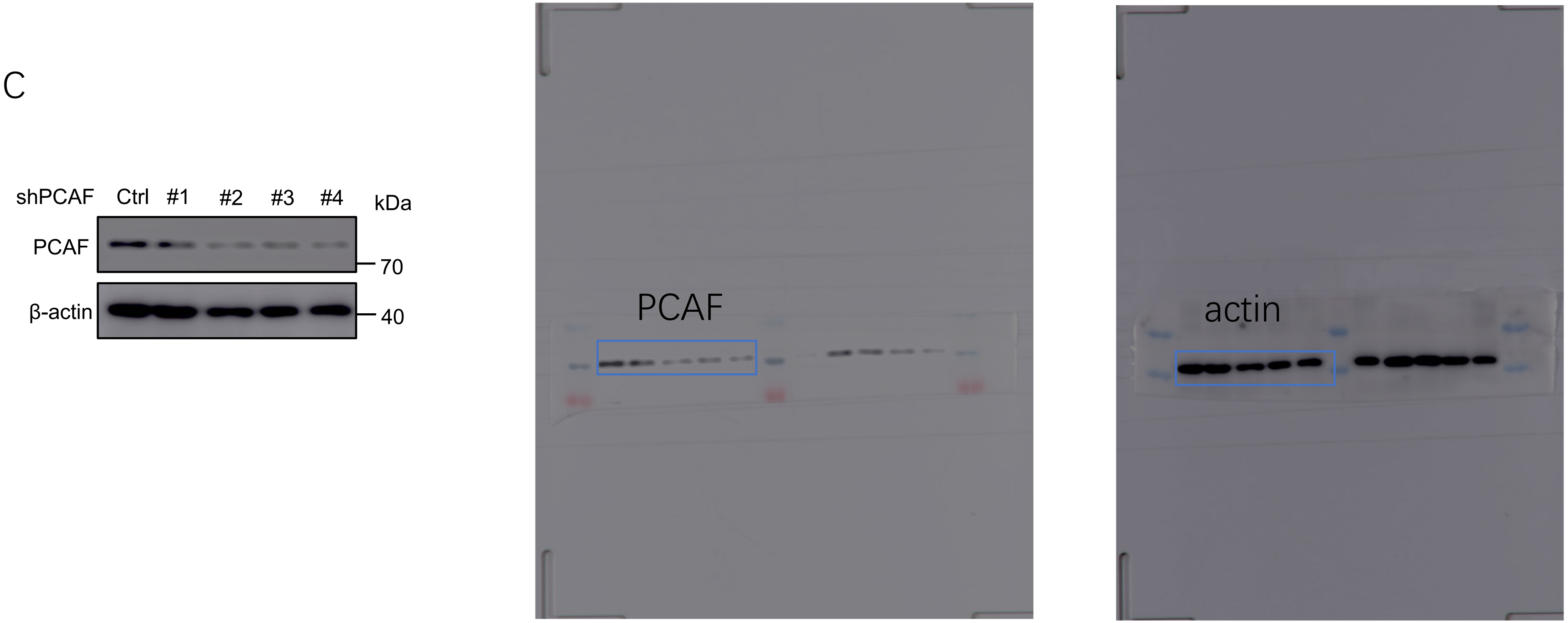
shPCAF
Ctrl
#1
#2
#3
#4
kDa
PCAF
β-actin
70
40
PCAF
actin

S8C

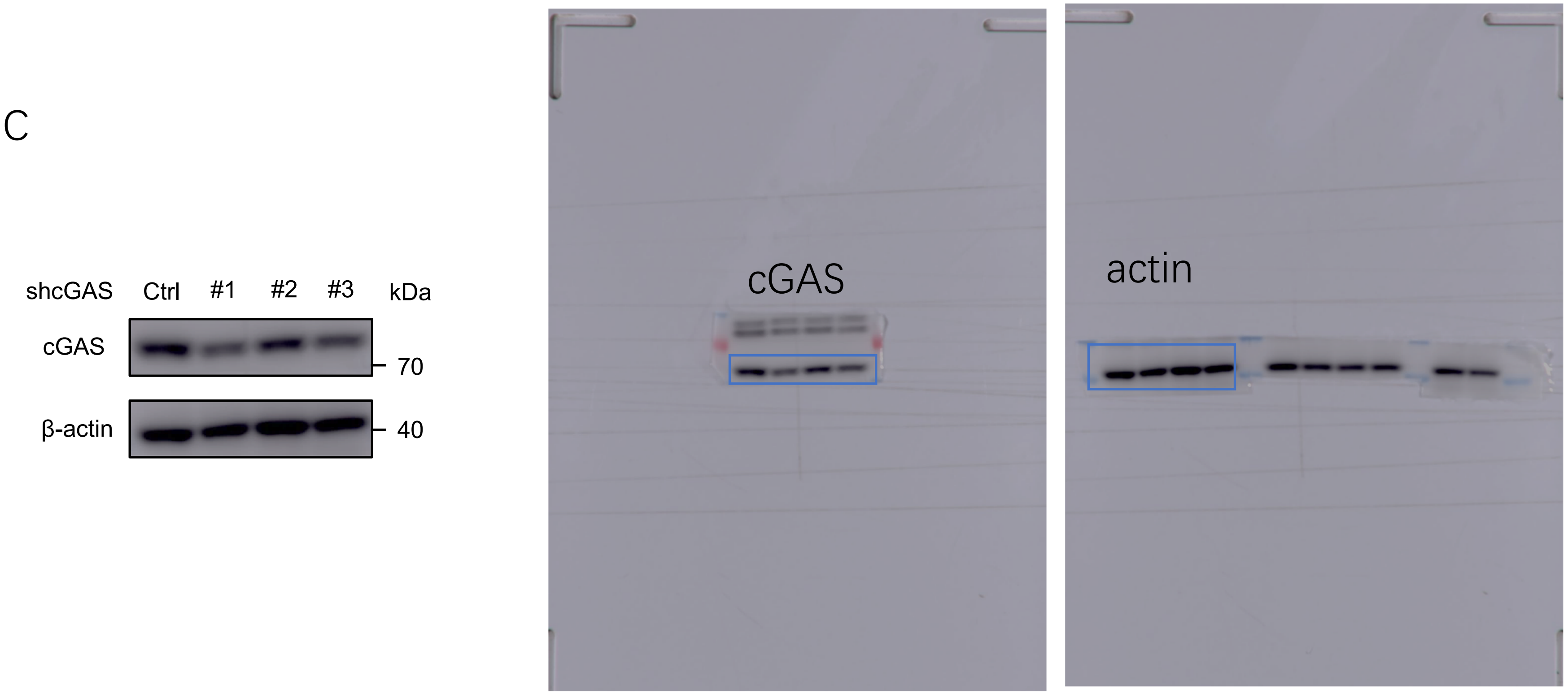
shcGAS
Ctrl
#1
#2
#3
kDa
cGAS
70
β-actin
40
cGAS
actin

S10A

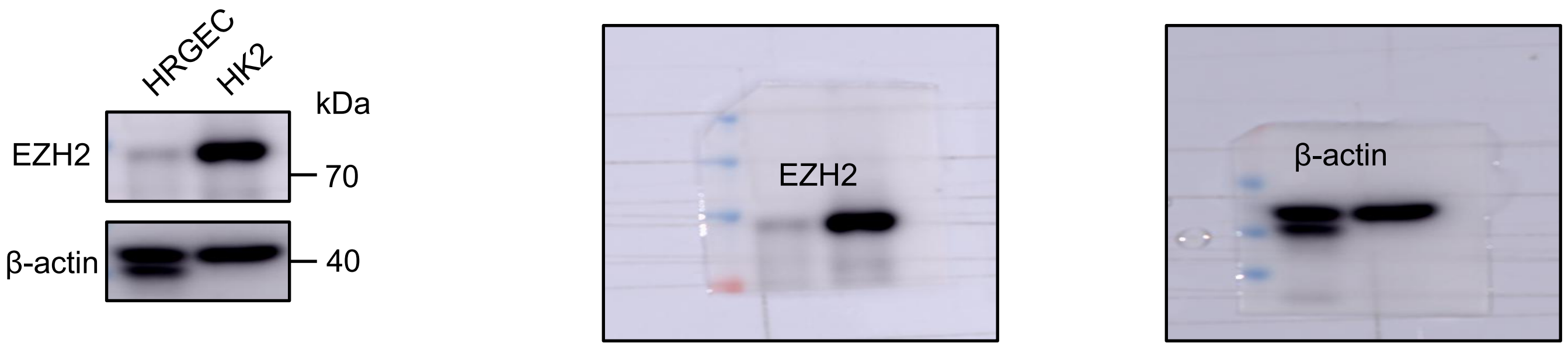
HRGEC
HK2
kDa
EZH2
70
β-actin
40
EZH2
β-actin

S10B

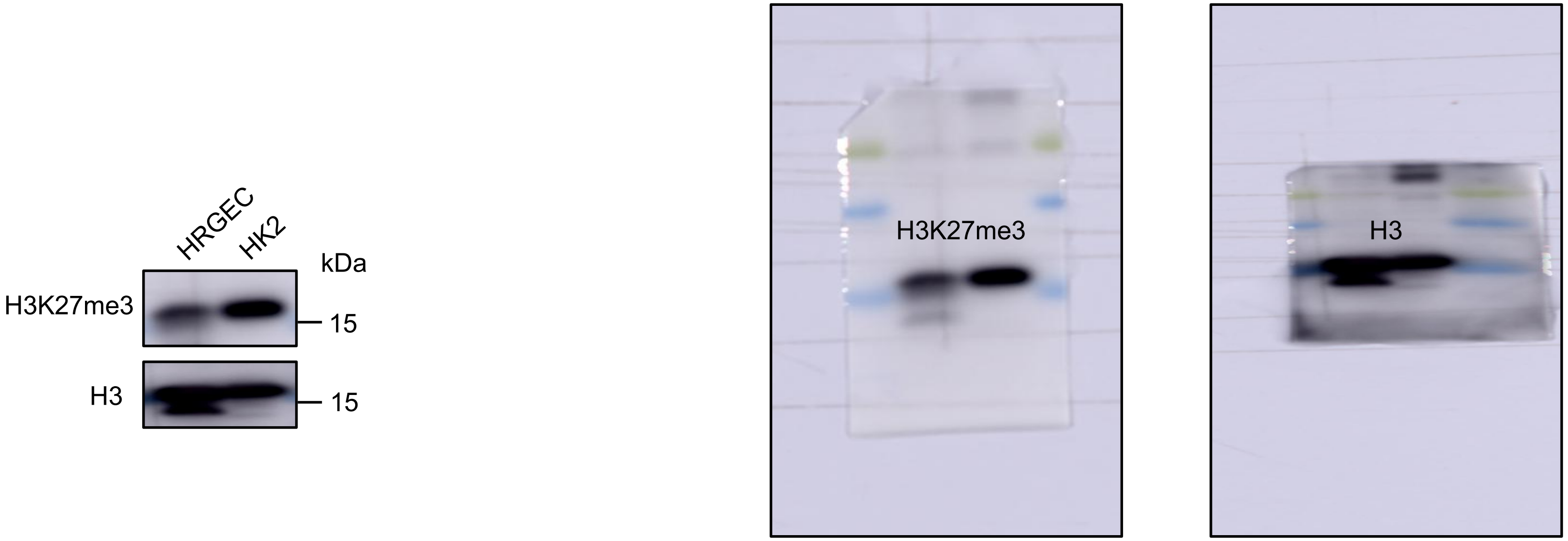
HRGEC
HK2
kDa
H3K27me3
15
H3
15
H3K27me3
H3

S10C

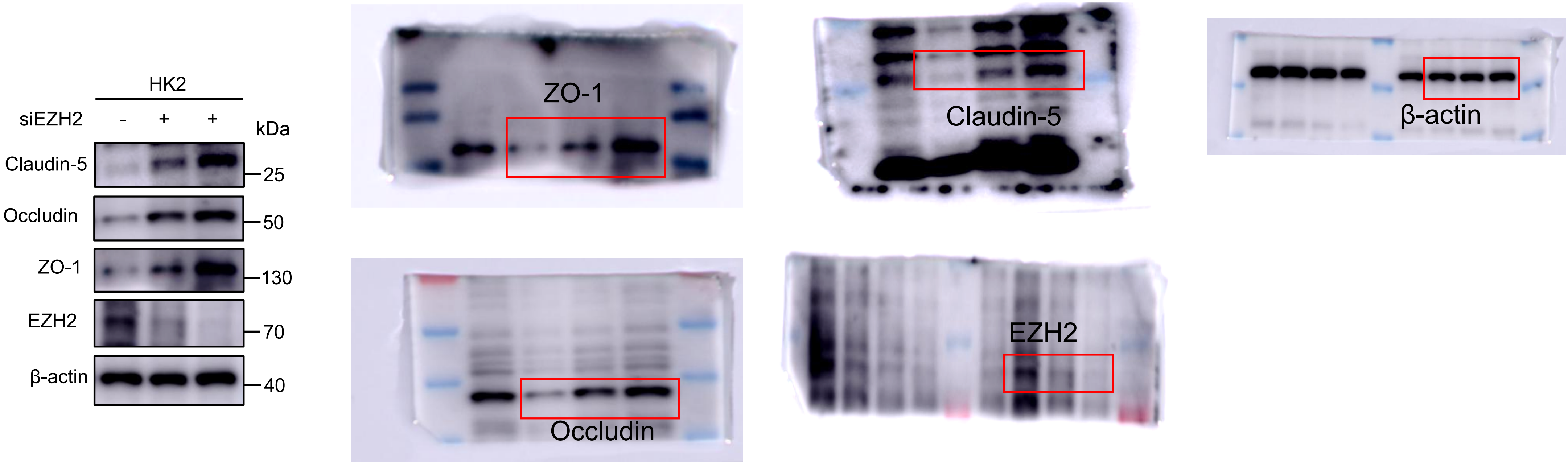
HK2
siEZH2
-
+
+
kDa
Claudin-5
25
Occludin
50
ZO-1
130
EZH2
70
β-actin
40
ZO-1
Claudin-5
β-actin
Occludin
EZH2

S10E

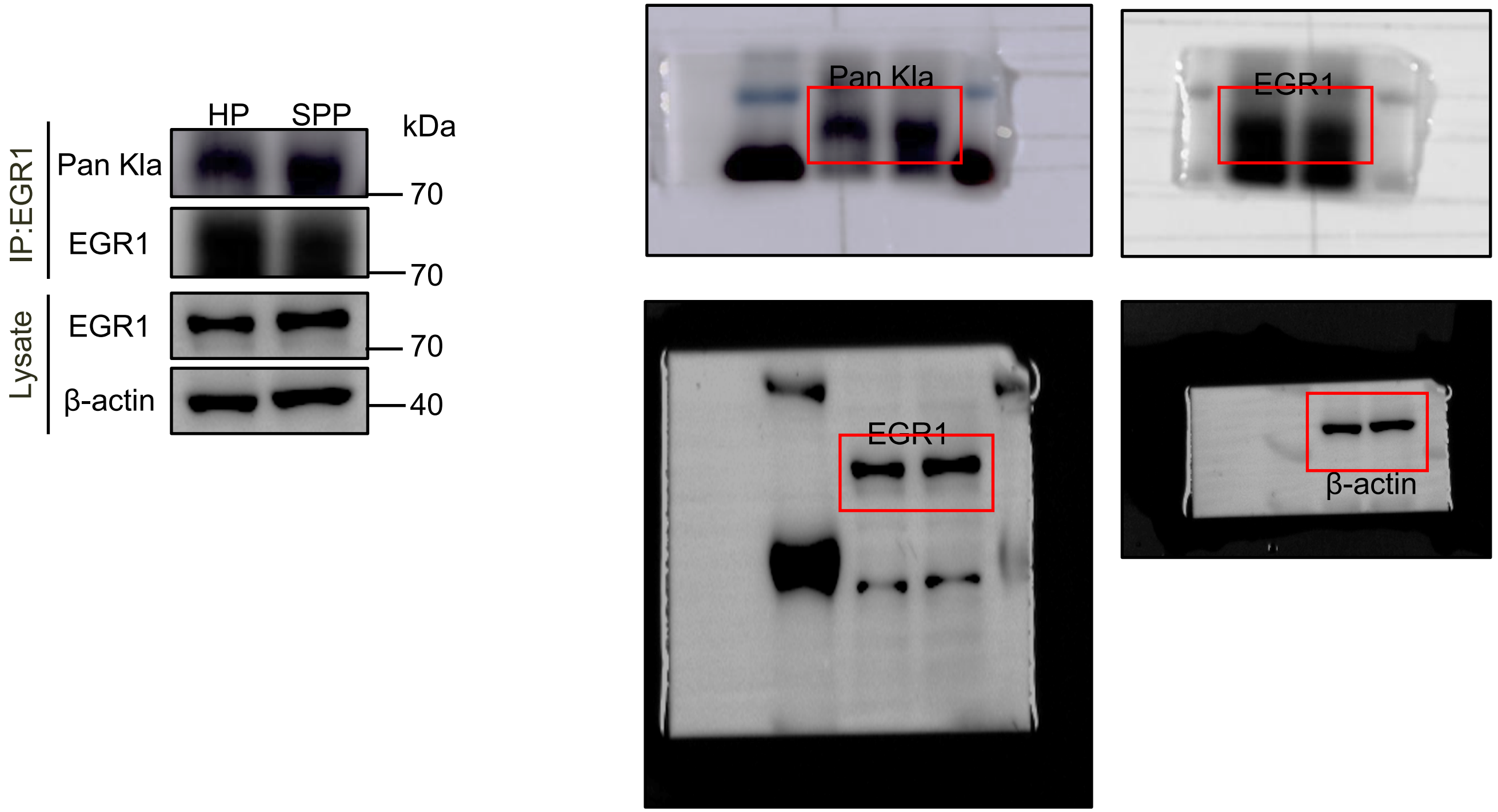

HP
SPP
kDa
IP:EGR1
Pan Kla
EGR1
70
70
Lysate
EGR1
β-actin
70
40
Pan Kla
EGR1
EGR1
β-actin
